# Paraptotic Cell Death as an Unprecedented Mode of Action Observed for New Bipyridine-Silver(I) Compounds Bearing Phosphane Coligands

**DOI:** 10.1021/acs.jmedchem.3c01036

**Published:** 2024-02-24

**Authors:** Ricardo G. Teixeira, Alessia Stefanelli, Adhan Pilon, Rebecca Warmers, Xavier Fontrodona, Isabel Romero, Paulo J. Costa, Maria J. Villa de Brito, Xenia Hudec, Christine Pirker, Sebastian Türck, Alexandra M. M. Antunes, Christian R. Kowol, Ingo Ott, Anamaria Brozovic, Andy Sombke, Margret Eckhard, Ana Isabel Tomaz, Petra Heffeter, Andreia Valente

**Affiliations:** † Centro de Química Estrutural, Institute of Molecular Sciences, Departamento de Química e Bioquímica, Faculdade de Ciências, Universidade de Lisboa, Campo Grande, Lisboa 1749-016, Portugal; ‡ Center for Cancer Research and Comprehensive Cancer Center, Medical University of Vienna, Vienna 1090, Austria; § Departament de Química and Serveis Tècnics de Recerca, Universitat de Girona, Campus de Montilivi, Girona 17071, Spain; ∥ BioISI - Instituto de Biosistemas e Ciências Integrativas, Faculdade de Ciências, Universidade de Lisboa, Lisboa 1749-016, Portugal; ⊥ Institute of Inorganic Chemistry, Faculty of Chemistry, University of Vienna, Waehringerstrasse 42, Vienna 1090, Austria; # Centro de Química Estrutural (CQE), Institute of Molecular Sciences, Departamento de Engenharia Química, Instituto Superior Técnico (IST), Universidade de Lisboa, Av Rovisco Pais 1, Lisboa 1049-001, Portugal; ∇ Institute of Medicinal and Pharmaceutical Chemistry, Technische Universität Braunschweig, Beethovenstr. 55, Braunschweig 38106, Germany; ○ Division of Molecular Biology, Ruđer Bošković Institute, Bijenička cesta 54,Zagreb 10000, Croatia; ◆ Center for Anatomy and Cell Biology, Cell and Developmental Biology, Medical University of Vienna, Schwarzspanierstraße 17, Vienna 1090, Austria

## Abstract

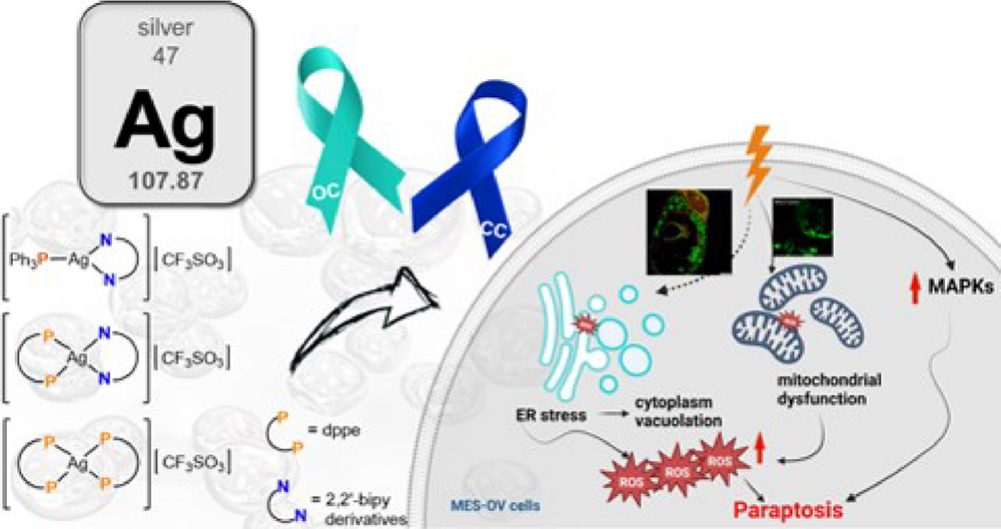

In this work, we investigated the anticancer activity of several novel silver(I) 2,2′-bipyridine complexes containing either triphenylphosphane (PPh_3_) or 1,2-bis(diphenylphosphino)ethane (dppe) ligands. All compounds were characterized by diverse analytical methods including ESI-MS spectrometry; NMR, UV–vis, and FTIR spectroscopies; and elemental analysis. Moreover, several compounds were also studied by X-ray single-crystal diffraction. Subsequently, the compounds were investigated for their anticancer activity against drug-resistant and -sensitive cancer cells. Noteworthily, neither carboplatin and oxaliplatin resistance nor p53 deletion impacted on their anticancer efficacy. MES**-**OV cells displayed exceptional hypersensitivity to the dppe-containing drugs. This effect was not based on thioredoxin reductase inhibition, enhanced drug uptake, or apoptosis induction. In contrast, dppe silver drugs induced paraptosis, a novel recently described form of programmed cell death. Together with the good tumor specificity of this compound’s class, this work suggests that dppe-containing silver complexes could be interesting drug candidates for the treatment of resistant ovarian cancer.

## 1 Introduction

Cancer is the second leading cause of death worldwide with 19.3 million new cases and 10 million deaths in 2020.^1^ One of the most common cancers among women is ovarian cancer (OC).^2^ Usually, OC is diagnosed at an advanced/late stage, resulting in poor prognosis of the patients. The first-line OC treatment is based on a combination regimen of surgery followed or preceded by platinum-based chemotherapy (cisplatin or carboplatin) and paclitaxel. However, rapid disease recurrence (of drug-resistant clones) is one of the major handicaps of platinum-based chemotherapy. Therefore, additional maintenance therapy with poly(ADP-ribose) polymerase (PARP) inhibitors drugs such as olaparib, rucaparib, or niraparib is currently recommended.^3,4^ In general, progression-free survival has been enhanced up to 15.5 months with PARP inhibitors. Nevertheless, these drugs do not solve the problem of low survival rates of OC patients.^5^ Consequently, in the quest for new compounds to treat OC with acquired resistance, new drugs that are not limited by (platinum) resistance are needed. Several silver complexes have been reported to exert promising anticancer activity against resistant OC.^6,7^ In general, silver compounds, comparable to their gold counterparts, are developed because of their promising antimicrobial and anticancer activity.^7−9^ Noteworthily, in contrast to the frequently observed in vivo toxicity of gold complexes, silver drugs are supposed to be better tolerated as a result of the inherent lack of toxicity of silver itself.^10,11^

Different silver(I) complexes have been synthesized over the years, listed in four different classes: Ag(I)-carboxylate, Ag(I)-N-ligand, Ag(I)-P-ligand, and Ag(I)-mixed ligand complexes.^12^ With regard to their mode of action, little is known. Recent studies have demonstrated that in the case of Ag(I)-NHCs (silver(I)-*N*-heterocyclic carbenes), the thiol oxidoreductase as well as the thioredoxin reductase systems might be important intracellular targets.^7^ In addition, cell cycle arrest and reactive oxygen species (ROS) production have been reported for certain silver complexes.^13−15^ In this study, we present the synthesis and biological evaluation of several new silver compounds concomitantly bearing phosphanes (triphenylphosphane (PPh_3_) or 1,2 bis(diphenylphosphino)ethane (dppe)) and 2,2′-bipyridine-based ligands (bipy). We chose to develop this family of compounds because, as far as we are aware, these structures have never been evaluated for their anticancer potential. Our results demonstrate that the compounds are not only active in cell lines with a carboplatin/oxaliplatin-resistance phenotype but also especially promising against OC by induction of a novel form of cell death called paraptosis.

## 2 Results and Discussion

### 2.1 Synthesis and Characterization

**Figure 1:**
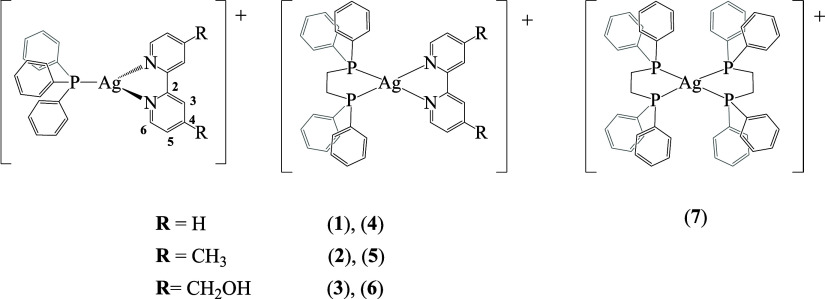
Chemical structures of compounds **1**–**7**. All compounds were isolated as CF_3_SO_3_^–^ salts.

Six silver(I) mononuclear complexes with the general formula [Ag(bipy)(P)][CF_3_SO_3_] (Figure 1) incorporating bipyridine-based ligands (2,2′-bipyridine (**1**, **4**), 4,4′-dimethyl-2,2′-bipyridine (**2**, **5**), and 4,4′-bis(hydroxymethyl)-2,2′-bipyridine (**3**, **6**)) were prepared by reacting silver trifluoromethanesulfonate with the corresponding bipyridine derivative and PPh_3_ (**1**–**3**) or dppe (**4**–**6**) in a 1:1:1 molar ratio at room temperature. Compound **7** was synthesized in dichloromethane at room temperature with silver trifluoromethanesulfonate and dppe 1:2. All new compounds were isolated by slow diffusion recrystallization in very good to excellent yields and fully analyzed by ^1^H, ^13^C{^1^H}, ^31^P{^1^H}-NMR, FTIR, and UV–vis spectroscopies and mass spectrometry (see SI). Elemental analyses confirmed the stoichiometry and purity of all compounds. In addition, single crystals of compounds **1**, **2**, **3**, and **4** were successfully obtained and studied by single-crystal X-ray crystallography. The air-stable white solids obtained are insoluble in water, diethyl ether, and *n*-hexane but are soluble in chlorinated solvents (such as dichloromethane and chloroform), acetone*, acetonitrile*, and DMSO (*compounds **4**–**6** are not soluble in this solvent).

The solid-state Fourier-transform infrared spectroscopy (FTIR) spectra (in KBr pellets) confirmed, in all cases, the presence of the triflate anion with the characteristic bands at 1250, 1160, and 1030 cm^–1^. Compounds **1**–**7** also present strong absorption of stretching bands ascribed to the υ(C–H) and υ(C=C) aromatic rings of the phosphane and bipyridine ligands in the expected ranges (3200–3000 and 1600–1400 cm^–1^). In addition, compounds **3** and **6** present broad bands between 3367 and 3440 cm^–1^ due to the presence of the hydroxyl group at the hydroxymethylated bipyridine ligand.

Electronic absorption spectra of compounds **1**–**7** were recorded at room temperature using ∼10^–4^ to 10^–5^ M DMSO solutions (Figures S1 and S2). Typically, the optical absorption spectra of the compounds showed a similar behavior and are dominated by intense absorption bands at high energy values (λ_max_ ∼280 nm) that are assigned to the intraligand π → π* transitions centered on the coordinated chromophores.

**Figure 2:**
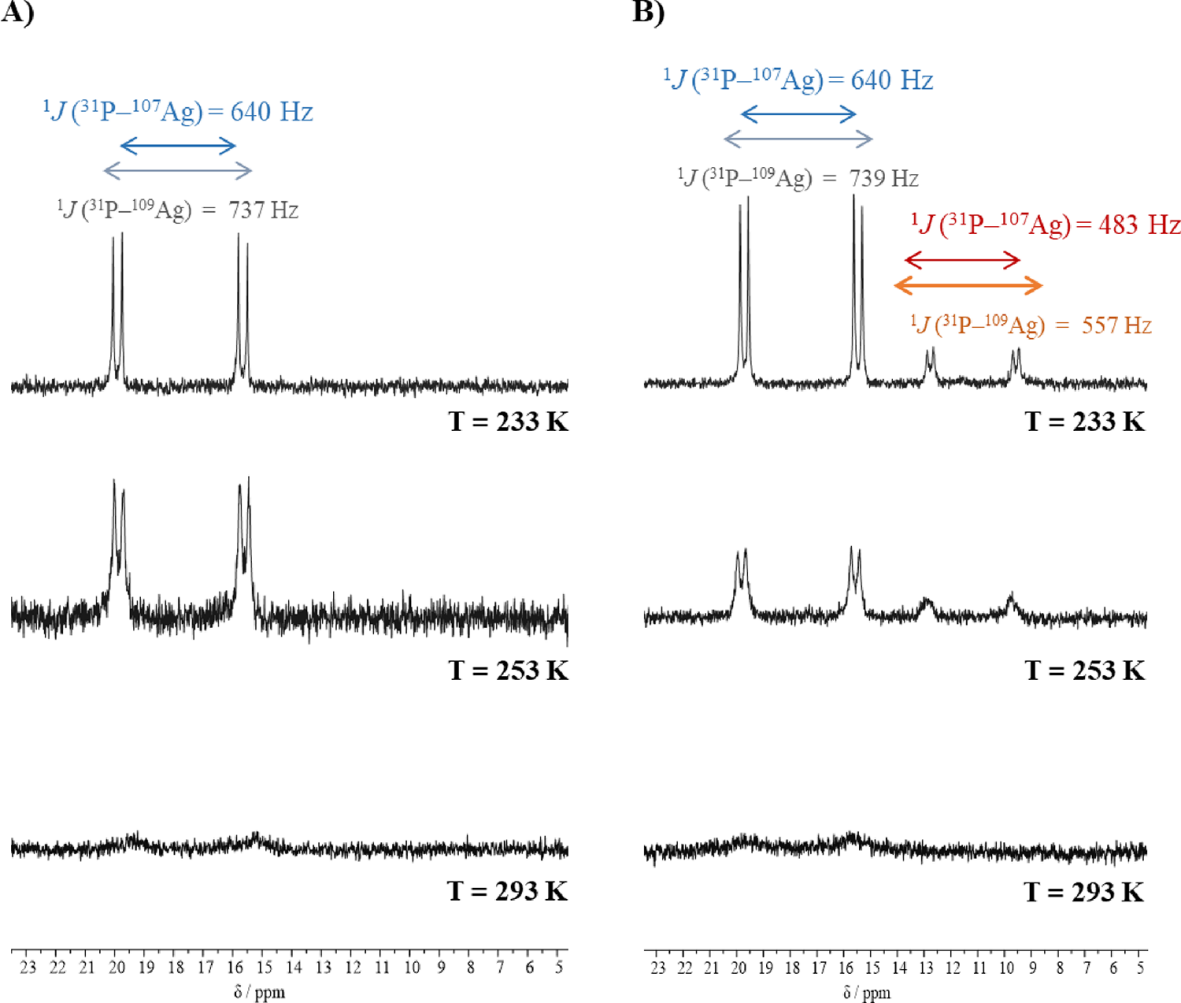
^31^P{^1^H} NMR spectra (162 MHz, CDCl_3_) of the PPh_3_-containing compounds **2** (A) and **3** (B) at different temperatures.

**Scheme 1:**
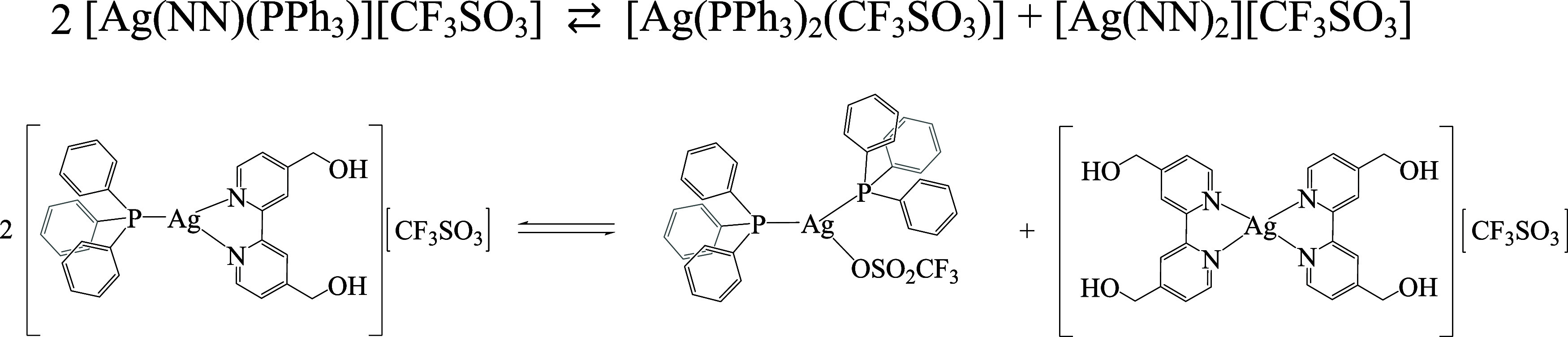
Depictive Reaction Equilibrium Detected for Complex **3** to Yield the Additional AgP_2_ Species in the Solution (CDCl_3_) Observed by NMR and MS

Compounds **1**–**7** were also characterized by NMR spectroscopy through ^1^H, ^13^C{^1^H}, and ^31^P{^1^H} (Figures S3–S23) and bidimensional correlation experiments (COSY, HMQC, and HMBC) in DMSO-*d*_6_ to unequivocally attribute all compounds’ resonances. The coordination of both *N,N*-bidentate and mono- or bidentate *P*-donors was confirmed by ^1^H and ^31^P{^1^H} NMR, and the spectra of all compounds show a common trend: the aromatic protons of the bipyridine shift to downfield values (with respect to the free ligands), with the greatest shift being observed for the H5 protons of PPh_3_ (Δδ H5 ∼ 0.23 ppm) and dppe-containing compounds (Δδ H5 ∼ 0.07 ppm), respectively. As found in many examples in the literature,^16^ small shifts on the ^1^H resonances seem to be typical of silver compounds. The presence of the methyl and hydroxymethyl substituents at the bipyridine ligand was also easily identified in their respective spectral range as they were not significantly affected upon coordination to the metal center. Although the aromatic protons of the phosphanes follow the same tendency, they could not be completely distinguished because of the overlap of the signals. However, all resonances ascribed to their *ortho-*, *meta-,* and *para-*protons are found in the characteristic range (7.70 ppm < H_*ortho*, *meta*, *para*_ < 7.20 ppm). The analysis of the ^13^C{^1^H} spectra corroborate the previous discussion. The changes in electron density upon coordination to the metal center had a significant impact on the phosphorus nuclei. The ^31^P{^1^H} NMR spectra in DMSO-*d*_6_ for compounds **4**–**7**, recorded at room temperature, show two doublets centered at δ = ∼3.4 ppm due to the coupling of the phosphorus atoms to both ^107^Ag and ^109^Ag nuclei, with ^1^*J*(^31^P–^107^Ag) ∼230 Hz and ^1^*J* (^31^P–^109^Ag) ∼266 Hz, assigned in accordance to the direct relation between the ratio of the coupling constants and the ratio of the magnetogyric ratios of the silver isotopes (∼1.15). These results are in agreement with values reported in the literature.^17,18^ In the case of compounds **1**–**3**, the ^31^P{^1^H} NMR spectra in DMSO-*d*_6_ recorded at room temperature show one broad resonance, presumably due to the relatively fast dynamic exchange in comparison to the NMR time scale, which led to further studies of these systems with variable temperature nuclear magnetic resonance (VT-NMR) experiments in CDCl_3_. At low temperature, this exchange equilibrium was quenched, and well-resolved pairs of doublets were observed for compounds **1**, **2**, and **3** as a result of phosphorus coupling to individual ^107^Ag and ^109^Ag isotopes, similarly to the dppe-containing compounds. As an example, the effect of cooling a CDCl_3_ solution of compounds **2** and **3** is depicted in Figure 2A,B, respectively. At 233 K, the ^31^P{^1^H} NMR spectra are resolved with spin–spin coupling constants lying near 640 Hz for ^1^*J*(^107^Ag–^31^P) and 740 Hz for ^1^*J*(^109^Ag–^31^P). In the low-temperature ^31^P{^1^H} spectrum of compound **3** (Figure 2B), two pairs of doublets are detected at δ 17.6 ppm (with ^1^*J*(^107^Ag–^31^P) ∼640 Hz and ^1^*J*(^109^Ag–^31^P) ∼739 Hz) and at δ 11.2 ppm (with ^1^*J*(^107^Ag–^31^P) ∼483 Hz and ^1^*J*(^109^Ag–^31^P) ∼557 Hz). The latter is in accordance with the presence of additional AgP_2_-containing species, presumably formed as a result of the dissociation equilibrium shown by Scheme 1, which was confirmed by mass spectrometry (*m*/*z* = 630.92 ⟨=⟩ {[Ag(PPh_3_)_2_]^+^}). In all cases, the coupling constants are in accordance with an AgN_2_P coordination environment for compounds **1**–**3**, which are in perfect agreement with their solid-state structures (see single-crystal X-ray discussion).

**Figure 3:**
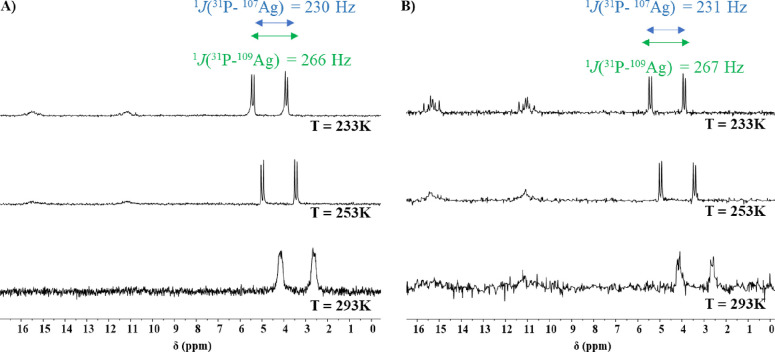
^31^P{^1^H} NMR spectra (162 MHz, CDCl_3_) of the dppe-containing compounds **5** (A) and **6** (B) at different temperatures.

VT-NMR studies were also performed for compounds **4**–**6** in CDCl_3_. For compound **6**, we only observed an improved resolution of the spectra. Yet, for compounds **4** and **5**, at 233 K, a new signal appeared in the ^31^P NMR spectra at δ = 13.30 ppm (Figure 3) whose pattern is consistent with an [Ag_2_(NN)_2_(μ-dppe)_2_]^2+^ type complex. The doublet of multiplets observed (better resolved in B) can result from a distribution of isotopologues^19^ (^109^Ag-^109^Ag, ^109^Ag-^107^Ag, and ^107^Ag-^107^Ag) close to 1:2:1, as the isotopic ratio for the two spin isotopes ^109^Ag and ^107^Ag (natural abundance 51.8 and 48.2%, respectively) is close to 1:1. A similar behavior with silver(I) complexes bearing diphosphane ligands has been reported before.^20^

In all cases, the ratio between the ^31^P to Ag nuclei coupling constants (^1^*J*(^31^P-^109^Ag)/^1^*J*(^31^P-^107^Ag) ≃ 1.15 is in good agreement with the quotient between the magnetogyric ratios of the corresponding silver isotopes (γ (^109^Ag) /γ (^107^Ag) = 1.1498),^21^ supporting the proposed assignment in the ^31^P{^1^H} NMR spectra.

#### 2.1.1 Single-Crystal X-ray Studies

**Figure 4:**
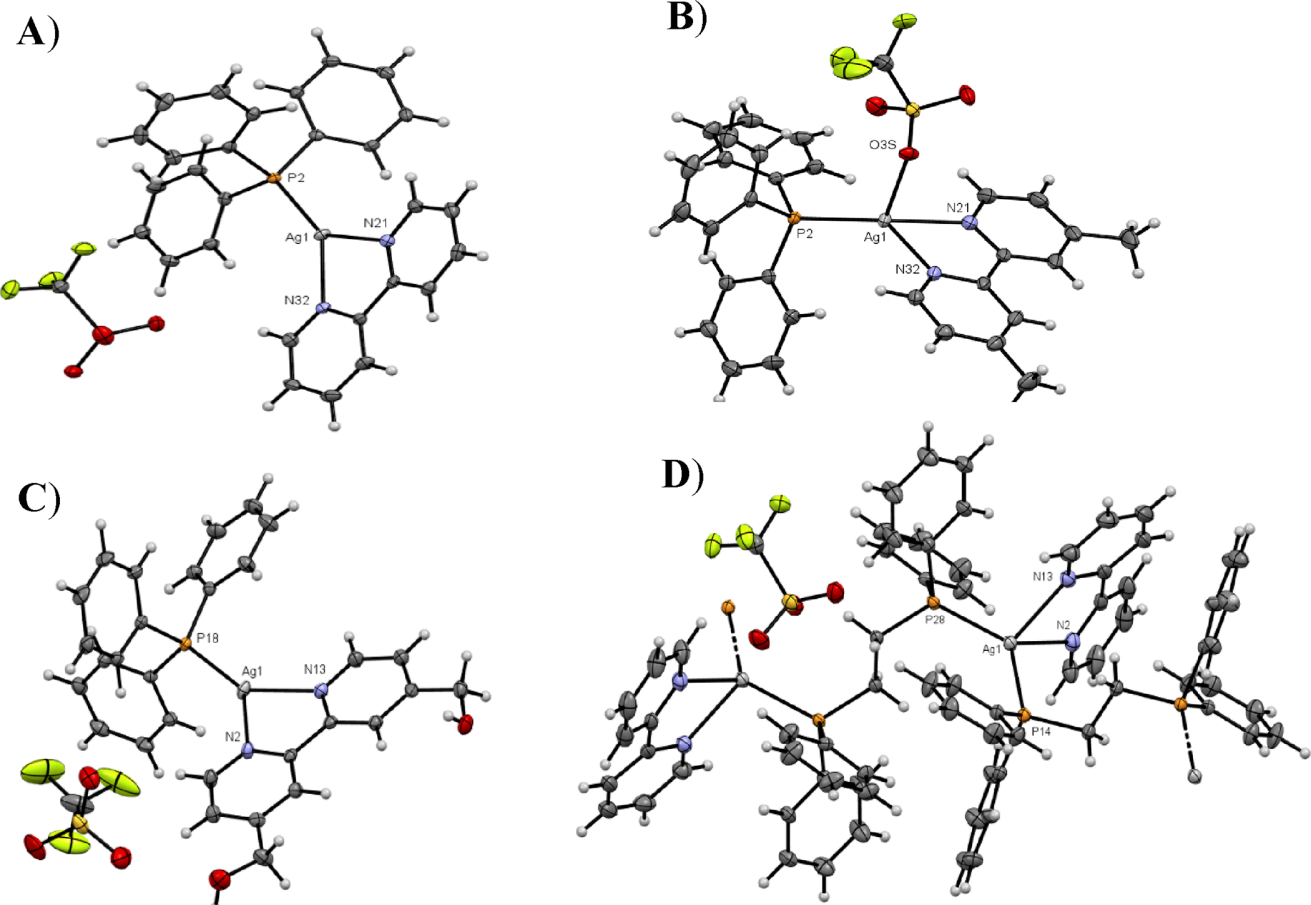
ORTEP plots (50% probability) and labeling schemes for compounds **1** (A), **2** (B), **3** (C), and **4** (D). Solvent molecules and disordered atoms are omitted for clarity.

The crystal structures of complexes **1**–**4** have been solved by X-ray diffraction analysis. Crystal data for these complexes are summarized in the Experimental Section, and the selected bond lengths and angles are listed in Table S1. Figure 4 displays the ORTEP diagrams of their molecular structures.

Complexes **1** and **3** crystallize in the triclinic system space group *P*-1 and display similar structures. Both contain a tricoordinated silver(I) ion with a distorted trigonal planar geometry, highlighting the geometrical restrictions imposed by the bipyridyl ligands (Figure 4A,C). As expected, the silver(I) cations are coordinated by one phosphorus atom of the triphenylphosphane ligand and by two nitrogen atoms from the corresponding bidentate bipyridine ligands, which act as chelating ligands. The functionalization of the bipyridine ligand with −CH_2_OH substituents in complex **3** has only a minor impact on the molecular structures. The Ag–P and Ag–N distances and associated angles are similar in both complexes, although the bond distances are slightly longer in complex **1** (Table S1). All bond distances and angles are within the expected values for this trigonal silver(I) complexes.^22,23^ Complexes **1** and **3** also display nonclassical intramolecular hydrogen bonds between the fluoride and oxygen atoms of the triflate anions and the hydrogen atoms of the bipy ligands and the hydrogen atoms of the phenyl groups of the triphenylphosphane ligand (H31–O2R = 2.618 Å; H19–O2R = 2.738 Å, and H14–F6R = 2.732 Å in **1**; H3–O2S = 2.577 Å and H36–O2S = 2.804 Å in **3**; see Figure S24A). In the case of **3**, two extra intramolecular interactions were observed between the oxygen atoms of the −CH_2_OH groups and the neighboring hydrogens of the pyridyl rings (H9–O17 = 2.473 Å and H4–O15 = 2.514 Å, see Figure S24B). The packing of **1** and **3** along the *a* axis shows the anions CF_3_SO_3_^–^ located between the silver cations. Also, π-stacking interactions are observed between bipyridine rings of neighboring molecules (Figure S25A,B).

Complex **2** crystallizes in the space group *P*12_1_/c 1 and displays a silver cation in a distorted tetrahedral environment with the bipyridine, triphenylphosphane, and triflate ligands bonded to the central atom through the N(21) and N(32), the P(2), and the O(3′) atoms, respectively. The Ag–N and Ag–P distances are of the same order of magnitude (2.3–2.34 Å), and the values are similar to other complexes described in the literature.^20,24^ However, the Ag–O bond distance is larger (2.501 Å) than the Ag–N and Ag–P distances and readily dissociates in solution (Figures S26 and S27). Similar to the structures of **1** and **3**, the distortion of the tetrahedral geometry around the silver cation is due to the restrictions imposed by the bipyridine bite angle N(21)–Ag(1)–N(32) of 71.88(8)°. The packing structure of complex **2** shows π-stacking interactions between two pyridine rings of two neighboring bipyridine ligands (Figure S25D).

Complex **4** crystallizes in the space group *P*12_1_/*n*1, and surprisingly, the crystal X-ray structure displays the formation of a polymeric compound where the basic repeat unit includes the silver(I) cation, the bipyridine, and the 1,2-bis(diphenylphosphino)ethane ligands. The polymer can be described as a 1D linear chain of alternating silver(I) cations and bridging diphosphane ligands linked through the phosphorus atoms. This infinite 1D chain is generated by two inversion centers located at C27–C27# and C41–C41#. Each silver(I) is coordinated by two nitrogen atoms from the bipyridine ligand and by two phosphorus atoms from two different diphosphane units, showing a distorted tetrahedral geometry attributed to the bipyridine bite angle N(32)–Ag(1)–N(21) of 71.88(8), quite similar to what is observed in other silver polymers.^25^ The Ag–P bond distances are similar (Ag(1)–P(14), 2.4302(15) and Ag(1)–P(28), 2.4325(18)) and lie within the range of those found in other linear silver(I) polymers.^26,27^ The packing along the *a* axis shows linear chains with the anions CF_3_SO_3_^–^ and solvent molecules situated between the different chains (Figure S25E). However, no π-stacking interactions between aromatic rings of neighboring molecules have been observed. Another view of the packing is shown in Figure S25F.

#### 2.1.2 DFT Calculations

**Figure 5:**
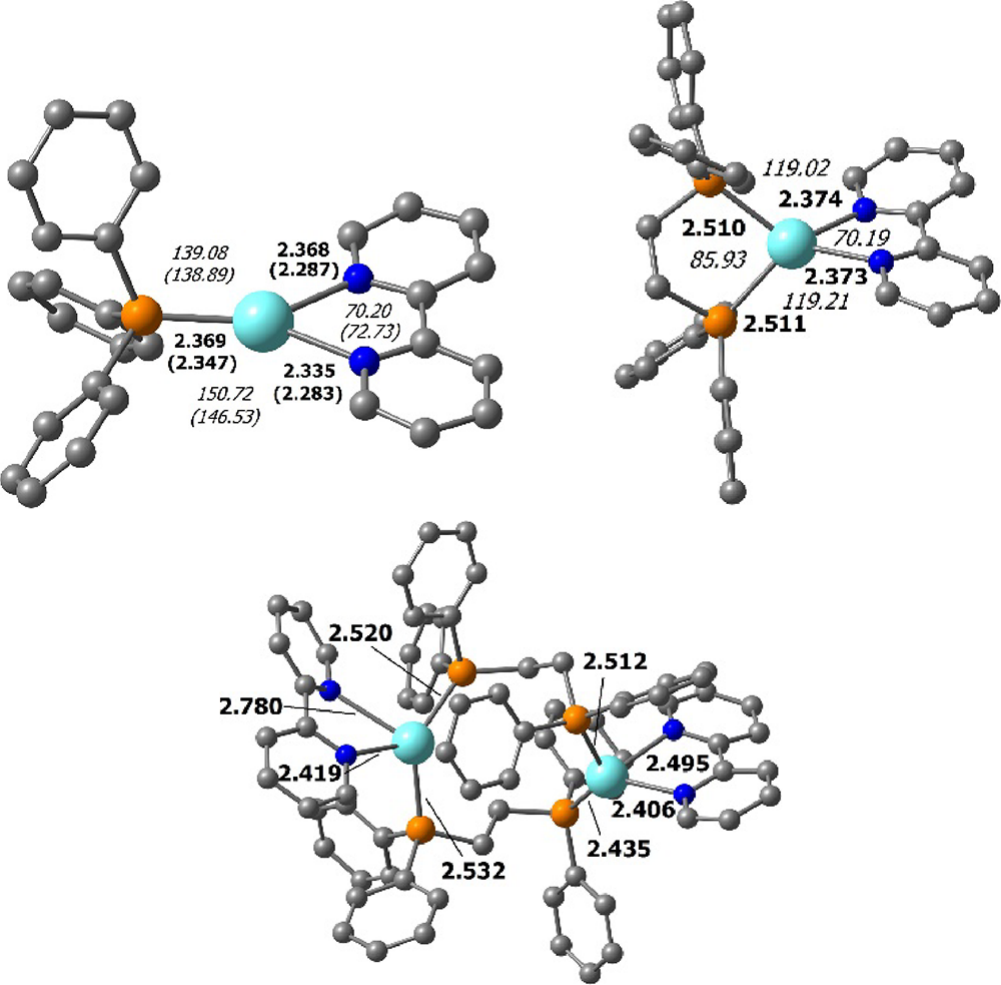
DFT optimized structures of complexes **1** (top, left) and **4** (top, right). The optimized structure of the proposed complex formed by **4** at low temperatures by VT-NMR studies ([Ag_2_(NN)_2_(μ-dppe)_2_]^2+^, 4*) is shown on the bottom. Hydrogen atoms are omitted for clarity. Experimental X-ray values are given in parentheses. Distances (bold) are given in angstroms, and angles (italics) are given in degrees.

Using the experimental X-ray data as a starting point, the geometry of complex **1** was optimized at the DFT level of theory (M06L functional,^28^ see computational details) and depicted in Figure 5 (top, left). The distorted trigonal coordination sphere of silver observed in the X-ray structure is properly described in the optimized geometry as the N–Ag–N angle is obviously smaller than the P–Ag–N angles that, on the other hand, are also not similar: 150.7 and 139.1° (calculated), 146.5 and 138.9° (X-ray). A satisfactory agreement is observed for the most important bond lengths, although a slight overestimation of the Ag–P and Ag–N bond lengths is observed. This is acceptable because the X-ray structure is obtained in the solid state, whereas the optimized structures were obtained in a vacuum. For complexes of the [Ag(4,4′-R-2,2′-bipy)(dppe)]^+^ family (**4**–**6**), only one crystal structure could be obtained (complex **4**), which surprisingly presents a polymeric compound. Therefore, to structurally characterize the geometry of complex **4** present in the solution (as per NMR data, see above), a geometry was also optimized (Figure 5, top, right). As expected for a d^10^ species, the coordination geometry around silver is tetrahedral, though distorted, and an elongation of both Ag–N and Ag–P bonds is observed when going from complex **1** to **4**. At low temperatures, the NMR of complex **4** shows the formation of a species, assigned as [Ag_2_(NN)_2_(μ-dppe)_2_]^2+^ (henceforth called 4*).^20^ The optimized structure of such a complex is shown in Figure 5 (bottom). As in **4**, the dimeric species 4* conserves the tetrahedral arrangement. However, it is highly distorted owing to the strain on the bridging phosphane ligand. This strain also imposes the elongation of both Ag–N and Ag–P bonds and additionally, also explains why structures featuring an Ag···Ag contact could not be located.

Overall, even though it was not possible to create a straight correlation between the solution and solid-state behavior of the dppe-containing compounds **4**–**6**, our data reveal that, in solutions, the mononuclear species (where the PP bidentate ligand forms a five-membered chelate ring with the metal ion) is the prevalent one.

### 2.2 Biological Evaluation of the New Silver Compounds

#### Anticancer Activity of the New Silver Compounds against Chemosensitive and Platinum-Resistant Cancer Cells as well as a Nontumorigenic Cell Model

Prior to the cytotoxic evaluation of the compounds, their stability in DMSO-*d*_6_ was monitored over 48 h by ^1^H and ^31^P{^1^H} NMR experiments at room temperature (Figures S28 and S29). According to data collected, the compounds were very stable over the assay period. As such, we then analyzed the stability of compounds **1**, **4**, and **7** (representative compounds of each subfamily) in PBS by LC-ESI(+)-HRMS/MS analysis. The results obtained clearly showed that **7** is stable under these conditions (Figure S32). For compound **1**, it was observed, similarly to what had already been observed by NMR in organic solvents (Section 2.1), that once in solution, an equilibrium is established between the parent complex and AgP_2_ and AgN_2_ species (Scheme 1), which remains stable throughout the duration of the assay (Figures S30 and S34). For compound **4**, an immediate rearrangement of the ligands to afford a mixture between AgP_2_ and AgN_2_ species is observed, which remains stable throughout the duration of the assay (Figures S31 and S33).

The cytotoxicity and the cross-resistance profile of the seven silver(I) 2,2′-bipyridine derivatives containing either PPh_3_ or dppe ligands was tested against two OC models (SK-OV-3 and MES-OV), one colon carcinoma (HCT-116), and their respective carboplatin/oxaliplatin-resistant counterparts (previously characterized^29−31^ SK-OV-3/CBP, MES-OV/CBP, and HCT116 WT/OXR) as well as nonmalignant fibroblasts (F331). To evaluate the impact of p53, an isogenic HCT-116 clone with p53 deletion based on artificial gene disruption (HCT-116 p53KO) was also included. MTT assays were performed after 72 h (Figures S35 and S36), and IC_50_ values were calculated (Tables 1 and 2). As a general trend, the silver(I) complexes revealed high anticancer activity in the low micromolar range with some exceptions, where nanomolar activity was reached. Moreover, there was a distinct difference between compounds bearing PPh_3_ (**1**–**3**) and dppe (**4**–**7**) ligands. In more detail, all PPh_3_-containing compounds had IC_50_ values in cancer cells around 2–4 μM, whereas nonmalignant F331 cells were less sensitive (IC_50_ values ∼8 μM). In contrast, the sensitivity to the dppe-containing drugs varied strongly. In the case of SK-OV-3, IC_50_ values between 4.3 and 7.2 μM were reached. The HCT-116 cells were more sensitive with IC_50_ values between 0.3 and 0.7 μM. An exceptional sensitivity was observed in MES-OV cells, where the lowest tested concentration of 0.05 μM was already able to kill more than 50% of the cancer cells in most cases (compounds **4**, **6**, and **7**). On the opposite, the compounds were rather inactive (IC_50_ > 10 μM in most cases) in the nonmalignant F331 fibroblasts, indicating a very good cancer selectivity (Figure S37). This was further supported by a in parallel performed hemolytic assay (Figure S38). Noteworthily, none of the bipyridine ligand modifications had an impact on the anticancer activity. With regard to carboplatin and oxaliplatin resistance, none of the drugs were affected by the respective resistance mechanisms (compare Figures S35 and S36). In addition and in contrast to the reference drug oxaliplatin, loss of functional p53 did not render cells resistant to the silver compounds, thus indicating that DNA damage is not involved in their mode of action.

**Table 1:** Anticancer Activity (IC_50_ Values after 72 h) of the Silver Compounds in OC Models and Their Carboplatin-Resistant Counterparts as well as Nonmalignant Fibroblasts^a^

**IC**_**50**_**μM**^b^							
**compound**	**SK-OV-3**	**SK-OV-3/CBP**	**relative resistance**^c^	**MES-OV**	**MES-OV/CBP**	**relative resistance**^c^	**F331**
[Ag(PPh_3_)(bipy-**R**)]^**+**^							
**1**	2.15 ± 0.30	3.50 ± 0.66	1.6^n.s.^	2.60 ± 0.26	2.52 ± 1.15	1.0^n.s.^	8.33 ± 0.3
**2**	2.37 ± 0.32	3.66 ± 0.55	1.5^n.s.^	>5	3.81 ± 0.11		8.57 ± 0.5
**3**	2.22 ± 0.50	3.20 ± 0.3	1.4^n.s.^	2.95 ± 1.11	2.30 ± 1.62	0.8^n.s.^	7.76 ± 0.8
[Ag(dppe)(bipy-**R**)]^+^							
**4**	6.80 ± 1.41	5.48 ± 2.16	0.8^n.s.^	<0.05	0.06 ± 0.02		>10
**5**	6.86 ± 1.55	6.50 ± 1.29	0.9^n.s.^	0.06 ± 0.03	0.06 ± 0.03	1.0^n.s.^	8.52 ± 0.9
**6**	6.50 ± 1.66	5.52 ± 2.20	0.8^n.s.^	<0.05	<0.05		>10
**7**	7.24 ± 1.44	5.97 ± 1.42	0.8^n.s.^	<0.05	0.05 ± 0.02		5.78 ± 0.5
**carboplatin**	56.47 ± 15.38	89.93 ± 5.64	1.6*	44.33 ± 7.09	94.67 ± 9.24	1.7**	n.t.

a ***p ≤ 0.001; **p ≤ 0.01; *p ≤ 0.05. n.s., not significantly different, calculated by one-sample *t* test; n.t., not tested.

b IC_50_ values were calculated from concentration–response curves. Values are given as mean ± SD of three independent experiments performed in triplicates.

c Differences in sensitivity calculated by dividing the IC_50_ values of the resistant subline by those of the parental line.

**Table 2:** Anticancer Activity (IC_50_ Values after 72 h) of the Indicated Compounds in HCT116, the Isogenic P53-Deleted Subclone, as well as Its Respective Oxaliplatin-Resistant Counterpart^a^

**IC**_**50**_**μM**^b^					
**compound**	**HCT116 WT**	**HCT116 WT/OxR**	**relative resistance**^c^	**HCT116 p53KO**	**relative resistance**^c^
[Ag(PPh_**3**_)(bipy-**R**)]^+^					
**1**	2.72 ± 0.66	1.90 ± 0.54	0.7^n.s.^	2.63 ± 0.40	1.0^n.s.^
**2**	3.78 ± 0,30	3.33 ± 0.03	0.9^n.s.^	3.69 ± 0.07	1.0^n.s.^
**3**	2.96 ± 0.67	2.08 ± 0.50	0.7^n.s.^	2.60 ± 0.40	0.9^n.s.^
[Ag(dppe)(bipy-**R**)]^+^					
**4**	0.70 ± 0.07	0.65 ± 0.09	0.9^n.s.^	0.46 ± 0.20	0.7^n.s.^
**5**	0.74 ± 0.06	0.75 ± 0.08	1.0^n.s.^	0.43 ± 0.05	0.6^n.s.^
**6**	0.64 ± 0.06	0.54 ± 0.11	0.8^n.s.^	0.49 ± 0.14	0.8^n.s.^
**7**	0.35 ± 0.04	0.38 ± 0.05	1.1^n.s.^	0.34 ± 0.01	1.0^n.s.^
**oxaliplatin**	0.72 ± 0.18	>10	>14**	2.37 ± 0.45	3.3**

a ***p ≤ 0.001; **p ≤ 0.01; *p ≤ 0.05. n.s., not significantly different, calculated by one-sample *t* test.

b IC_50_ values were calculated from concentration–response curves. Values are given as mean ± SD of three independent experiments performed in triplicates.

c Differences in sensitivity calculated by dividing the IC_50_ values of the resistant subline by those of the parental line.

For further studies, the three complexes with unmodified bipyridine ligands (**1**, **4**) and compound **7** were selected ZX. To gather more information on the time dependency of the activity of the selected drugs, time course experiments as well as long-term clonogenic assays were performed. In SK-OV-3, all three drugs had already full activity after 24 h. In contrast, the exceptional sensitivity of MES-OV cells upon treatment with **4** and **7** was observed only at 72 h, whereas short-term incubation of 24 h resulted in even slightly lower activity as compared to compound **1** (Table S2). Noteworthily, after 24 h, **7** was about twofold more active than **4**, which could point toward a faster onset of activity with this drug. Interestingly, in the clonogenic assays after 10 days, the results in the MES-OV cells were rather similar to the 72 h MTT assays. In contrast, the dppe-bearing drugs exerted a more pronounced activity against SKOV-3, reaching IC_50_ values in the nM range (Figure S39).

#### Intracellular Accumulation of Compounds **1**, **4**, and **7** in OC Models

**Figure 6:**
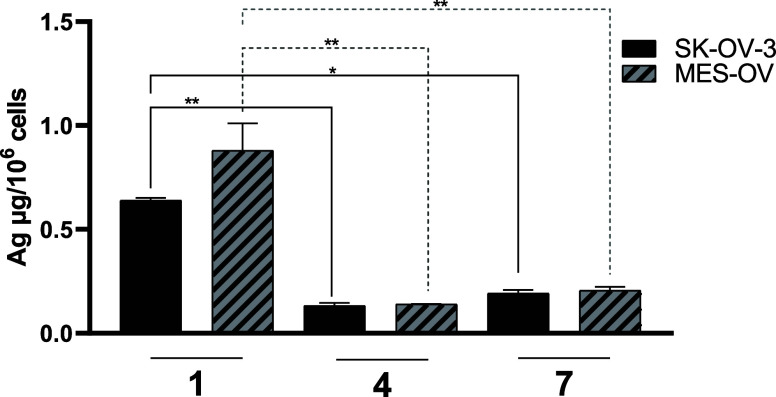
Intracellular silver levels after treatment with **1**, **4,** or **7** (10 μM) in SK-OV-3 and MES-OV after 5 h were determined by ICP-MS. Results were normalized to the cell number, and values are given as mean ± SD of two independent experiments. Statistical significance was tested by two-way ANOVA and Sidak′s multiple comparison test (**p < 0.01; *p < 0.05).

To get more insight into the differences in the cytotoxicity of the studied compounds, the intracellular levels of the drugs were investigated. Therefore, we evaluated the three chosen drugs (**1, 4**, and **7**) in SK-OV-3 and MES-OV cells after 5 h treatment with equimolar drug concentrations by ICP-MS (Figure 6). In general, the dppe-bearing compounds **4** and **7** were distinctly lower than **1** in their cellular accumulation in both OC cell models. Noteworthily and although **4** and **7** were more active, the intracellular silver levels for the PPh_3_-bearing compound **1** were 3 times higher. Moreover, despite their distinct differences in sensitivity, the drug uptake was comparable between SK-OV-3 and MES-OV cells. Thus, based on these results, we can conclude that the anticancer activity of the drugs is not explained by the intracellular silver levels.

#### Thioredoxin Reductase Inhibition Potential of the New Drugs

**Figure 7:**
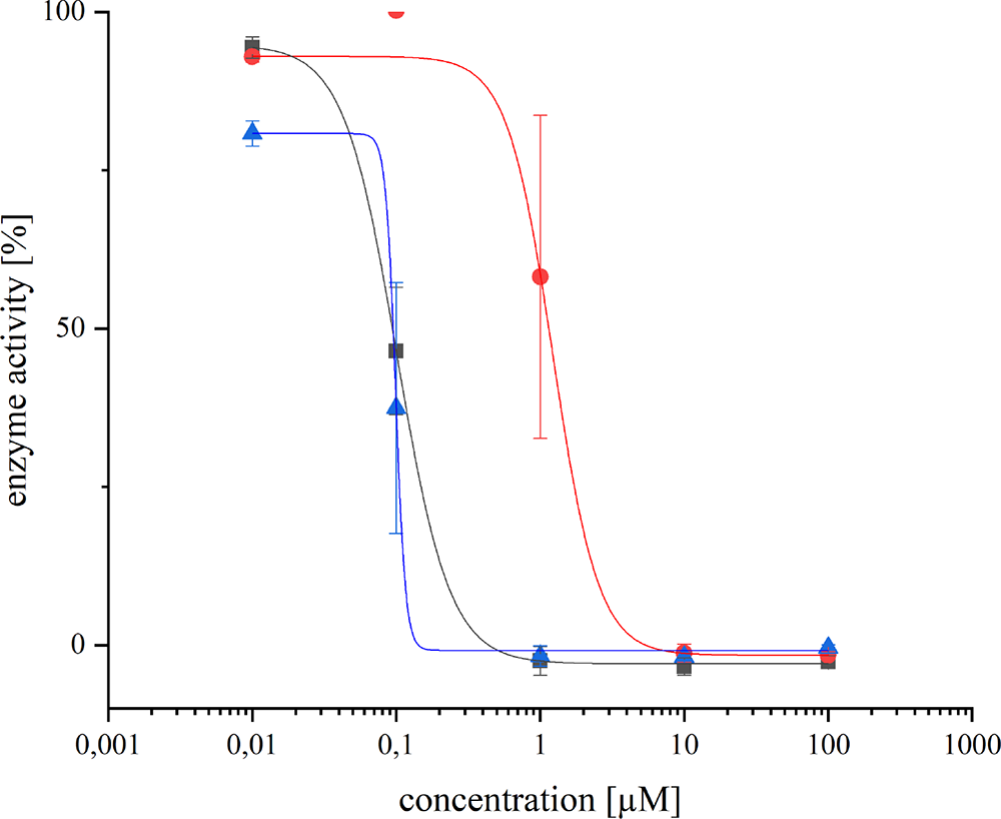
TrxR activity after incubation with different concentrations of the selected compounds (IC_50_ [μM] ± SD) **1** (▲): 0.093 ± 0.014; **4** (■): 0.101 ± 0.009; **7** (●): 1.021 ± 0.192.

One frequently discussed target for silver drugs is the enzyme thioredoxin reductase (TrxR).^7^ Different types of silver complexes have demonstrated strong activity against TrxR.^32,33^ Consequently, possible differences in TrxR inhibition properties were evaluated with an established microplate reader-based assay. Unexpectedly, in this cell-free assay, compounds **1** and **4** turned out to be 10-fold stronger TrxR inhibitors than **7** (Figure 7). This means that TrxR inhibition could contribute to the anticancer activity of these compounds, however, it does not explain the high sensitivity of the MES-OV cells to the dppe-bearing compounds.

#### Apoptotic Cell Death Is Involved in Silver Drug Activity only at Higher Drug Levels

To gain more information on the mode of action of the tested silver compounds, their impact on cell cycle distribution and their apoptosis-inducing potential were investigated after 24 h. With regard to the cell cycle distribution, drug treatment did not lead to significant changes with the exception of the highest concentration of **1** (10 μM). In these samples, a significant shift from the G0–G1 phase to the S phase was observed in both SK-OV-3 and MES-OV cells (Figure S40). Consequently, we concluded that cell cycle arrest and senescence are not the major drivers of the MES-OV-selective anticancer activity of the dppe-bearing compounds at lower drug concentrations.

**Figure 8:**
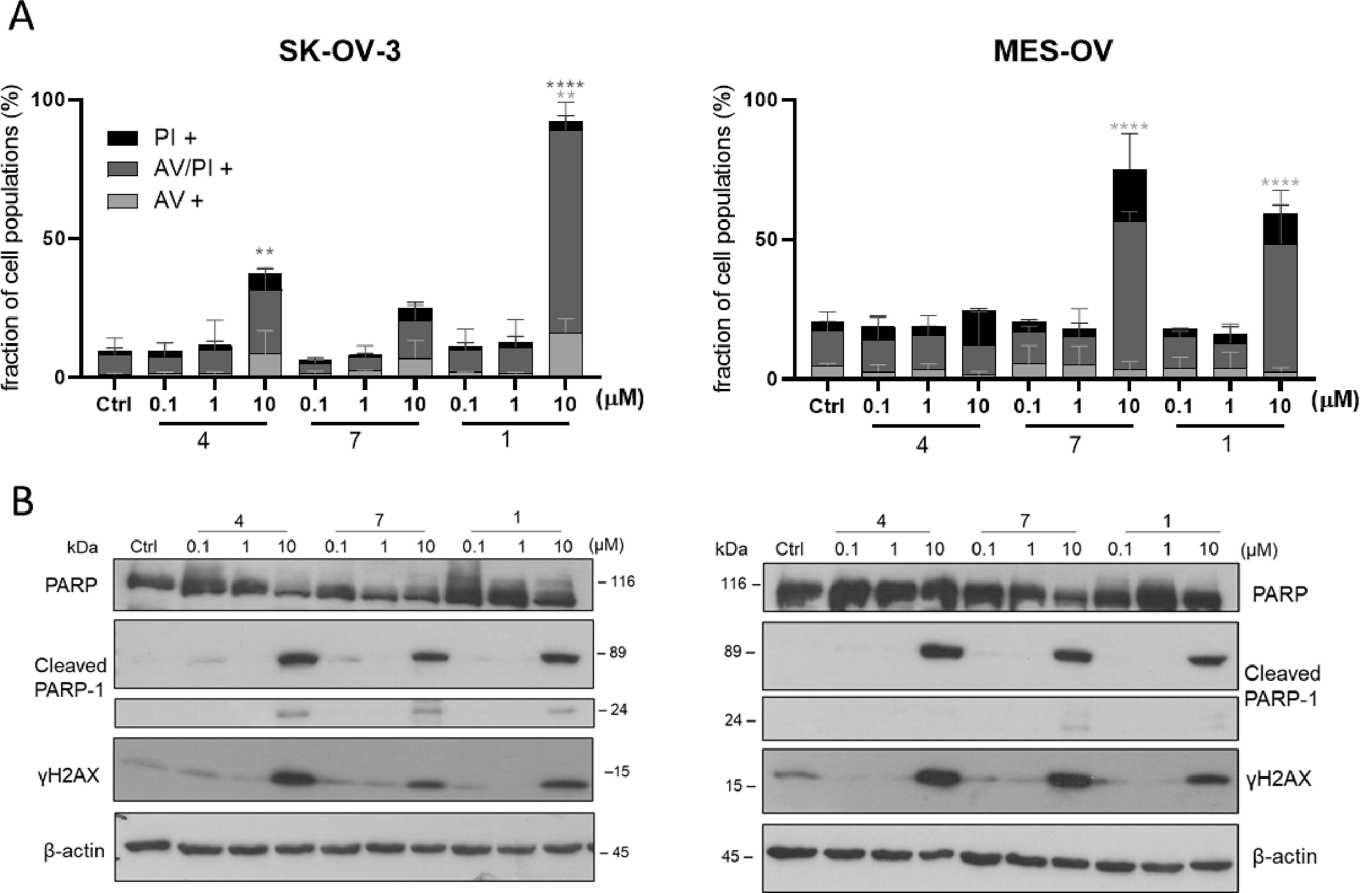
Apoptosis induction by the novel silver drugs in SK-OV-3 and MES-OV cells. (A) Cell death was determined by annexin V/propidium iodide (AV/PI) stain by flow cytometry after 24 h treatment with the indicated concentrations. Means ± SD were derived from three independent experiments. Significance to control was calculated by two-way ANOVA and Bonferroni’s multiple comparison test using the GraphPad Prism software (***p < 0.001, **p ≤ 0.01, *p ≤ 0.05). (B) Protein expression of PARP and cleaved PARP-1 as a late-phase apoptosis marker as well as γH2AX as a DNA damage marker was detected by Western blot after 24 h treatment with the tested silver compounds at the indicated concentrations.

Interestingly, also when looking for cell death induction by annexin V/propidium iodide (AV/PI) stains, strong apoptosis induction (up to 100% in case of SK-OV-3 and **1**) was detected only at 10 μM concentrations after 24 h (Figure 8A). This was also confirmed by Western blot analysis, where PARP cleavage, an indicator for late-phase apoptosis, was visibly increased only after treatment with the highest concentration of the silver compounds (Figure 8B). Moreover, to evaluate the role of DNA damage in the mode of action in the cell death induced by our silver compounds, DNA laddering (Figure S41) and γH2AX-dependent damage signaling (Figure 8B) were investigated. The γH2AX results indicated that the enhanced sensitivity of the MES-OV cells was not based on DNA damage, which is in good agreement with the observation that the cellular P53 status had no impact on the drug sensitivity of the cancer cells. In addition, our compound did not induce DNA fragmentation, which would be characteristic for apoptotic cell death, *e.g.*, induced by H_2_O_2_.^34,35^

Together with the other results, this clearly indicates that (DNA damage-associated) apoptotic cell death is not responsible for the enhanced activity of the dppe-bearing drugs against MES-OV cells.

#### Dppe-Bearing Compounds Induce Paraptotic Cell Death in MES-OV Cells

**Figure 9:**
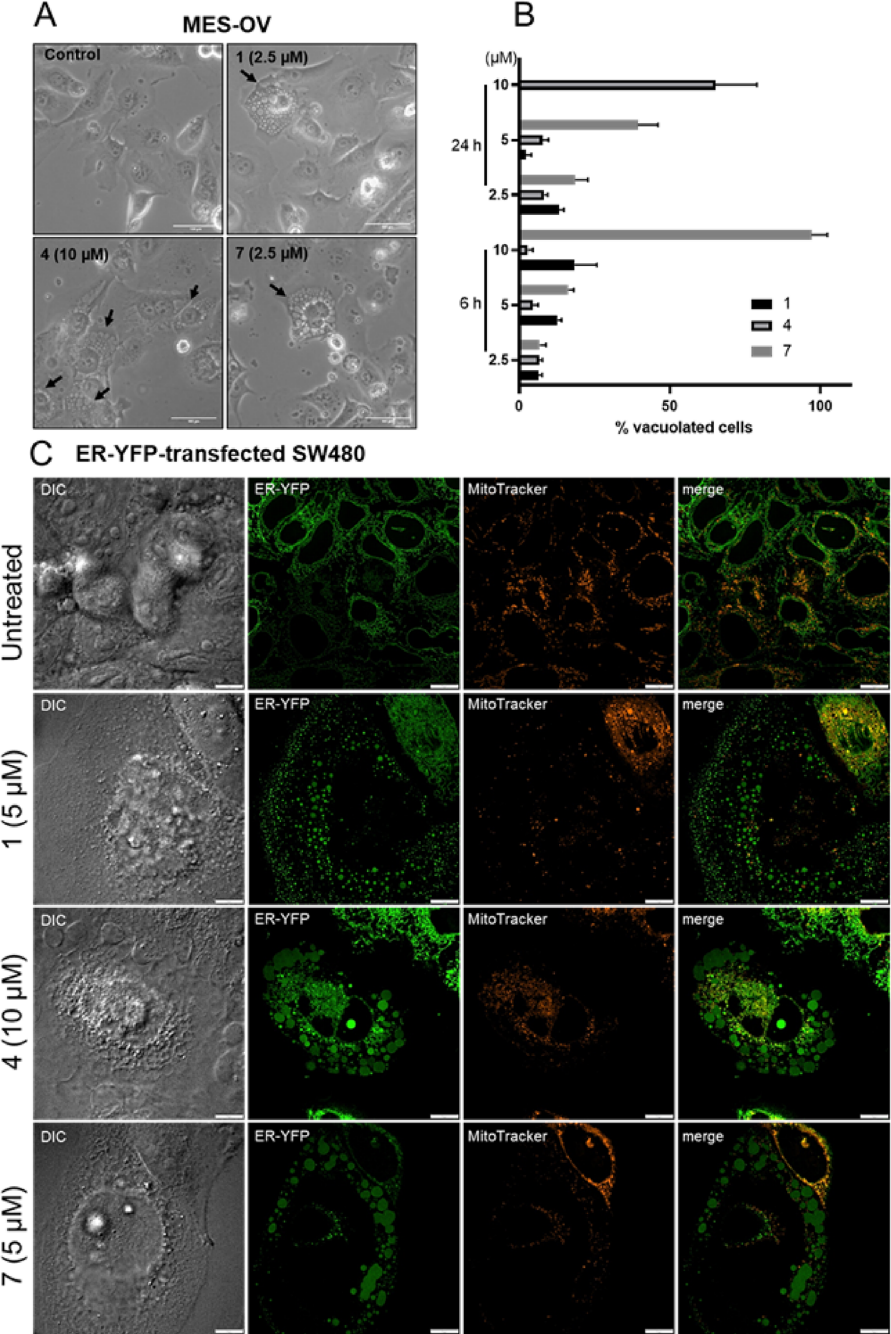
Cytoplasmatic vacuoles indicative for paraptotic cell death induced by silver drug treatment. (A) Phase contrast images of MES-OV cells treated with the indicated drugs for 24 h (20× magnification, scale bar: 100 μm). (B) Percentage of vacuolated cells counted from phase-contrast microscopy images seen in panel A and Figure S42 after 6 and 24 h treatment. Values given are the mean ± standard deviation of three images with at least 30 cells in total. (C) Spinning disk confocal microscopy of ER-YFP-transfected SW480 cells treated with the indicated drugs and concentrations for 24 h. Representative pictures were taken in confocal mode, *Z*-stack, and max intensity projection (192× magnification and 60× objectives) of vesicles in ER-YFP-transfected SW480 (ER-YFP in green), DIC (differential interference contrast), and mitochondria (MitoTracker in red).

When looking for differences between the reaction of SK-OV-3 and MES-OV to the new drugs, we observed that the MES-OV cells were characterized by distinct morphological changes, especially upon treatment with the dppe-bearing compounds (Figure 9A,B as well as Figure S42). In detail, already after 6 h treatment with 2.5 μM of **7**, the formation of cytoplasmic vacuoles near the nucleus within the cytoplasm was observed (highlighted by the black arrows in Figure 9A). Also, **4** induced pronounced cytoplasmic vacuoles only after 24 h incubation, suggesting again that the activity of **7** might be faster than **4**. In contrast, **1**-treated cells exhibited distinctly fewer vesicles of smaller and distinctive shapes.

Strong cytoplasmic vacuolization could be an indicator for paraptosis, a recently described form of programmed cell death^36,37^ where the endoplasmatic reticulum (ER) forms large vesicles together with mitochondrial fragmentation/swelling and dysfunction. To investigate the origin of the vesicles in more detail, spinning-disk confocal microscopy of cells stained with ER Tracker Red (stains ER membrane) and Mito Tracker Green (stains mitochondrial mass) was performed (Figure S43). The ER depicts a reticulate structure in the untreated cells, whereas treatment with the silver compounds induced large vacuoles of which membranes were stained with the ER tracker. In parallel, mitochondrial fragmentation/swelling and an overlap of mitochondria with ER vesicle membranes were found. In addition, the mitochondrial membrane potential (low Δψ fraction) was investigated by JC-1 stain (Figure S44). After 24 h, treated MES-OV cells displayed 30 and 90% of cells with mitochondrial depolarization after 1 and 10 μM **7** treatment, respectively. In contrast, **1** and **4** only showed an impact with the highest concentration of 10 μM, where 50% of the cells displayed mitochondrial depolarization. The ER-derived nature of the vesicles and mitochondrial fragmentation/swelling were confirmed using a SW480 subclone, which was transfected with an ER-tracked YFP (resulting in a luminal stain of the ER, Figure 9C).^38^

**Figure 10:**
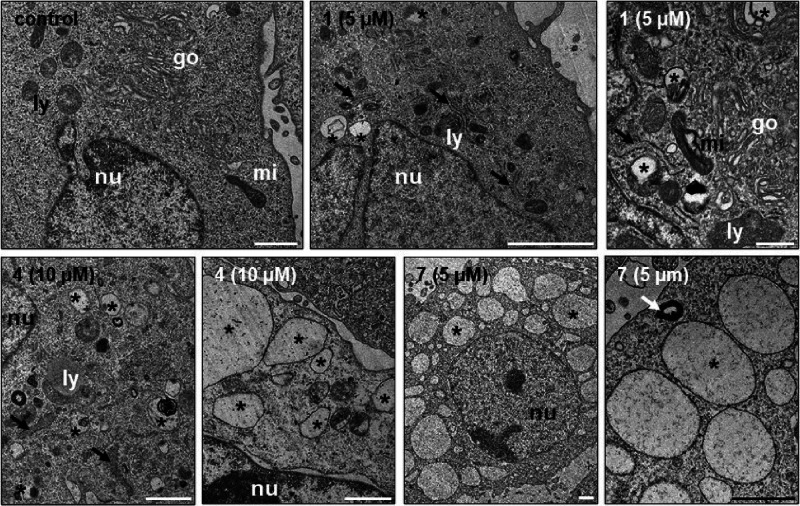
TEM analyses of MES-OV cells after 24 h treatment with the indicated drugs. Control cells exhibited a normal subcellular organization. In **1**-treated samples small, single-membrane vacuoles (asterisks) and slightly thicker rough ER (black arrows) are indicated. Frequently, lysosomes and mitochondria exhibit degradation (asterisks). Upon therapy with **4**, swollen rough ER (black arrows) and lysosomes with irregular lamellar structures were present (asterisk). Other cells exhibited stronger vacuolization (asterisks). In **7**-treated samples, the cytoplasm was mostly devoid of organelles and filled with vacuoles (asterisks). Frequently, multilamellar bodies (white arrow) were found. Abbreviations: go = Golgi, ly = lysosome, mi = mitochondrium, nu = nucleus, black arrow = rough endoplasmatic reticulum, white arrow = multilamellar body, asterisk = single-membrane vacuole. All scalebars = 1 μm.

To gain more insights into subcellular events, we performed transmission electron microscopy (TEM) analyses after 24 h drug treatment (Figure 10). In contrast to the control that exhibits a normal subcellular organization (mitochondria, Golgi, lots of smooth ER and few rough ER, some lysosomes), **1** induced small, single-membraned vacuoles (300–500 nm in diameter) near the nucleus, slightly thicker rough ER, as well as a higher number of lysosomes that exhibit degradation (likewise detectable in the few mitochondria). In **4**-treated samples, the rough ER was distinctly swollen, and lysosomal degradation additionally was evident by irregular lamellar structures. Other cells exhibited stronger vacuolization (300–1500 nm in diameter), and mitochondria were completely absent. The amount of smooth and rough ER was distinctly smaller than in control and **1**-treated cells. Upon treatment with **7**, the cytoplasm was mostly devoid of organelles and generally filled with small (300 nm) to big vacuoles (up to 7.5 μm). Frequently, multilamellar bodies were present.

**Figure 11:**
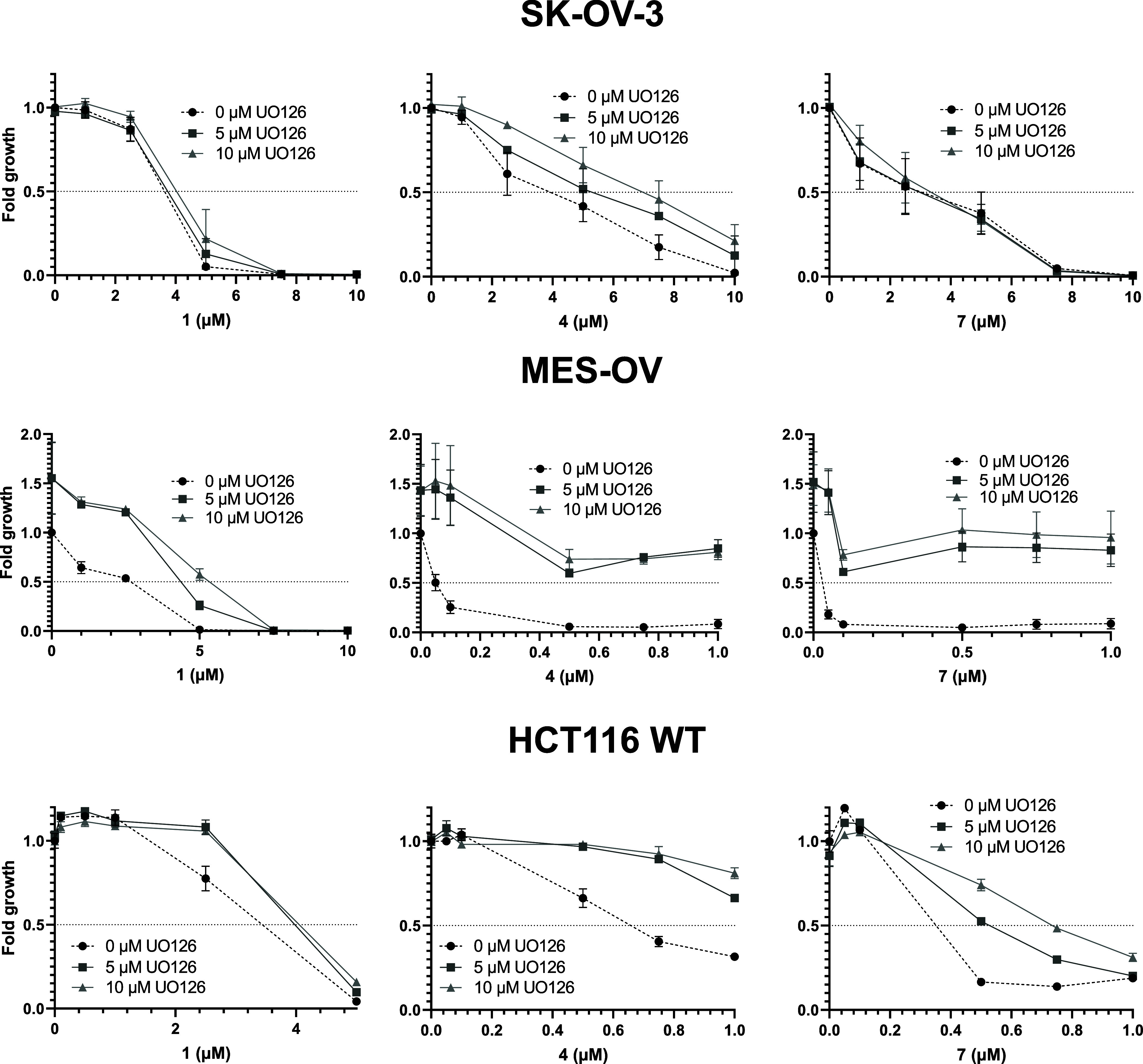
Impact of the MAPK inhibitor U0126 (5 and 10 μM) on the anticancer activity of the tested silver compounds. To evaluate the cells' viability, an MTT assay was performed after 72 h of combined drug treatment. The mean ± SD was derived from triplicates of one representative experiment out of three.

Paraptosis is a programmed form of cell death independent of caspase signaling.^39^ Accordingly, also in the case of the silver drugs, the addition of the pan-caspase inhibitor z-VAD-FMK had no impact on the ER-derived vacuoles (Figure S45) or anticancer activity (Figure S46). Another hallmark of paraptosis is the dependence on the mitogen-activated protein kinase (MAPK) signaling.^40,41^ Comparable to other paraptosis inducers, also in the case of this drug panel, the MAPK inhibitor U0126 had protective effects (Figure 11). Noteworthily, the effect was stronger in MES-OV cells for **4** and **7** followed by **1**. A similar picture was seen for HCT116 cells but was less pronounced, whereas in SK-OV-3 cells, only in case of **4** a protective effect by U0126 cotreatment was observed. This is in good agreement with the individual IC_50_ values indicated in Tables 1 and 2. Together, these results suggest that the enhanced sensitivity of some cancer cell types against dppe-bearing compounds is associated with paraptosis induction.

#### The Role of ER Stress and Altered Redox Homeostasis in the Dppe-Silver Drug-Induced Paraptosis of MES-OV Cells

**Figure 12:**
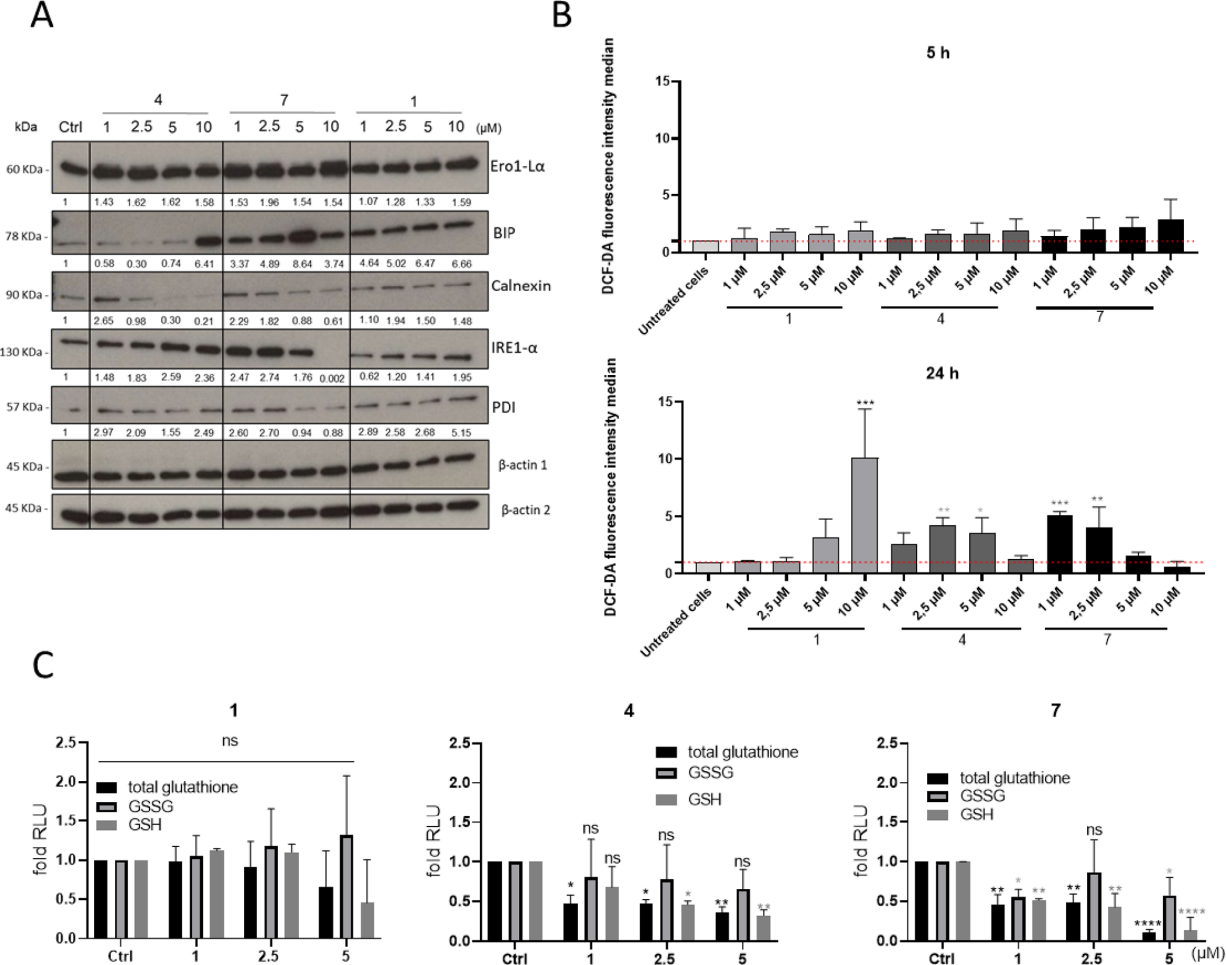
(A) Protein expression of ER-stress markers was detected by Western blot after 24 h treatment with the tested silver compounds at the indicated concentrations. (B) ROS production was measured by flow cytometry of DCF-DA fluorescence in MES-OV cells treated with indicated concentrations of the tested silver complexes after 5 and 24 h. Significance to control was calculated with one-way ANOVA and Dunnett’s multiple comparison test (****p < 0.0001, ***p < 0.0002, **p ≤ 0.002, *p ≤ 0.03, ns 0.12). (C) Changes in total, reduced and oxidized glutathione (GSH vs GSSG) are shown by fold increase to control of luminescence in MES-OV cells treated with the indicated drugs for 24 h. Significance to control was calculated with two-way ANOVA and Tukey’s multiple comparison test (****p < 0.0001, ***p < 0.0002, **p ≤ 0.002, *p ≤ 0.03, ns 0.12).

Paraptosis is a quite recently discovered form of cell death. Consequently, the exact causes and molecular targets are still not fully understood. Frequently, ER stress has been reported, which however often differs from classical ER stress inducers, e.g., thapsigargin.^42,43^ Also, in case of the investigated silver compounds, some ER stress markers were activated in MES-OV cells (Figure 12A). In detail, ER stress indicators including Ero1-Lα, BiP (GRP78), and IRE1-α were upregulated upon treatment with all tested drugs. However, only in case of **1** this upregulation followed a dose–response pattern, whereas in case of the others, a plateau was reached. Interestingly, with the exception of the ER stress chaperons BiP and calnexin, the regulation patterns did not follow the strength of the observed paraptotic morphology (compare Figure 9B). In more detail, the strongest BiP signals were found in paraptotic cells, whereas calnexin was down-regulated in a dose-dependent manner only with **4** and **7** but not upon **1** treatment. Thus, although we observed ER stress with the paraptosis-inducing silver compounds, it is difficult to conclude whether it is a cause or just a consequence of the paraptotic cell death.

Several studies (*e.g.*, tocotrienol) have demonstrated that some forms of paraptosis are associated with elevated oxidative stress.^36,44^ Consequently, ROS levels after treatment with the silver drugs were measured by DCF-DA assay. Whereas no significant increase was detected in MES-OV cells after 5 h of drug treatment, an interesting pattern was seen after 24 h (Figure 12B). Thus, in case of **1**, a significant and very strong elevation in ROS was found only in the 10 μM sample. In contrast, upon **4** and **7** treatment, ROS levels increased in the lower drug levels only (with a peak of ∼5-fold at 2.5 μM). Noteworthily, no oxidative stress was seen in SK-OV-3 cells at the same experimental conditions (Figure S47). Interestingly, subsequent investigation of the reduced and oxidized glutathione levels (GSH vs GSSG) revealed that the ROS were not accompanied by increased levels of GSSG but in contrast by a general reduction of total glutathione (Figure 12C). The strength of this effect followed the same trend as the paraptosis induction, with the strongest reduction for **7** and the weakest for complex **1**. Consequently, this change in redox balance seems to be strongly connected with the paraptosis induction, which will be investigated in more depth in future studies.

## 3 Conclusions

In this paper, we described the synthesis and biological evaluation of the first Ag(I)-bipyridine compounds bearing phosphane ligands as promising anticancer agents. The new silver(I) conjugates of general formulas [Ag(bipyR)(phosphane)][CF_3_SO_3_] and [Ag(dppe)_2_][CF_3_SO_3_] were synthesized and characterized in solution and in the solid state by means of several spectroscopic techniques, in good agreement with the proposed structures. Additionally, single-crystal X-ray diffraction studies were performed for compounds **1**–**4**. We chose to develop this family of compounds because, as far as we are aware, these structures have never been evaluated for their anticancer potential and surprisingly are virtually unexplored. Only a report on the synthesis of silver(I) complexes with various phenanthroline ligands and different bis-phosphane derivatives has been found.^45^ Regarding the use of monodentate phosphanes, such as PPh_3_, together with *N*,*N*′-bidentate-type ligands, only the synthesis of a family of [Ag(PPh_3_)(N,N’)]^+^(NO_3_^–^) compounds (*N*,*N*’ = 2,2′-bypiridine, 1,10-phenanthroline, 2,9-dimethyl-1,10-phenanthroline, 2,20-biquinolyl, bis(2-pyridyl)amine) has been reported.^46^ In our study, we also included the compound [Ag(dppe)_2_][CF_3_SO_3_], for which an old report on the cation indicated its potential as an anticancer agent.^47^ Globally, our structure–activity studies revealed that these complexes potently overcome drug resistance in platinum-resistant cancer cells. Remarkably, the introduction of the dppe moiety leads to specific targeting of an OC subtype resulting in anticancer activity in the low nM range. Subsequent studies on three selected compounds indicated that these effects are not based on enhanced drug uptake, stronger TrxR inhibition properties, cell cycle arrest, or pronounced apoptosis induction. In contrast, we found that especially silver compounds with a dppe moiety induce paraptosis, a recently described form of programmed cell death. Together, these make our new class of silver compounds interesting candidates for further preclinical development against drug-resistant OC.

## 4 Experimental Section

### 4.1 Materials

All chemicals were purchased from commercial sources and used without further purification (unless otherwise stated).

### 4.2 Instrumentation and Methods (Experimental Section)

#### General Procedures

All reactions and purification of compounds were performed under a nitrogen atmosphere using Schlenk techniques. All solvents were used as purchased. Dichloromethane and *n*-hexane used for synthetic procedures and workup were dried using an MBRAUN solvent purification system (MB SPS-800, M Braun Inertgas-Systeme GmbH, Garching, Germany). NMR spectra were recorded on a Bruker Avance 400 spectrometer at probe temperature using commercially available deuterated dimethyl sulfoxide. Chemical shifts (δ) are reported in parts per million (ppm) referenced to tetramethylsilane (δ 0.00 ppm) using the residual proton solvent peaks as internal standards. ^31^P{^1^H} NMR chemical shifts were reported downfield from external standard 85% H_3_PO_4_. The multiplicity of the peaks is abbreviated as follows: s (singlet), d (doublet), t (triplet), m (multiplet), comp (complex), br (broad). Coupling constants (*J*) are reported in hertz (Hz). All assignments were attributed using COSY, HMBC, and HMQC 2D-NMR techniques. Infrared spectra were recorded on KBr pellets using a Mattson Satellite FT-IR spectrophotometer. Only considered relevant bands were cited in the text. Electronic spectra were recorded at room temperature on a Jasco V-660 spectrometer from solutions of 10^–3^–10^–6^ M in quartz cuvettes (1 cm optical path). LC-ESI(+) analysis was performed on an Elute UPLC system (Bruker, Bremen, Germany) connected with a Bruker Impact II quadrupole time-of-flight mass spectrometer equipped with an ESI source (Bruker Daltoniks, Bremen, Germany). For the chromatographic separations, a Luna C18 (2) column (150 × 2.0 mm inner diameter; 3.0 μm particle size, Phenomenex) equipped with a C18 (Phenomenex) guard disk (2 × 4 mm) was used. The mobile phase consisted of water (mobile phase A) and acetonitrile (mobile phase B) at a flow rate of 170 μL/min. A 20 min gradient was used as follows: 70–100% B for 9 min; isocratic elution with 100% B for 7 min; 100–70% B for 1 min; and finally, 70% B for 3 min. All compounds are >95% pure by elemental analysis. Elemental analyses were performed at *Laboratório de Análises* at *Instituto Superior Técnico* using a Fisons Instruments EA1 108 system.

### 4.3 Synthesis of the Silver Compounds

#### Synthesis of the Compounds [Ag(bipyR)(PPh_3_)][CF_3_SO_3_] (**1**–**3**)

Compounds of general formula [Ag(bipyR)(PPh_3_)][CF_3_SO_3_] (**1**–**3**) were prepared by mixing PPh_3_ (103 mg, 0.4 mmol) to a rapidly stirred solution of AgCF_3_SO_3_ (100 mg, 0.4 mmol) in 10 mL of dichloromethane followed immediately by the addition of 2,2′-bipyridine (62 mg, 0.4 mmol, for **1**), 4,4′-dimethyl-2,2′-bipyridine (75 mg, 0.4 mmol, for **2**), or 4,4′-dihydroxymethyl-2,2′-bipyridine (86 mg, 0.4 mmol, for **3**). The mixture was stirred and protected from light at room temperature for 3 h. Removal of the solvent in vacuum left a white to a slightly pale-yellow residue. The residue was washed three times with *n*-hexane or diethyl ether (3 × 5 mL) until the residue was transformed into a white powder. Colorless crystals suitable for X-ray crystallographic analysis were obtained by slow evaporation of a solution of acetonitrile (**1**) or by slow diffusion, at room temperature, of *n*-hexane into dichloromethane (**2**) or slow diffusion of diethyl ether into acetone (**3**).

##### [Ag(2,2′-bipy)(PPh_3_)][CF_3_SO_3_] (**1**)

Yield: 98% (267 mg). ^1^H NMR [(CD_3_)_2_SO, Me_4_Si] δ(ppm): 8.78 (d, 2H, ^3^*J*_HH_ = 4, H6), 8.55 (d, 2H, ^3^*J*_HH_ = 8, H3), 8.17 (t, 2H, ^3^*J*_HH_ = 8, H4), 7.68 t, 2H, ^3^*J*_HH_ = 8, H5), 7.53 (comp, 15H, H_*ortho*_ + H_*meta*_ + H_*para*_). APT-^13^C{^1^H} NMR [(CD_3_)_2_SO] δ(ppm): 152.3 (C2), 150.8 (C6), 139.4 (C4), 135.6 (d, ^2^*J*_CP_ = 17, C_*ortho*_), 131.3 (d, ^1^*J*_CP_= 34, C_*q*_PPh_3_), 131.1 (br, C_*para*_PPh_3_), 129.4 (d, ^3^*J*_CP_ = 10, C_*meta*_), 125.8 (C5), 122.6 (C3). ^31^P{^1^H} NMR [(CD_3_)_2_SO, 293 K] δ(ppm): 13.52 (d br, ^1^*J*_AgP_ = 578, PPh_3_). ^31^P{^1^H} NMR [CDCl_3_, 233 K], δ(ppm): 17.4 (2 d, ^1^*J*(^109^Ag–^31^P) = 740, ^1^*J*(^107^Ag–^31^P) = 643, PPh_3_). FTIR [KBr, cm^–1^]: 3084–3005 (υ_C–H_ aromatic rings), 1435 (υ_C=C_ aromatic rings), 1273 (υCF_3_SO_3_ counterion). UV–vis [DMSO, λ_max_/nm (ε × 10^3^/M^–1^cm^–1^)]: 277 (18.63), 298 (sh). Elemental analysis calc. for C_29_H_23_F_3_N_2_O_3_PAgS (675.41 g mol^–1^): C, 51.57; H, 3.43; N, 4.15; S, 4.75. Found: C, 51.4; H, 3.3; N, 4.1; S, 5.0. ESI-MS(+): [**1**-CF_3_SO_3_]^+^ calc. for [C_28_H_23_N_2_PAg]^+^: 525.06. Found: 525.12 (100%).

##### [Ag(4,4′-CH_3_-2,2′-bipy)(PPh_3_)][CF_3_SO_3_] (**2**)

Yield: 96% (270 mg). ^1^H NMR [(CD_3_)_2_SO, Me_4_Si] δ(ppm): 8.62 (d, 2H, ^3^*J*_HH_ = 8, H6), 8.43 (s, 2H, H3), 7.53 (comp, 17H, H_*ortho*_ + H_*meta*_ + H_*para*_ + H5), 3.37 (s, 6H, CH_3_). APT-^13^C{^1^H} NMR [(CD_3_)_2_SO] δ(ppm): 152.5 (C2), 151.2 (C4), 150.8 (C6), 134.0 (d, ^2^*J*_CP_ = 17, C_*ortho*_), 131.8 (d, ^1^*J*_CP_= 34, C_*ipso*_), 131.5 (br, C_*para*_), 129.9 (d, ^3^*J*_CP_ = 10, C_*meta*_), 126.9 (C5), 123.7 (C3), 21.3 (C7). ^31^P{^1^H} NMR [(CD_3_)_2_SO, 293 K], δ(ppm): 13.64 (d br, ^1^*J*_AgP_ = 534, PPh_3_). ^31^P{^1^H} NMR [CDCl_3_, 233 K], δ(ppm): 17.8 (2 d, ^1^*J*(^109^Ag–^31^P) = 739, ^1^*J*(^107^Ag–^31^P) = 640, PPh_3_). FTIR [KBr, cm^–1^]: 3068 (υ_C–H_ aromatic rings), 1436 (υ_C=C_ aromatic rings), 1276 (υCF_3_SO_3_ counterion). UV–vis [DMSO, λ_max_/nm (ε × 10^3^/M^–1^cm^–1^)]: 277 (22.25), 299 (sh). Elemental analysis calc. for C_31_H_27_F_3_N_2_O_3_PAgS (703.46 g mol^–1^): C, 52.93; H, 3.87; N, 3.98; S, 4.56. Found: C, 53.1; H, 3.9; N, 3.9; S, 5.0. ESI-MS(+): [**2**-CF_3_SO_3_]^+^ calc. for [C_30_H_27_N_2_PAg]^+^: 553.10. Found: 553.19 (100%).

##### [Ag(4,4′-CH_2_OH-2,2′-bipy)(PPh_3_)][CF_3_SO_3_] (**3**)

Yield: 95% (278 mg). ^1^H NMR [(CD_3_)_2_SO, Me_4_Si] δ(ppm): 8.70 (d, 2H, ^3^*J*_HH_ = 8, H6), 8.45 (s, 2H, H3), 7.60 (d, 2H, ^3^*J*_HH_ = 8, H5), 7.53 (m, 15H, H_*ortho*_ + H_*meta*_ + H_*para*_), 5.69 (t, 2H, ^3^*J*_HH_ = 4, −OH), 4.73 (d, 4H, ^3^*J*_HH_ = 4, H7). APT-^13^C{^1^H} NMR [(CD_3_)_2_SO] δ(ppm): 155.3 (C2), 152.2 (C4), 150.4 (C6), 133.6 (d, ^2^*J*_CP_ = 17, C_*ortho*_), 131.3 (d, ^1^*J*_CP_ = 34, C_*ipso*_), 131.1 (br, C_*para*_), 129.0 (d, ^3^*J*_CP_ = 10, C_*meta*_), 122.8 (C5), 119.3 (C3), 61.5 (C7). ^31^P{^1^H} NMR [(CD_3_)_2_SO, 293 K] δ(ppm): 13.50 (d br, ^1^*J*_AgP_ = 629, PPh_3_). ^31^P{^1^H} NMR [CDCl_3_, 233 K], δ(ppm): 17.6 (2 d, ^1^*J*(^109^Ag–^31^P) = 739, ^1^*J*(^107^Ag–^31^P) = 640, PPh_3_), 11.2 (d, ^1^*J*(^109^Ag–^31^P) = 557, ^1^*J*(^107^Ag–^31^P) = 483, PPh_3_ of [Ag(PPh_3_)_2_]^+^). FTIR [KBr, cm^–1^]: 3367 (υ_O–H_), 3053 (υ_C–H_ aromatic rings), 1435 (υ_C=C_ aromatic rings), 1274 (υCF_3_SO_3_ counterion). UV–vis [DMSO, λ_max_/nm (ε × 10^3^/M^–1^cm^–1^)]: 280 (20.72), 300 (sh). Elemental analysis calc. for C_31_H_27_F_3_N_2_O_5_PAgS (735.46 g mol^–1^): C, 50.63; H, 3.70; N, 3.81; S, 4.36. Found C, 50.7; H, 3.6; N, 3.7; S, 4.0. ESI-MS: [**3**-CF_3_SO_3_]^+^ calc. for [C_30_H_27_N_2_O_2_PAg]^+^: 585.09. Found: 585.03 (100%).

#### Synthesis of the Compounds [Ag(bipyR)(dppe)][CF_3_SO_3_] (**4**–**6**)

Compounds of general formula [Ag(bipyR)(dppe)][CF_3_SO_3_] (**4**–**6**) were prepared by mixing dppe (155 mg, 0.4 mmol) to a rapidly stirred solution of AgCF_3_SO_3_ (100 mg, 0.4 mmol) in 10 mL of dichloromethane followed immediately by the addition of 2,2′-bipyridine (62 mg, 0.4 mmol, **4**), 4,4′-dimethyl-2,2′-bipyridine (75 mg, 0.4 mmol, **5**), or 4,4′-dihydroxymethyl-2,2′-bipyridine (86 mg, 0.4 mmol, **6**). The mixture was stirred and protected from light at room temperature for 4 h. Removal of the solvent in vacuum left a white residue. The residue was washed three times with *n*-hexane or diethyl ether (3 × 15 mL) until the residue was transformed into a white powder. Colorless crystals suitable for X-ray crystallographic analysis were obtained by slow diffusion of *n*-hexane or diethyl ether into acetone at room temperature.

##### [Ag(2,2′-bipy)(dppe)][CF_3_SO_3_] (**4**)

Yield: 88%. (286 mg) ^1^H NMR [(CD_3_)_2_SO, Me_4_Si] δ/ppm: 8.71 (d, 2H, H6), 8.48 (d, ^3^*J*_HH_ = 8.07, 2H, H4), 8.08 (t, ^3^*J*_HH_ = 7.69, 2H, H5), 7.56 (d, 2H, H3), 7.65–7.12 (comp, 20H, H_*ortho*_ + H_*meta*_ + H_*para*_), 2.56 (comp, 4H, CH_2_–dppe). APT-^13^C{^1^H} NMR [(CD_3_)_2_SO, Me_4_Si] δ/ppm: 153.0 (C2), 150.2 (C6), 138.6 (C5), 125.2 (C3), 121.8 (C4), 132.5 + 131.08 + 129.2 (comp, C_*ortho*_ + C_*meta*_ + C_*para*_), 39.9 (CH_2_–dppe). ^31^P{^1^H} NMR [(CD_3_)_2_SO, 293 K] δ/ppm: 3.49 (2 d, ^1^*J*(^109^Ag–^31^P_dppe_) = 265.7, ^1^*J*(^107^Ag–^31^P_dppe_) = 230.0). FTIR [KBr cm^–1^]: 3057 υ(C–H aromatics), 2900 υ(C–H aliphatics), 1435 υ(CH_2_ aliphatics), 1259, 1153, 1028 υ(CF_3_SO_3_^–^). UV–vis [DMSO, λ_max_/nm (ε × 10^3^/M^–1^cm^–1^)]: 279 (21.76). Elemental analysis calc. for C_37_H_32_AgF_3_N_2_O_3_P_2_S. C 54.76; H 3.97; N 3.45; S 3.95; Found: C 54.85; H 3.82; N 3.29; S 3.33. ESI-MS (+, *m*/*z*) calc for [C_36_H_32_AgN_2_P_2_]^+^: 661.11. Found: 660.91.

##### [Ag(4,4′-CH_3_-2,2′-bipy)(dppe)][CF_3_SO_3_] (**5**)

Yield: 82%. (275 mg) ^1^H NMR [(CD_3_)_2_SO, Me_4_Si] δ/ppm: 8.51 (d, 2H, ^3^*J*_HH_ = 5.07, H6), 8.32 (s, 2H, H3), 7.36 (d, 2, ^3^*J*_HH_ = 5.32, H5), 7.65–7.12 (comp, 20H, H_*ortho*_ + H_*meta*_ + H_*para*_-dppe), 7.67 (d, 2H, ^3^*J*_HH_ = 7.67, H3), 2.56 (comp, 4H, CH_2_–dppe), 2.46 (s, 6H, H7). APT-^13^C{^1^H} NMR [(CD_3_)_2_SO, Me_4_Si] δ/ppm: 153.0 (C2), 149.8 (C6), 149.8 (C4), 132.5 + 129.2 + 125.4 (comp, C_*ortho*_ + C_*meta*_ + C_*para*_), 125.9 (C5), 122.6 (C3), 39.9 (CH_2_*-*dppe), 20.8 (C7). ^31^P{^1^H} NMR [(CD_3_)_2_SO, 293 K] δ/ppm: 3.50 (2 d, ^1^*J*^*109*^_AgP-dppe_ = 265.7, ^1^*J*^*107*^_AgP-dppe_ = 233.3). ^31^P{^1^H} NMR [CDCl_3_, 233 K] δ/ppm: 13.19 (m). FTIR [KBr cm^–1^]: 3057 υ(C–H aromatics), 2900 υ(C–H aliphatics), 1608 υ(C=C aromatics), 1435 υ(CH_2_ aliphatics), 1259, 1153, 1028 υ(CF_3_SO_3_^–^). UV–vis [DMSO, λ_max_/nm (ε × 10^3^/M^–1^cm^–1^)]: 281 (20.68). Elemental analysis calc. for C_39_H_36_AgF_3_N_2_O_3_P_2_S. C 55.79; H 4.32; N 3.34; S 3.82. Found: C 55.79; H 4.15; N 3.33; S 3.46. ESI-MS (+, *m*/*z*) calc for [C_38_H_36_AgN_2_P_2_]^+^: 689.14. Found: 688.91.

##### [Ag(4,4′-CH_2_OH-2,2′-bipy)(dppe)][CF_3_SO_3_] (**6**)

Yield: 85%. (296 mg) ^1^H NMR [(CD_3_)_2_SO, Me_4_Si] δ/ppm: 8.59 (d, 2H, ^*3*^*J*_HH_ = 5.09, H6), 8.40 (s, 2H, H3), 7.47 (d, 2H, ^*3*^*J*_HH_ = 5.30, H5), 5.65 (t, 2H, *J*_HH_ = 5.76, −OH), 7.60–7.25 (comp, 20H, H_*ortho*_ + H_*meta*_ + H_*para*_), 4.69 (d, 4H, *J*_HH_ = 5.44, H7), 2.50 (comp, 4H, *CH*_*2*_-dppe). APT-^13^C{^1^H} NMR [(CD_3_)_2_SO, Me_4_Si] δ/ppm: 154.5 (C4), 153.1 (C3), 149.9 (C6), 132.6 + 130.55 + 129.2 (comp, C_*ortho*_ + C_*meta*_ + C_*para*_), 122.2 (C5), 118.7 (C3), 61.5 (C7), 39.8 (*CH*_*2*_*–*dppe). ^31^P{^1^H} NMR [(CD_3_)_2_SO, 293 K] δ/ppm: 3.52 (2 d, ^1^*J*(^109^Ag–^31^P_dppe_) = 267.3, ^1^*J*(^107^Ag–^31^P_dppe_) = 231.6). FTIR [KBr cm^–1^]: 3446 υ(O–H), 3057 υ(C–H aromatics), 2900 υ(C–H aliphatics), 1604 υ(C=C aromatics), 1435 υ(CH_2_ aliphatics), 1259, 1153, 1028 υ(CF_3_SO_3_^–^). UV–vis [DMSO, λ_max_/nm (ε × 10^3^/M^–1^cm^–1^)]: 280 (26.76). Elemental analysis calc. for C_39_H_36_AgF_3_N_2_O_5_P_2_S. C 53.74; H 4.16; N 3.21; S 3.67. Found: C 53.71; H 4.18; N 3.06; S 3.53. ESI-MS (+, *m*/*z*) calc for [C_38_H_36_AgN_2_P_2_]^+^: 721.13. Found: 720.88.

#### Synthesis of the Compound [Ag(dppe)_2_][CF_3_SO_3_] (**7**)

Compound **7** was prepared by mixing dppe (310 mg, 0.8 mmol) to a rapidly stirred solution of AgCF_3_SO_3_ (100 mg, 0.4 mmol) in 10 mL of dichloromethane. The mixture was stirred and protected from light at room temperature for 2 h. The product was dried under a vacuum and washed three times with *n*-hexane or diethyl ether (3 × 15 mL) until the residue was transformed into a white powder.

Yield: 91%. (379 mg) ^1^H NMR [(CD_3_)_2_SO, Me_4_Si, δ/ppm]: 7.53 (t, 8H, H_*meta*_), 7.40–7.35 (m, 8H, H_*ortho*_), 7.38 (t, 4H, H_*para*_), 2.56 (comp, 8H, *CH*_*2*_-dppe). APT-^13^C{^1^H} NMR [(CD_3_)_2_SO, Me_4_Si] δ/ppm: 132.6 (C_*ipso*_), 132.5 (C_*ortho*_), 130.4 (C_*para*_), 129.1 (C_*meta*_), 39.9 (t, *J*_*CP*_*= 20, −CH*_*2*_*–*). ^31^P{^1^H} NMR [(CD_3_)_2_SO, 293 K] δ/ppm: 3.48 (2 d, ^1^*J*(^109^Ag–^31^P_dppe_) = 267.3, ^1^*J*(^107^Ag–^31^P_dppe_) = 231.7). FTIR [KBr cm^–1^]: 3053 υ(C–H aromatics), 2900 υ(C–H aliphatics), 1608 υ(C=C aromatics), 1435 υ(CH_2_ aliphatics), 1259, 1153, 1028 υ(CF_3_SO_3_^–^). UV–vis [DMSO, λ_max_/nm (ε × 10^3^/M^–1^cm^–1^)]: 281 (20.68). Elemental analysis calc. for C_53_H_48_AgF_3_O_3_P_4_S. C 60.40; H 4.59; S 3.04. Found: C 60.0; H 4.6; S 3.0. ESI-MS (+, *m*/*z*) calc for [C_52_H_48_AgP_4_]^+^: 903.18. Found: 903.45.

### 4.4 X-ray Structure Analysis

The X-ray intensity data were measured on a D8 QUEST ECO three-circle diffractometer system equipped with a PHOTON II CMOS detector, a ceramic X-ray tube (Mo Kα, λ = 0.71076 Å), and a doubly curved silicon crystal Bruker Triumph monochromator.^48^ Measurements were performed at low temperature (100 K). The frames were integrated with the Bruker SAINT software package using a narrow-frame algorithm.^49^ The structure was solved and refined using the Bruker SHELXTL Software Package.^50^ CCDC 2251148 (for (**1**)), 2251149 (for (**2**)), 2251150 (for (**3**)), and 2251151 (for (**4**)) contain the supplementary crystallographic data for this paper. These data can be obtained free of charge from The Cambridge Crystallographic Data Centre via www.ccdc.cam.ac.uk/products/csd/request/.

### 4.5 DFT Calculations

Geometry optimizations were performed in a vacuum using the M06L functional^51^ employing the 6-31+G(d) basis set for all elements except for silver for which the Stuttgart–Dresden basis set (SDD) along with the associated pseudopotential was used. To determine ^31^P NMR chemical shifts relative to phosphoric acid calculated by the GIAO method,^52^ single point calculations were performed on the optimized structure using the same level of theory but employing the larger 6-311G(2d,2p) basis set on all elements apart from silver (SDD basis set) while also including solvent effects (chloroform) through the SMD solvation model.^53^

### 4.6 Stability Studies

All compounds were dissolved in DMSO-*d*_6_ and transferred to an NMR tube. Then, and at set time intervals, the solution behavior (under air and moisture exposure) of all compounds was monitored by measuring the ^1^H and ^31^P{^1^H} NMR spectra for a period of 48 h. All the samples were stored at room temperature and protected from light between measurements.

For the stability evaluation in PBS, 10 mM solutions of complexes **1**, **4**, and **7** were first prepared in DMSO. Two microliters of this solution were then added to PBS (250 μL) for a total concentration of 80 μM. Following centrifugation for 10 min at 3000 rpm, the resulting solutions were monitored by LC-ESI(+)-MS and LC-ESI(+)-HRMS for a period of 48 h.

### 4.7 Cell Culture

In vitro tests were performed with the human cell lines shown in Table S3. Cells were grown in their respective medium supplemented with 10% fetal bovine serum (FBS). All cells were maintained at 37 °C in an atmosphere containing 5% CO_2_ and enriched humidity. Tests for mycoplasma contamination were regularly performed. Moreover, all the cell lines were maintained in culture from re-establishment not more than 10 passages.

### 4.8 Viability Assays

Cells were seeded (2 × 10^4^ cells/well for HCT116, HCT116/OxR, 3 × 10^4^ cells/well for HCT116 p53KO, 4 × 10^4^ cells/well for MES-OV, SK-OV-3, and their respective CBP resistant counterparts) in 100 μL/well in 96-well plates and allowed to attach for 24 h at 37 °C and 5% CO_2_. Compounds were first dissolved in DMSO to a 10 mM stock solution and then further diluted in a growth medium (DMSO concentration <1%). After 24 h, cells were treated with 100 μL of different drugs dilutions in triplicates with the final concentrations of 0, 0.01, 0.05, 0.075, 0.1, 0.5, 0.75, 1, 2.5, 5, 7.5, and 10 μM depending on the compound and the cell line. For combination experiments, compounds were added in 50 μL growth medium and inhibitors (U0126, MAPK inhibitor from Calbiochem, z-VAD-FMK, pan-caspase inhibitor, from Enzo Life Sciences (New York, USA) also in 50 μL medium. After an incubation time of 24 and/or 72 h at 37 °C and 5% CO_2_, the proportion of viable cells was determined by 3-(4,5-dimethylthiazole-2-yl)-2,5-diphenyltetrazolium (MTT) assay following the manufacturer’s recommendations (EZ4U, Biomedica, Vienna, Austria). Anticancer activity was expressed as IC_50_ values (drug concentrations inducing 50% reduction of cell survival in comparison to the control) calculated from full dose–response curves using the GraphPad Prism software.

### 4.9 “In Vitro” Hemolytic Assay

To analyze the extent of hemolysis caused by the drugs, naive blood from male CB17/SCID mice was taken from the abdominal aorta and equally divided in EDTA tubes. Subsequently, the blood was treated with 10 μM of the drugs for 30 min. As a positive control, one sample was treated with a freeze/thaw cycle in liquid nitrogen/37 °C water bath. After incubation, the samples were centrifuged at 300*g* for 5 min, and the cell pellet was washed three times with phosphate-buffered saline (PBS). The pellet was then resuspended in 500 μL PBS, and 100 μL/well was transferred to a 96-well plate. A TECAN Infinite 200 Pro plate reader was used to measure the absorbance at 541 nm.

### 4.10 Clonogenic Assays

For the long-term effect of the drugs, cells were seeded (400 cells/well for SK-OV-3 and 300 cells/well for MES-OV) in 24-well plates for 24 h at 37 °C and 5% CO_2_. Subsequently, cells were treated with the indicated drugs concentration for 10 days. Then, methanol (−20 °C, 20 min) was used to fix the cells, and after a washing step with PBS, cells were stained with crystal violet. The plates were then washed and allowed to dry for 24 h. Fluorescence was measured (633 nm excitation and 610/30 nm bandpass emission filter) with a Typhoon Trio imager (GE Healthcare Life Sciences). The fluorescence intensities were analyzed by ImageJ and, after subtraction of the blank value, were normalized to untreated cells.

### 4.11 Drug Uptake Studies

For the detection of silver levels, inductively coupled plasma-mass spectrometry (ICP-MS) was performed. Cells were seeded (1 × 10^6^ cells/well) in 1 mL/well in six-well plates and, after 24 h recovery time, treated with 10 μM of the drugs for 5 h at 37 °C and 5% CO_2_. Subsequently, the cells were collected by trypsinization, and 100 μL of the harvested cells was separately transferred in fresh tubes and counted for normalization. The remaining cell solutions were transferred into 15 mL Falcons and centrifuged at 300*g* for 5 min. The pellets were washed twice with PBS and dried at room temperature for 15 min. For digestion, the pellet was then resuspended in 100 μL HNO_3_ (69%, Rotipuran Supra, Carl Roth, Karlsruhe, Germany) at room temperature for 1 h. The lysate was then further diluted in 5.9 mL of ddH_2_O. The silver content was measured with an Agilent ICP-MS 7800 (Agilent Technologies, Tokyo, Japan) at the Institute of Inorganic Chemistry, University of Vienna. The instrument was equipped with an Agilent SPS 4 autosampler (Agilent Technologies, Tokyo, Japan) and a MicroMist nebulizer at a sample uptake rate of approximately 0.2 mL/min. The Agilent MassHunter software package (Workstation Software, version C.01.04, Build 544.17, Patch 3, 2018) was used for data processing. The experimental parameters for ICP-MS are summarized in Table S4. The instrument was tuned on a daily basis to achieve maximum sensitivity.

### 4.12 Inhibition of Mammalian TrxR

To determine the inhibition of mammalian TrxR, an established microplate reader-based assay was performed. For this purpose, commercially available recombinant rat TrxR (from Cayman Chemical) was used and diluted with distilled water to achieve a concentration of 0.12 U mL^–1^. The compounds were freshly dissolved as stock solutions in DMSO. Aliquots (25 μL) of the enzyme solution and 25 μL of potassium phosphate buffer (pH 7.0) containing the compounds in graded concentrations (1% DMSO) were mixed. Positive controls were composed of 25 μL aliquots of the enzyme solution mixed with 25 μL 1% DMSO in buffer solution (no compounds). The final concentration of DMSO was 0.5% v/v in all samples. The blank solution was the highest used concentration of the compound in 0.5% DMSO in buffer solution (no enzyme). All resulting solutions were incubated with moderate shaking for 75 min at 37 °C in a 96-well plate. To each well, a 225 μL reaction mixture (1 mL reaction mixture consists of 500 μL potassium phosphate buffer (pH 7.0), 80 μL EDTA solution (100 mM, pH 7.5), 20 μL bovine serum albumin solution (0.2%), 100 μL of NADPH solution (20 mM), and 300 μL distilled water) was added, and the reaction was started immediately by addition of 25 mL of a 20 mM ethanolic 5,5′-dithiobis(2-nitrobenzoic acid) solution. After proper mixing, the formation of 2-nitro-5-thiobenzoate (5-TNB) was monitored with a microplate reader at 405 nm 10 times in 35 s intervals for about 6 min. The increase in 5-TNB concentration over time followed a linear trend, and the enzymatic activities were calculated as the slopes (increase in absorbance per second) thereof. For each tested compound, the noninterference with the assay components was confirmed as there was no TNB formation with the blank solution. The IC_50_ values were calculated as the concentration of compound decreasing the enzymatic activity of the untreated control by 50% and are given as the means and error of two repeated experiments. All reagents were obtained from Sigma-Aldrich except where otherwise specified. For the absorption measurements in the enzyme assay, a PerkinElmer 2030 Multilabel Reader VICTORTMX4 was used.

### 4.13 Flow Cytometry

Cells were seeded in a density of (2–4) × 10^5^ cells/well in six-well plates and in 1 mL culture medium containing 10% FBS. After 24 h recovery, cells were treated with the indicated compounds and concentrations. Next, the supernatant was collected followed by trypsinization of the cells and centrifugation at 300*g* for 5 min. In the following protocols, cells were stained with annexin V (AV) and propidium iodide (PI) for cell death analysis, with only PI for cell cycle analysis, or with 10 μg mL^–1^ JC-1 for evaluation of the mitochondrial membrane potential as previously described.^54,55^

### 4.14 Western Blot

For protein detection, cells were treated with the indicated concentrations for 24 h. After preparation, protein lysates were used for the SDS-PAGE, blotted on a polyvinylidene difluoride (PVDF) membrane, and stained with primary antibodies overnight followed by secondary antibodies for 1 h as previously described.^56^ The following primary antibodies were diluted (1:1000): Sigma-Aldrich: β-actin (AC-15; #A5441; diluted 1:2000), PARP (46D11; #9532), Cleaved PARP (Asp2149) (D64E10, #5625), Phospho-Histone H2A.X (Ser139) (20E3, #9718), Calnexin (#2679), PDI (#3501), ero1L-α (#3264), BiP (#3177), and IRE1α (#3294). The following secondary antibodies were diluted (1:10,000): antimouse (#7076) and antirabbit (#7074) horseradish peroxidase-labeled antibodies from Cell Signaling Technologies.

### 4.15 DNA Fragmentation

MES-OV cells (5 × 10^5^ cells/mL) were incubated for 24 h with the indicated concentration. All cells were centrifuged at 300*g* for 5 min, washed with PBS, and centrifuged again. The pellet was resuspended in DNA lysis buffer (50 mM Tris (pH 8), 10 mM EDTA, and 0.5% sodium lauryl sarcosine) followed by addition of 20 U of RNAase solution (Sigma) and incubation at 37 °C for 1 h. Finally, 150 μg of proteinase K (Sigma) was added and incubated overnight. Isolated DNA was analyzed by electrophoresis (80 V, 1 h) in 2% agarose gels containing ethidium bromide (Sigma).

### 4.16 Microscopy

Cells were seeded 5 × 10^5^/well in six-well plates after 24 h recovery treatment with compounds **1**, **4**, and **7** was performed with the indicated concentrations. After 6 and 24 h, phase-contrast images of the cells were taken with a Zeiss Primovert microscope (20× magnification). To determinate the percentage of vacuolated cells, images (three images each condition) were analyzed by ImageJ, and vacuolated cells were counted.

### 4.17 Spinning-Disk Confocal Microscopy

Cells were seeded 10 × 10^4^ cells/mL for SW480 ER-YFP (SW480 subclone transfected with an ER-tracked YFP) and 15 × 10^4^ cells/mL for MES-OV in 200 μL/well in eight-well chamber slides (Ibidi, Martinsried, Germany). After 24 h, cells were treated with 100 μL of different drug dilutions, with the final concentrations of 0, 5, and 10 μM, depending on the compound. For ER and mitochondria tracking, the medium was replaced, and MES-OV cells were stained with MitoTracker Red CMXRos, MitoTracker Green^FM^, or ER-Tracker Red (ThermoFisher Scientific) in serum- and phenol red-free medium for 30 min at 37 °C. After 30 min, trackers were removed, and cells were washed and kept in a serum- and phenol red-free medium for life-cell imaging. A Spinning-Disk Confocal Super Resolution Microscope (Olympus) was used to take images. Three representative pictures were taken in confocal mode, *Z*-stack, and max intensity projection (192× magnification and 60x) of vesicles in ER-YFP-transfected SW480 (ER-YFP in green), DIC (differential interference contrast), and mitochondria (MitoTracker in red or green). Contrast and brightness were adjusted with Cellsens dimension 4.0.

### 4.18 TEM

MES-OV Cells were seeded (8 × 10^5^ cells/mL) in 100 μL/well in six-well plates. After 24 h, cells were treated with the selected compounds at the indicated concentration for another 24 h. Afterward, cells were collected, pelleted, and fixed in a solution of 2.5% glutaraldehyde and 2% paraformaldehyde in 0.1 M cacodylate buffer (pH 7.4) for 2 h at room temperature. Samples were subsequently washed 3 × 10 min in cacodylate buffer and postfixed in 1% osmium tetroxide for 1 h. After 3 × 10 min washing in cacodylate buffer, samples were dehydrated in an ethanol series (30–100%, 10 min each) and pure acetone (2 × 10 min) and gradually embedded in Epon 812 resin (Serva). Sections (40–50 nm) were prepared with a Leica UC7 ultramicrotome, mounted on copper mesh grids, and contrasted with 1% uranyl acetate (7 min) and 3% lead citrate (3 min). Imaging was performed with a Tecnai G2 20 transmission electron microscope at 80 kV equipped with an FEI Eagle 4K CCD camera (Center for Anatomy and Cell Biology). Image processing was performed in Fiji^57^ using the CLAHE filter (enhance local contrast).

### 4.19 Detection of ROS

Cells were seeded 5 × 10^5^ cells/mL in six-well plates. After 24 h treatment with the indicated concentration, cells were incubated with 10 μM 2′,7′-dichlorofluorescein diacetate (DCFH-DA, Sigma-Aldrich) in Hank’s balanced salt solution for 1 h at 37 °C. Subsequently, flow cytometry (with BD LSR Fortessa instrument, BD Biosciences, Franklin Lakes, NJ, USA) was performed to measure the median fluorescence signal of 20,000 single cells per sample. FlowJo (BD Biosciences, San Jose, CA, USA) and GraphPad Prism software (GraphPad Software Inc., San Diego, CA, USA) were to analyze the data.

### 4.20 Glutathione Measurement

Cells were seeded 4 × 10^4^ cells/mL for SK-OV-3 and 4.5 × 10^4^ cells/mL for MES-OV in 100 μL/well in 96-well plates. After 24 h, cells were treated with the selected compounds (1, 2.5, and 5 μM) in triplicates for another 24 h. Afterward, cells were lysed, and a GSH/GSSG-GloTM Kit (#V6611, Promega, Madison, USA) was used to measure the levels of total and oxidized glutathione following the manufacturer’s instructions. A TECAN Infinite 200 Pro plate reader was used to measure the luminescence. Untreated control values were used to determine the fold increase in relative luminescence units (RLU).

## Data Availability

The authors will release the atomic coordinates upon article publication.

## Supporting Information Available

Characterization of compounds **1**–**7** (HR-MS, UV–vis, and NMR spectra); crystallographic data and structural details for X-ray data for **1**, **2, 3**, and **4**; stability studies in 100% DMSO-*d*_6_ performed by ^1^H and ^31^P{^1^H} NMR experiments; and biological data regarding the anticancer activity of **1**–**7** against OC and CRC cell lines (IC_50_ curves, cell cycle arrest, cell death mechanism) (PDF)
Molecular formula strings (CSV)

## Abbreviations Used

AV/PI: annexin V/propidium iodide
Ag(I)-NHCs: silver(I)-*N*-heterocyclic carbenes
BiP: immunoglobulin binding protein
Bipy: 2,2′-bipyridine
DMSO: dimethyl sulfoxide
DCFH-DA: dichlorodihydrofluorescein diacetate
DFT: density functional theory
Dppe: 1,2-bis(diphenylphosphino)ethane
ER: endoplasmatic reticulum
Ero1-Lα: endoplasmic reticulum oxidoreductase 1 alpha
EDTA: ethylenediaminetetraacetic acid
FBS: fetal bovine serum
FTIR: Fourier-transform infrared spectroscopy
GRP78: glucose-regulating protein 78
GSH: glutathione
GSSG: glutathione disulfide
IC_50_: half-maximal inhibitory concentration
ICP-MS: inductively coupled plasma-mass spectrometry
IRE1-α: inositol-requiring transmembrane kinase/endoribonuclease 1 alpha
LC-ESI: liquid chromatography electrospray ionization
MAPK: mitogen-activated protein kinase
MTT: 3-(4,5-dimethylthiazole-2-yl)-2,5-diphenyltetrazolium
NADPH: nicotinamide adenine dinucleotide phosphate hydrogen
NMR: nuclear magnetic resonance
OC: ovarian cancer
PARP: poly(ADP-ribose) polymerase
PBS: phosphate-buffered saline
PPh_3_: triphenylphosphane
P53: tumor protein 53
PDI: protein-disulfide isomerase
PVDF: polyvinylidene difluoride
RLU: relative luminescence units
ROS: reactive oxygen species
RNAase: ribonuclease
SI: Supporting Information
TrxR: thioredoxin reductase
TEM: transmission electron microscopy
VT-NMR: variable temperature nuclear magnetic resonance
5-TNB: 2-nitro-5-thiobenzoate
