# Supplementary material for: Paraptotic Cell Death as an Unprecedented Mode of Action Observed for New Bipyridine-Silver(I) Compounds Bearing Phosphane Coligands
Source: J Med Chem. 2024 Feb 24;67(8):6081–98. doi: 10.1021/acs.jmedchem.3c01036 (PMC11056982; doi:10.1021/acs.jmedchem.3c01036)
Supplement: Supplementary file 1 — jm3c01036_si_001.pdf [file jm3c01036_si_001.pdf]

Supporting Information

Paraptotic cell death as unprecedented mode of action observed for new bipyridine-silver(I) compounds bearing phosphane co-ligands

Ricardo G. Teixeira,[a, †] Alessia Stefanelli,[b, †] Adhan Pilon,[a, †] Rebecca Warmers,[b] Xavier Fontrodona,[c] Isabel Romero,[c] Paulo J. Costa,[d] Maria J. Villa de Brito,[a] Xenia Hudec,[b] Christine Pirker,[b] Sebastian Türck,[e] Alexandra M. M. Antunes,[f] Christian R. Kowol,[g] Ingo Ott,[e] Anamaria Brozovic,[h] Andy Sombke,[i] Margret Eckhard,[i] Ana Isabel Tomaz,[a] Petra Heffeter,[b,*] Andreia Valente[a,*]

[a] Centro de Química Estrutural, Institute of Molecular Sciences, Departamento de Química e Bioquímica, Faculdade de Ciências, Universidade de Lisboa, Campo Grande, 1749-016 Lisboa, Portugal.

[b] Center for Cancer Research and Comprehensive Cancer Center, Medical University of Vienna, 1090 Vienna, Austria.

[c] Departament de Química and Serveis Tècnics de Recerca, Universitat de Girona, Campus de Montilivi, 17071 Girona, Spain.

[d] BioISI - Instituto de Biosistemas e Ciências Integrativas, Faculdade de Ciências, Universidade de Lisboa, 1749-016, Lisboa, Portugal.

[e] Institute of Medicinal and Pharmaceutical Chemistry, Technische Universität Braunschweig, Beethovenstr. 55, 38106 Braunschweig, Germany.

[f] Centro de Química Estrutural (CQE), Institute of Molecular Sciences, Departamento de Engenharia Química, Instituto Superior Técnico (IST), Universidade de Lisboa, Av Rovisco Pais 1, 1049-001 Lisboa, Portugal

[g] Institute of Inorganic Chemistry, Faculty of Chemistry, University of Vienna, Waehringerstrasse 42, 1090 Vienna, Austria

[h] Division of Molecular Biology, Ruđer Bošković Institute Bijenička cesta 54, 10000 Zagreb, Croatia.

[i] Center for Anatomy and Cell Biology, Cell and Developmental Biology, Medical University of Vienna, Schwarzspanierstraße 17, 1090 Vienna, Austria

[†]These authors contributed equally to this paper

Corresponding authors

*E-mail: amvalente@fc.ul.pt; petra.heffeter@meduniwien.ac.at

List of contents

List of contents 3

S1. Electronic spectra 4

S2. NMR spectra 5

S3. Single crystal X-ray crystallography 16

S4. Stability studies 22

S5. Biological data 28

S1. Electronic spectra

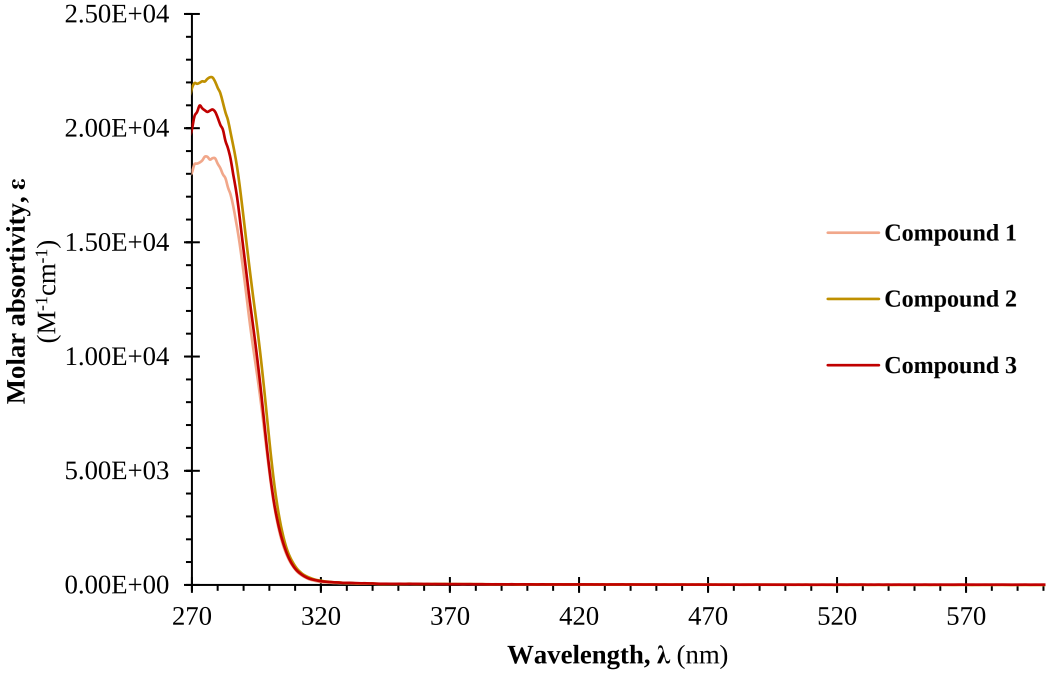

Figure S1. UV-Vis spectra of compounds **1–3** in dimethylsulfoxide.

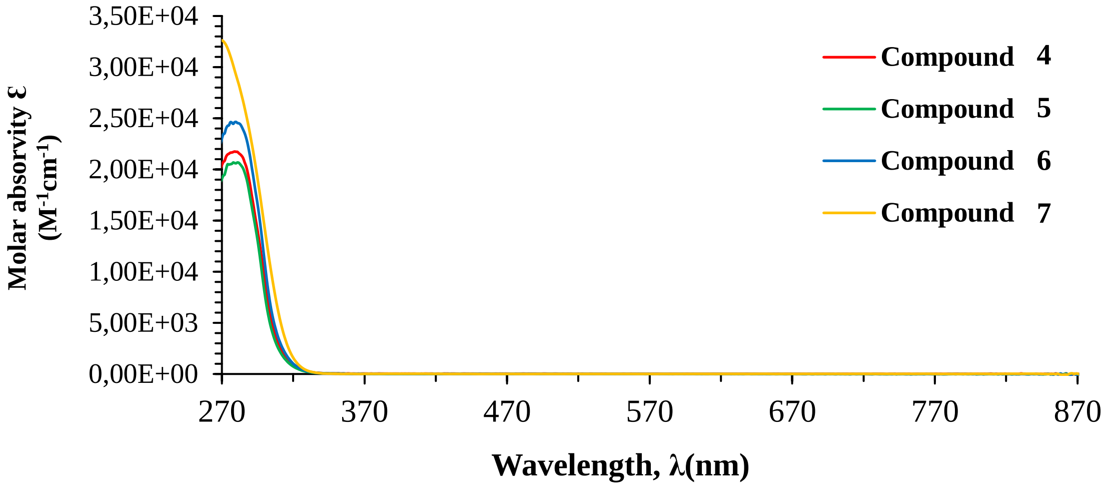

Figure S2. UV-Vis spectra of compounds **4–7** in dimethylsulfoxide.

S2. NMR spectra

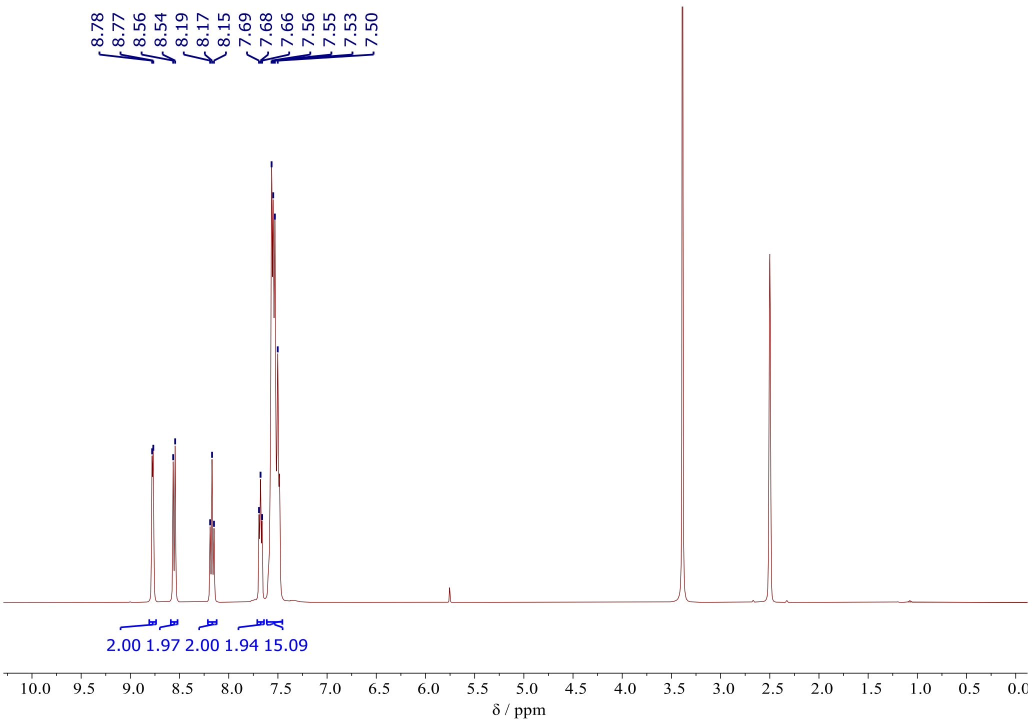

Figure S3. ^{1}H-NMR spectrum of **1** in DMSO-d_6 at 298 K.

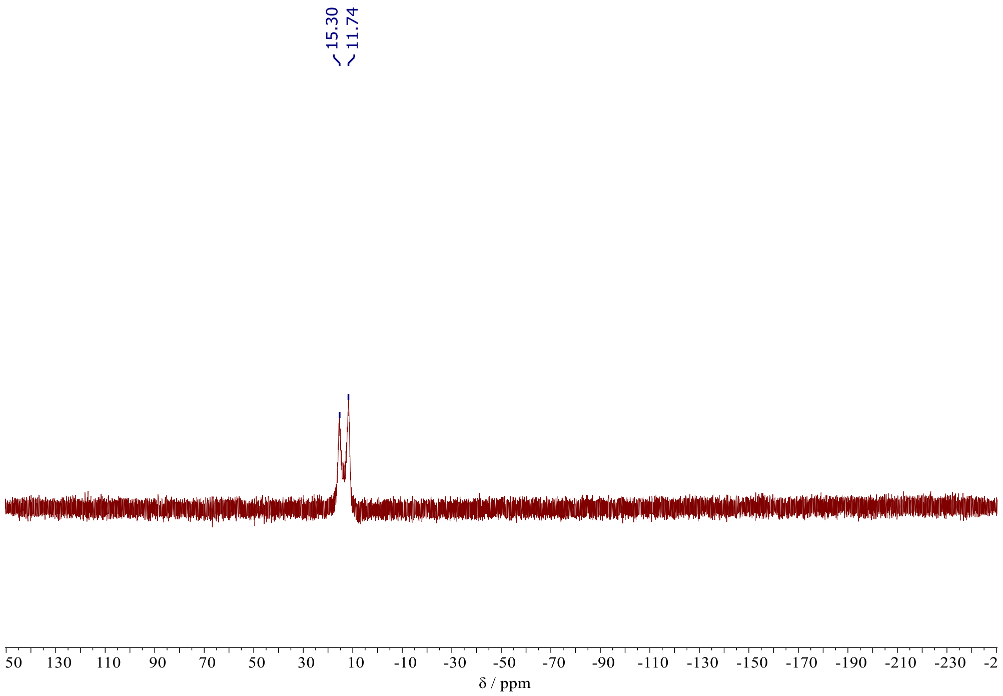

Figure S4. $^{31}P\{^{1}H\}$-NMR spectrum of **1** in DMSO-d_6 at 298 K.

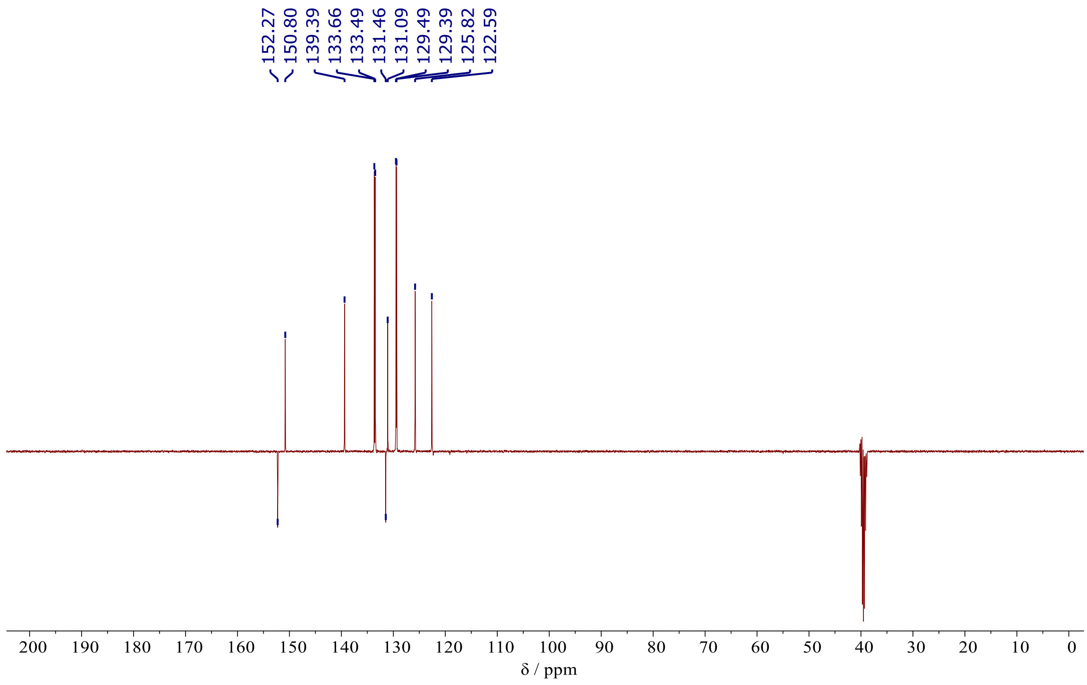

Figure S5. APT $^{13}C\{^{1}H\}$-NMR spectrum of **1**in DMSO-d_6 at 298 K.

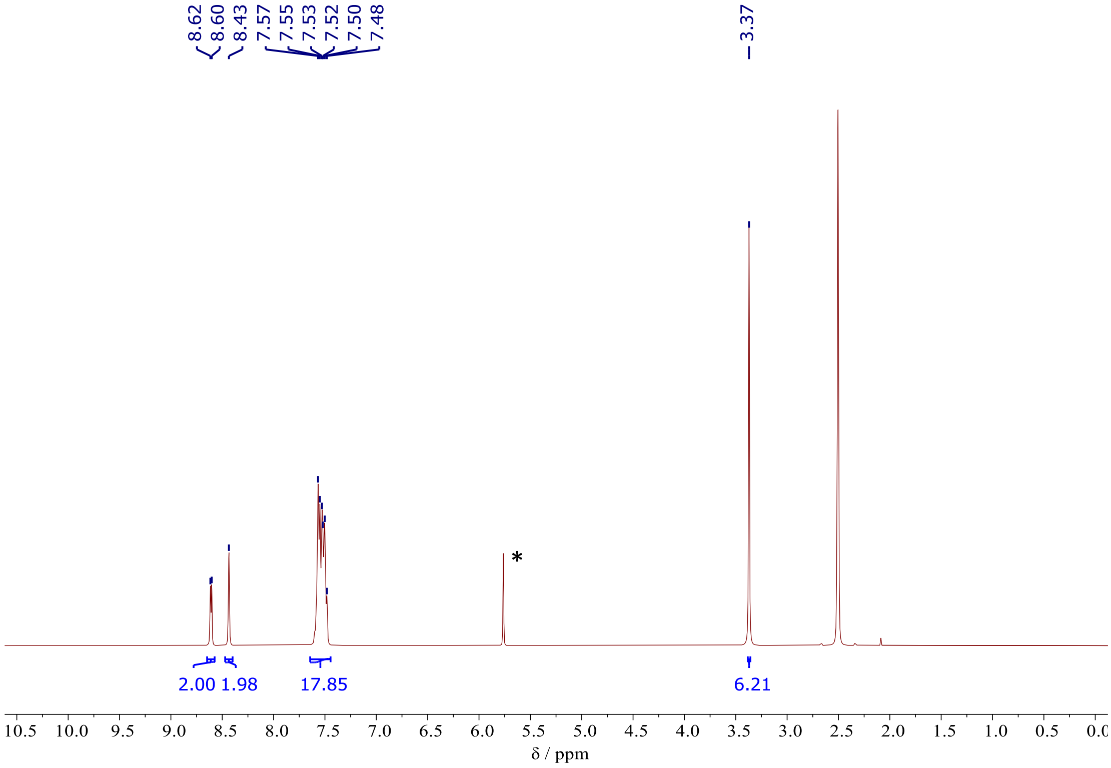

Figure S6. ^{1}H-NMR spectrum of compound **2** in DMSO-d_6 at 298 K. * residual CH_2Cl_2

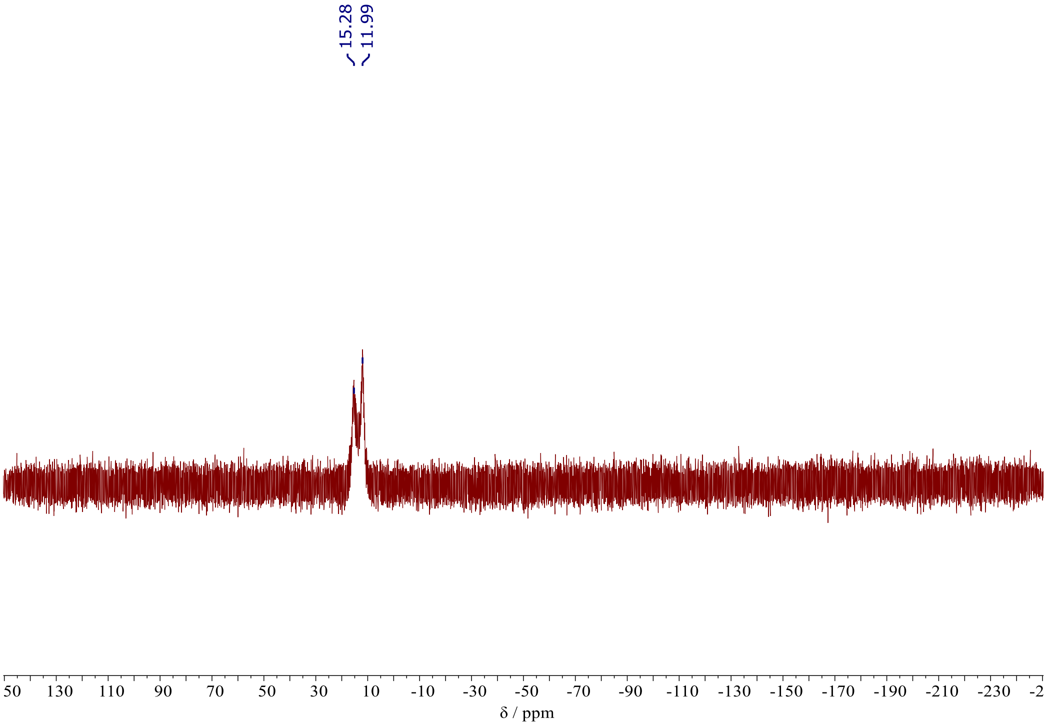

Figure S7. $^{31}P\{^{1}H\}$-NMR spectrum of compound **2** in DMSO-d_6 at 298 K.

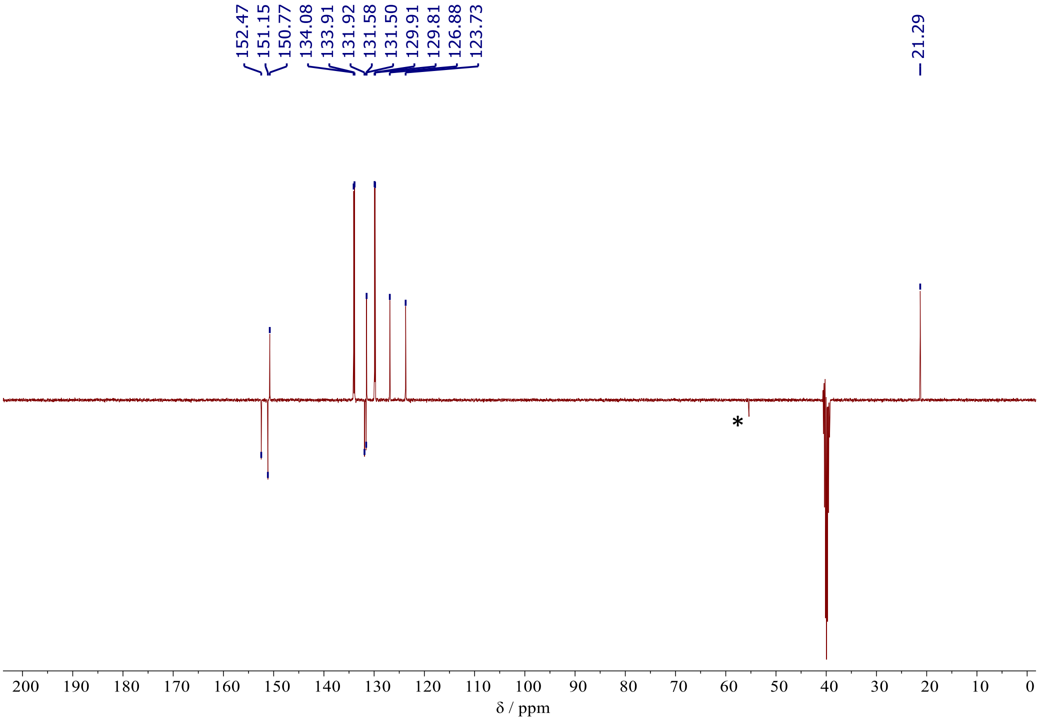

Figure S8. APT $^{13}C\{^{1}H\}$-NMR spectrum of compound **2** in DMSO-d_6 at 298 K. * residual CH_2Cl_2

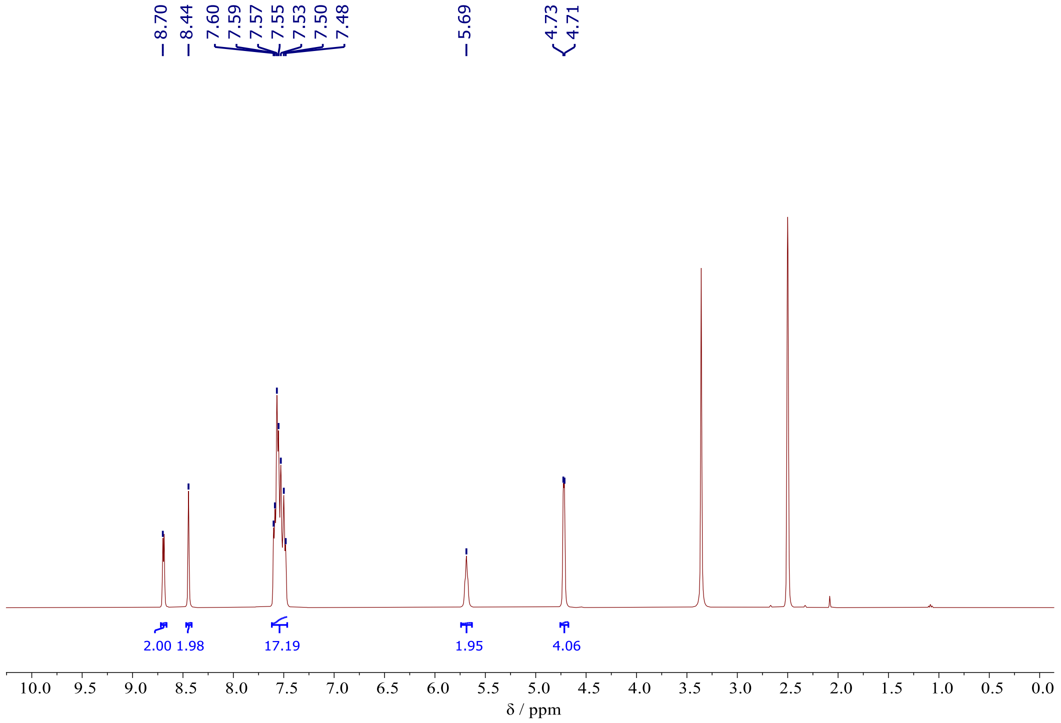

Figure S9. ^{1}H-NMR spectrum of compound **3** in DMSO-d_6 at 298 K.

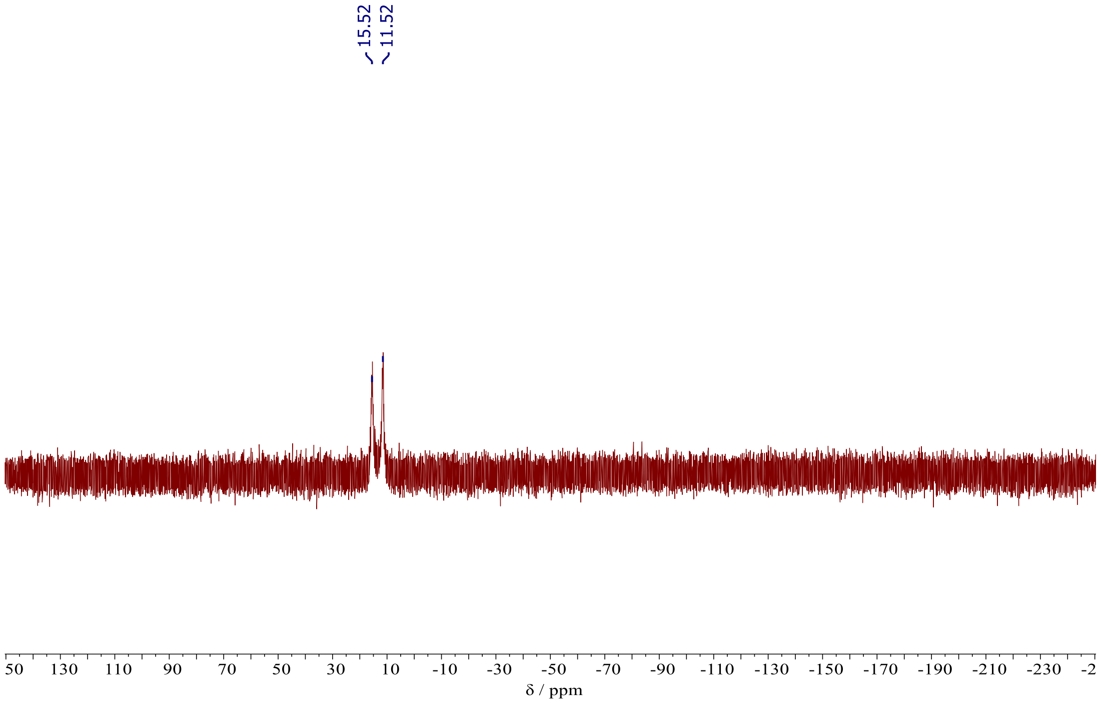

Figure S10. ^{31}P{^{1}H}-NMR spectrum of compound **3** in DMSO-d_6 at 298 K.

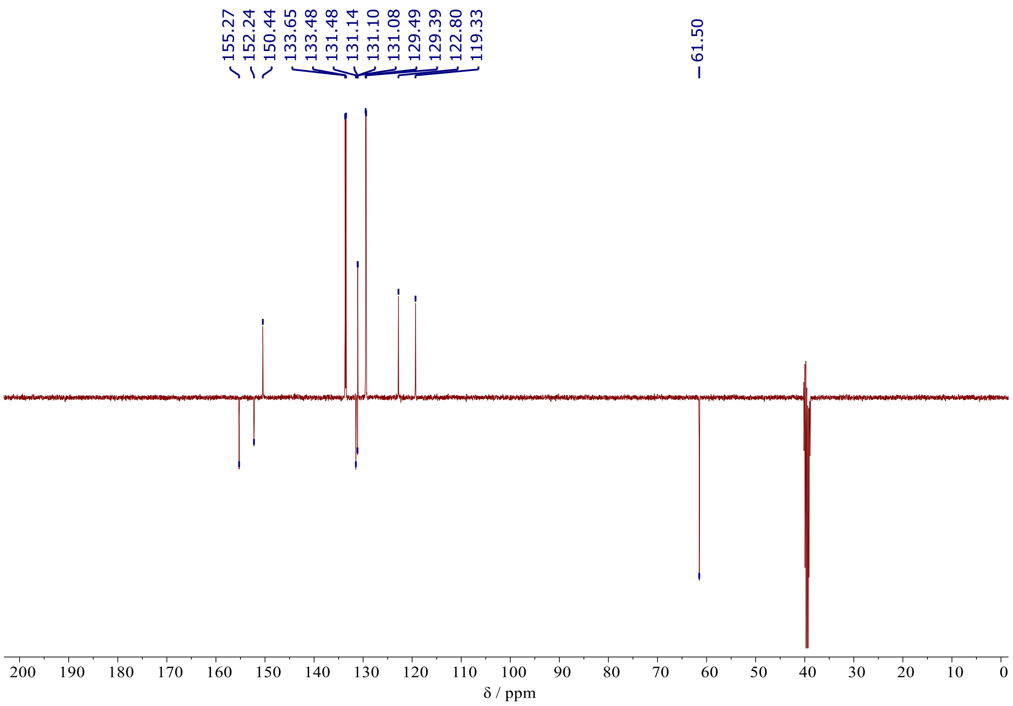

Figure S11. APT $^{13}C\{^{1}H\}$-NMR spectrum of compound **3** in DMSO-d_6 at 298 K.

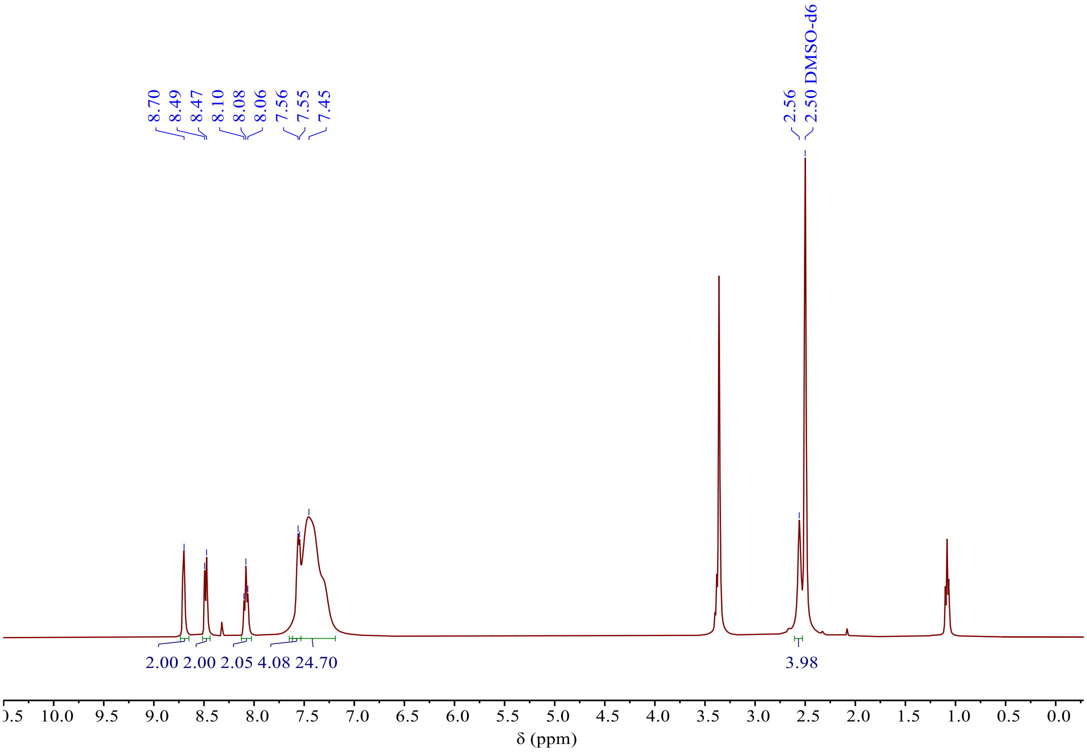

Figure S12. ^{1}H-NMR spectrum of compound **4** in DMSO-d_6 at 298 K.

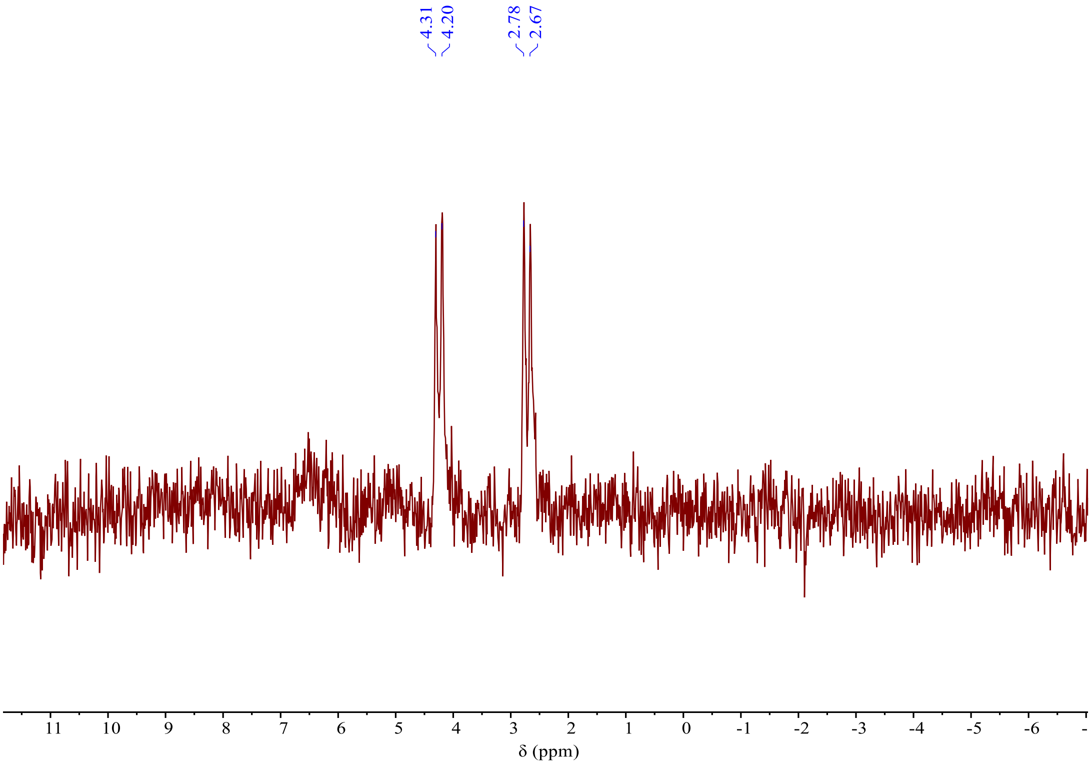

Figure S13. $^{31}P\{^{1}H\}$-NMR spectrum of compound **4** in DMSO-d_6 at 298 K.

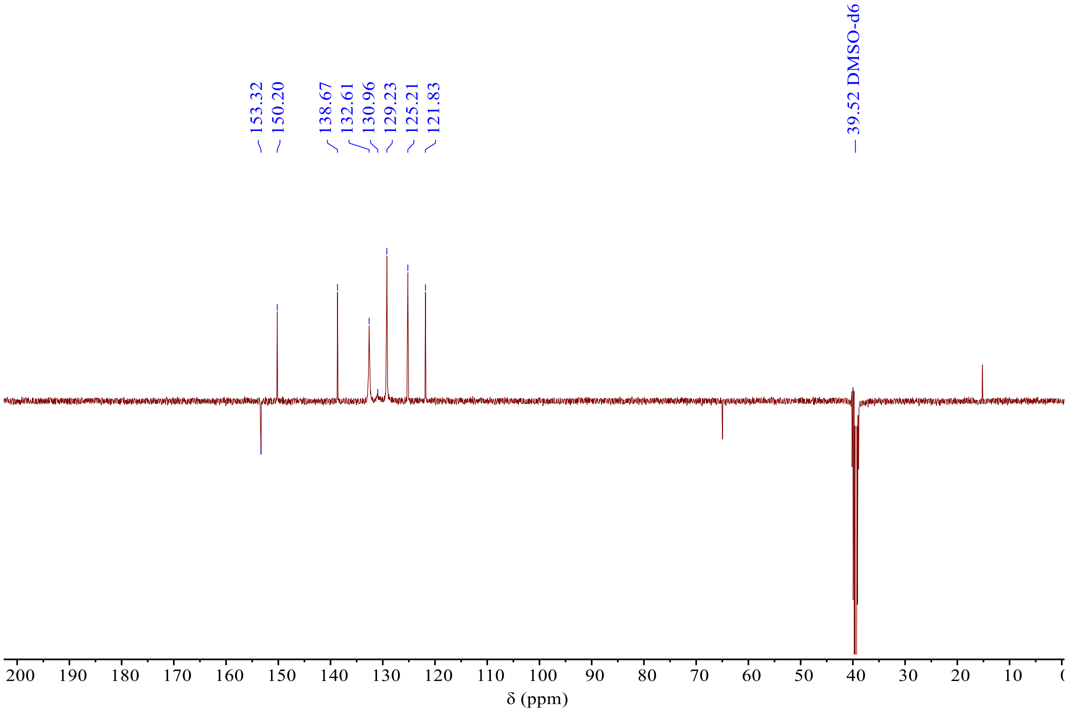

Figure S14. APT $^{13}C\{^{1}H\}$-NMR spectrum of compound **4** in DMSO-d_6 at 298 K.

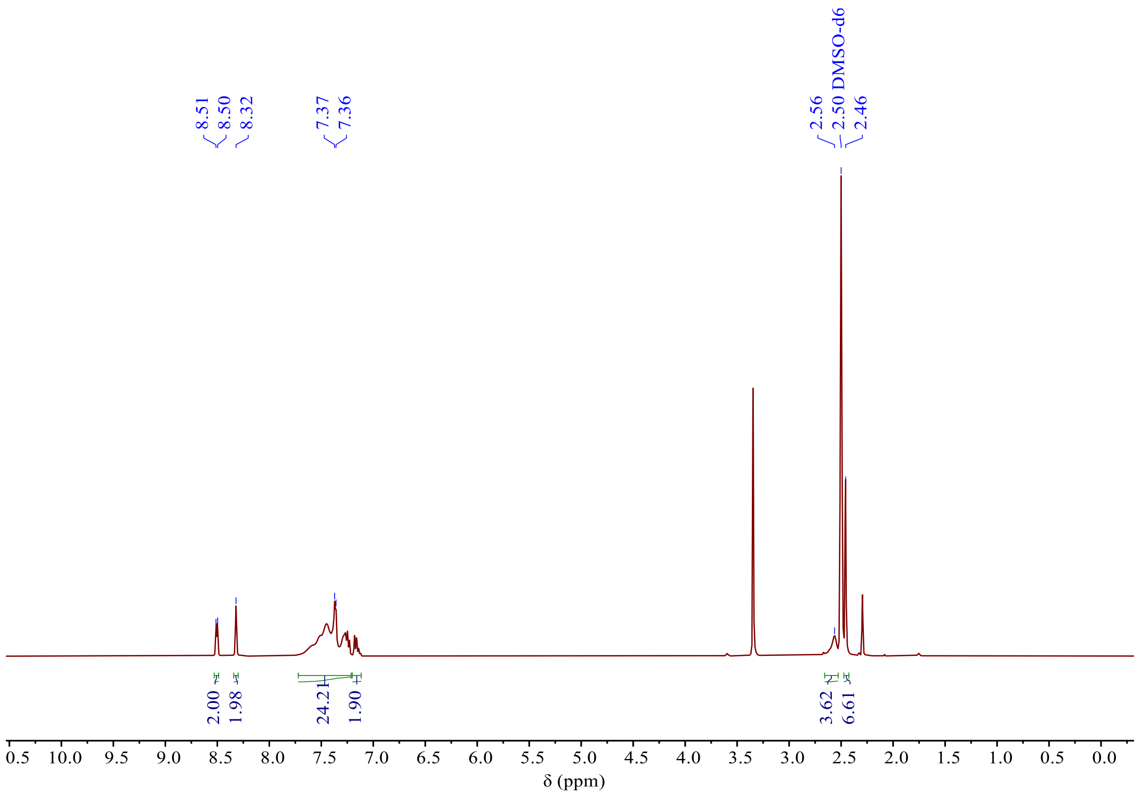

Figure S15. ^{1}H-NMR spectrum of compound **5** in DMSO-d_6 at 298 K.

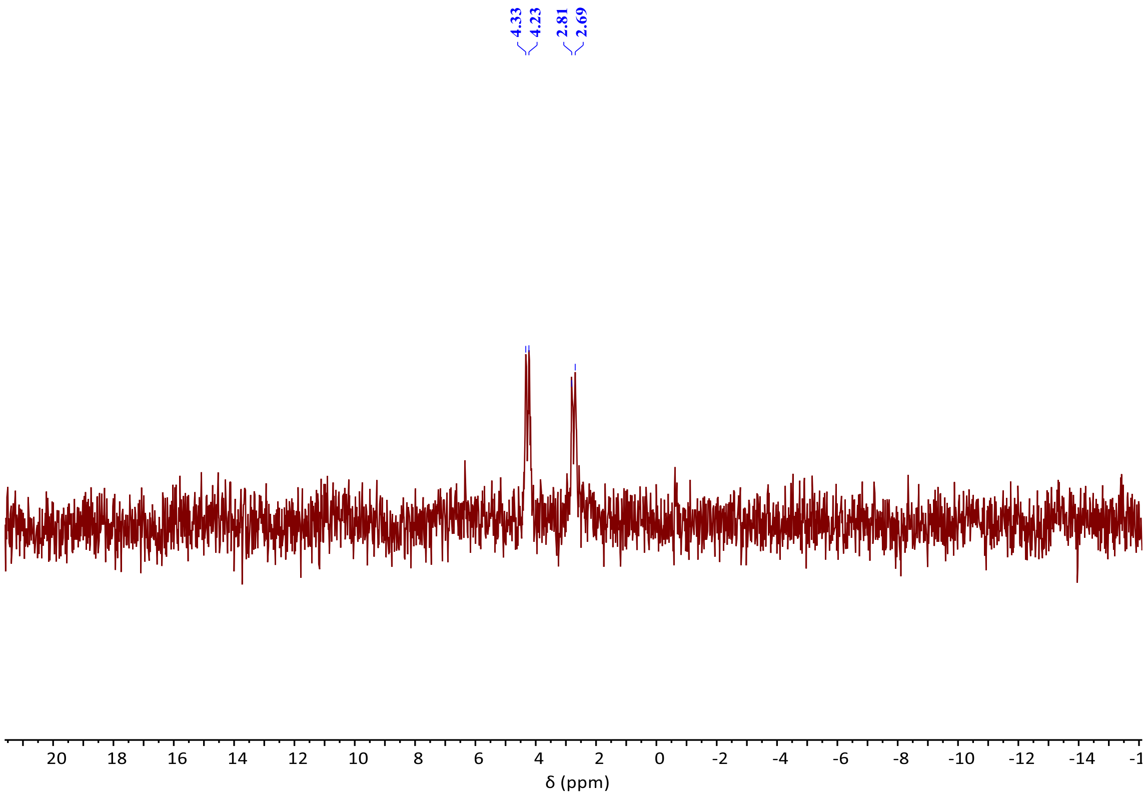

Figure S16. ^{31}P{^{1}H}-NMR spectrum of compound **5** in DMSO-d_6 at 298 K.

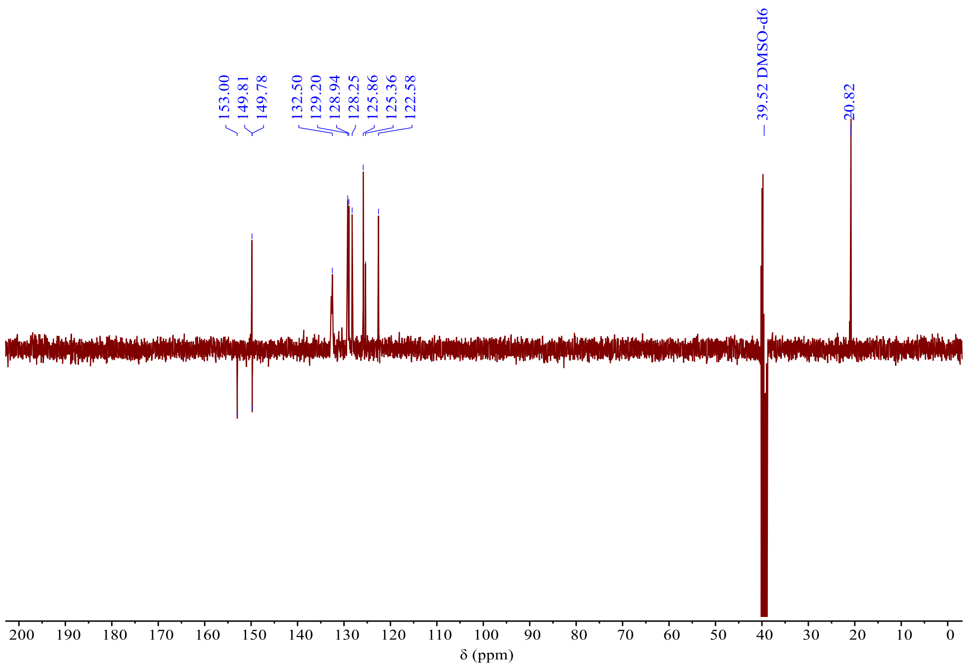

Figure S17. APT $^{13}C\{^{1}H\}$-NMR spectrum of compound **5** in DMSO-d_6 at 298 K.

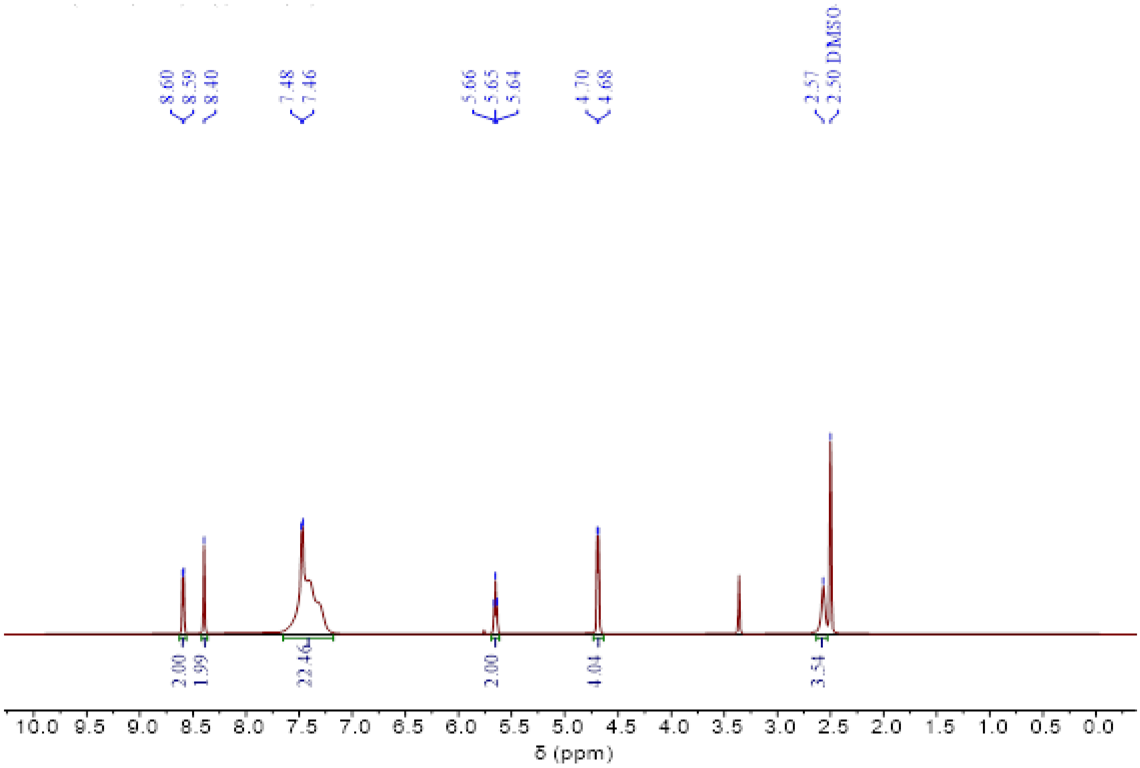

Figure S18. ^{1}H-NMR spectrum of compound **6** in DMSO-d_6 at 298 K.

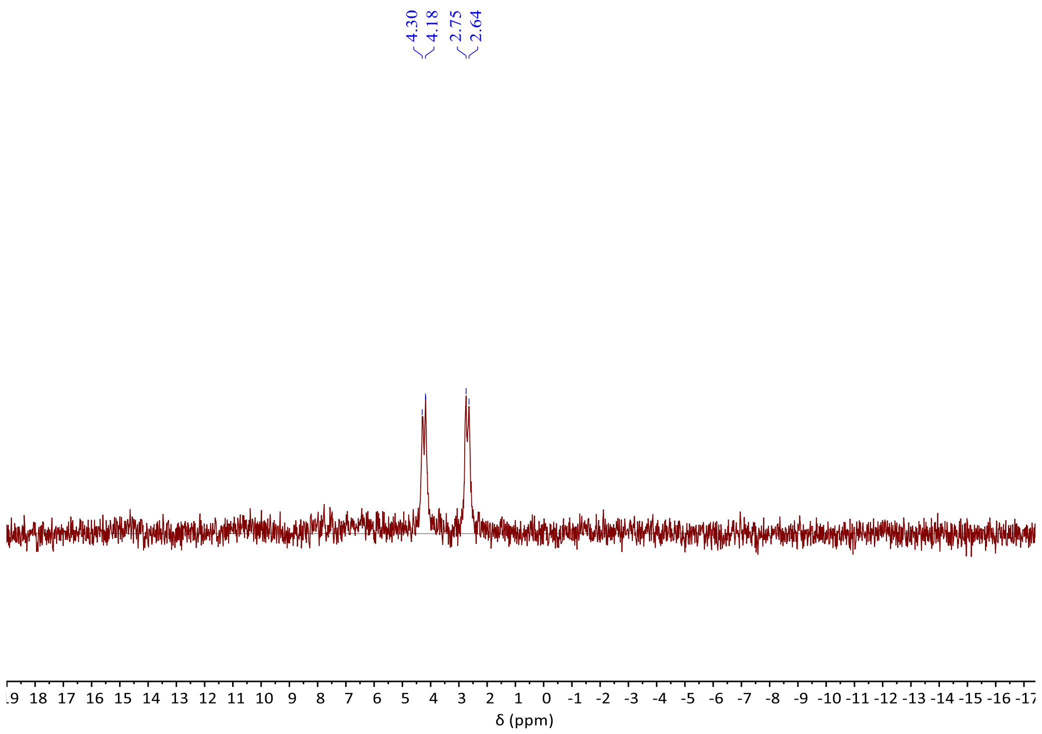

Figure S19. $^{31}P\{^{1}H\}$-NMR spectrum of compound **6** in DMSO-d_6 at 298 K.

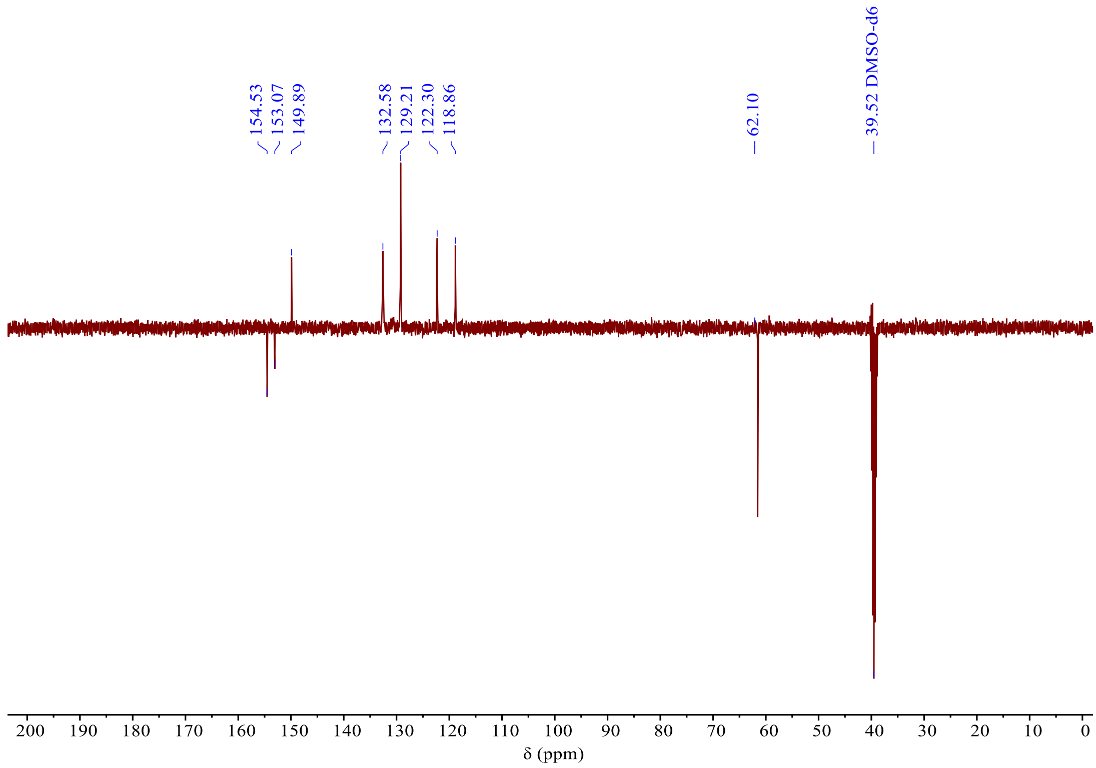

Figure S20. APT $^{13}C\{^{1}H\}$-NMR spectrum of compound **6** in DMSO-d_6 at 298 K.

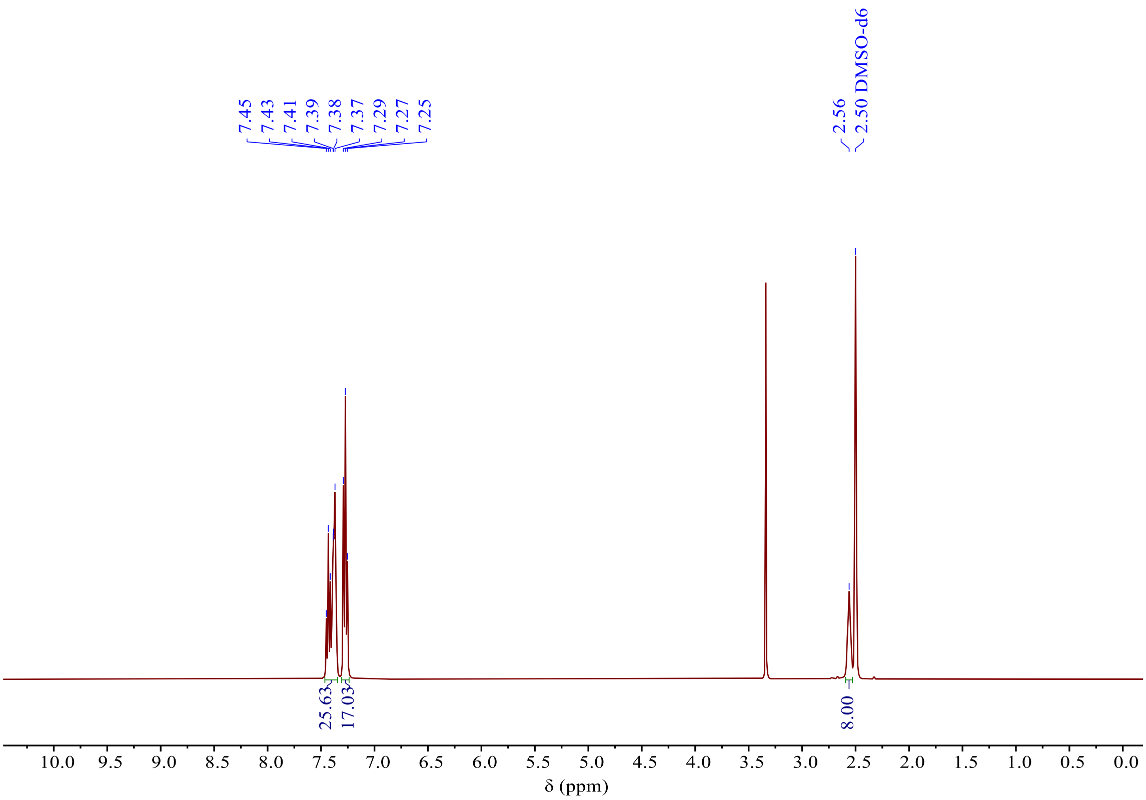

Figure S21. ^{1}H-NMR spectrum of compound **7** in DMSO-d_6 at 298 K.

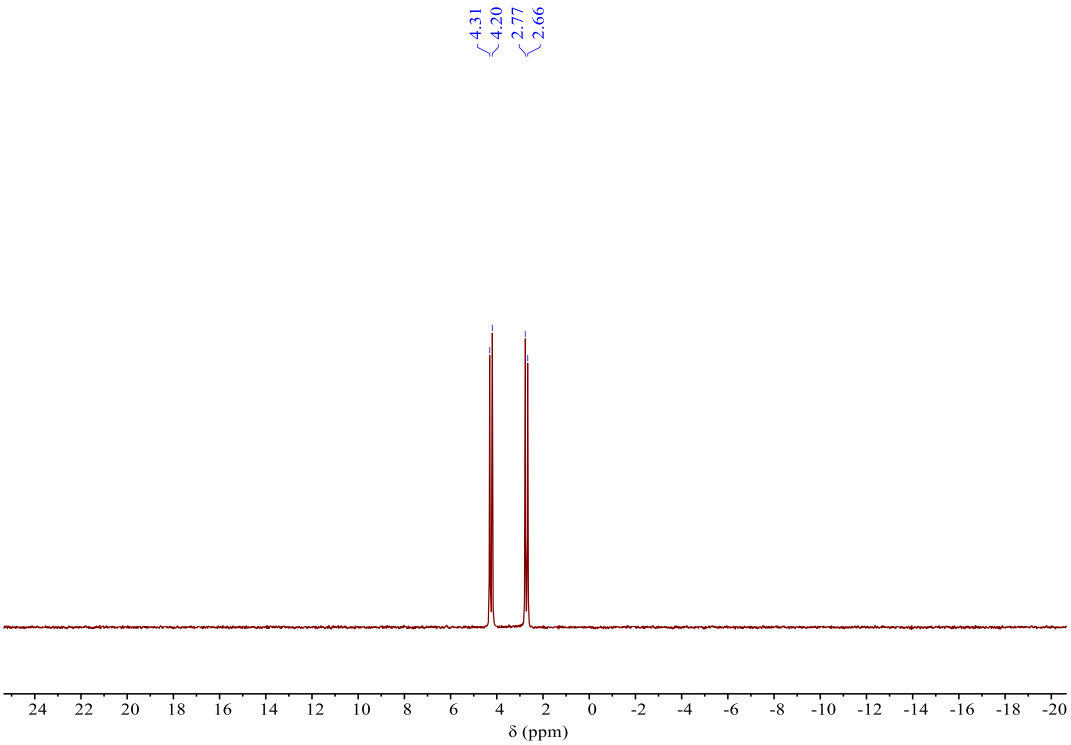

Figure S22. $^{31}P\{^{1}H\}$-NMR spectrum of compound **7** in DMSO-d_6 at 298 K.

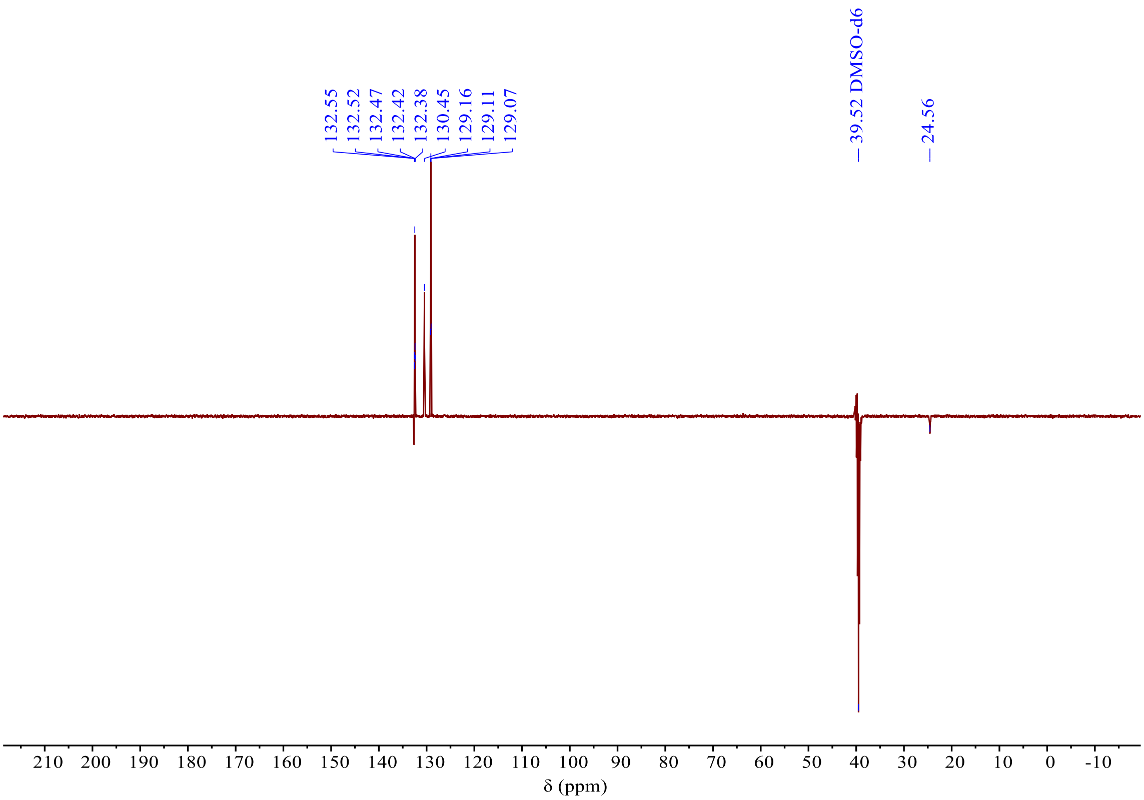

Figure S23. APT $^{13}C\{^{1}H\}$-NMR spectrum of compound **7** in DMSO-d_6 at 298 K.

S3. Single crystal X-ray crystallography

[Ag(2,2'-bipy)(PPh$_3$)][CF$_3$SO$_3$] (**1**)

A total of 637 frames were collected. The integration of the data using a triclinic unit cell yielded a total of 17832 reflections to a maximum θ angle of 27.48° (0.77 Å resolution), of which 6174 were independent (average redundancy 2.888, completeness = 99.3%, R_{int} = 2.56%, R_{sig} = 3.15%) and 5332 (86.36%) were greater than 2σ(F^2). The final cell constants of a = 11.223(6) Å, b = 11.328(7) Å, c = 11.622(7) Å, α = 79.811(16)°, β = 73.258(17)°, γ = 74.41(2)°, volume = 1354.9(14) $Å^3$, are based upon the refinement of the XYZ-centroids of 9962 reflections above 20 σ(I) with 6.012° < 2θ < 55.02°. Data were corrected for absorption effects using the Multi-Scan method (SADABS). The ratio of minimum to maximum apparent transmission was 0.905. The calculated minimum and maximum transmission coefficients (based on crystal size) are 0.6745 and 0.7456. Refinement using the space group P-1, with Z = 2 for the formula unit, $C_{29}H_{23}AgF_3N_2O_3PS$. The final anisotropic full-matrix least-squares refinement on F^2 with 361 variables converged at R1 = 2.68%, for the observed data and wR2 = 5.65% for all data. The goodness-of-fit was 1.119. The largest peak in the final difference electron density synthesis was 0.608 $e^-/Å^3$ and the largest hole was -0.443 $e^-/Å^3$ with an RMS deviation of 0.081 $e^-/Å^3$. On the basis of the final model, the calculated density was 1.655 g/cm^3 and F(000), 680 e^-.

[Ag(4,4'-CH$_3$-2,2'-bipy)(PPh$_3$)][CF$_3$SO$_3$] (**2**)

A total of 2418 frames were collected. The integration of the data using a monoclinic unit cell yielded a total of 165841 reflections to a maximum θ angle of 30.53° (0.70 Å resolution), of which 10342 were independent (average redundancy 16.036, completeness = 99.7%, R_{int} = 4.83%, R_{sig} = 1.96%) and 8924 (86.29%) were greater than 2σ(F^2). The final cell constants of a = 12.711(12) Å, b = 30.03(3) Å, c = 9.239(9) Å, β

= 105.90(2)°, volume = 3392.(6) Å^3, are based upon the refinement of the XYZ-centroids of 9721 reflections above 20 σ(I) with 6.143° < 2θ < 60.98°. Data were corrected for absorption effects using the Multi-Scan method (SADABS). The ratio of minimum to maximum apparent transmission was 0.912. The calculated minimum and maximum transmission coefficients (based on crystal size) are 0.8420 and 0.9520. Refinement using the space group P 1 21/c 1, with Z = 4 for the formula unit, $C_{32}H_{28}AgCl_3F_3N_2O_3PS$. The final anisotropic full-matrix least-squares refinement on F^2 with 484 variables converged at R1 = 3.63%, for the observed data and wR2 = 8.32% for all data. The goodness-of-fit was 1.087. The largest peak in the final difference electron density synthesis was 0.974 e^-/Å^3 and the largest hole was -1.054 e^-/Å^3 with an RMS deviation of 0.080 e^-/Å^3. On the basis of the final model, the calculated density was 1.611 g/cm^3 and F(000), 1656 e^-.

[Ag(4,4'-CH_2OH-2,2'-bipy)(PPh_3)][CF_3SO_3] (**3**)

A total of 2714 frames were collected. The integration of the data using a triclinic unit cell yielded a total of 185765 reflections to a maximum θ angle of 33.17° (0.65 Å resolution), of which 11614 were independent (average redundancy 15.995, completeness = 99.6%, R_{int} = 2.93%, R_{sig} = 1.16%) and 10676 (91.92%) were greater than 2σ(F^2). The final cell constants of a = 8.493(5) Å, b = 10.683(7) Å, c = 17.190(10) Å, α = 97.63(3)°, β = 93.26(2)°, γ = 98.273(19)°, volume = 1525.2(16) Å^3, are based upon the refinement of the XYZ-centroids of 9519 reflections above 20 σ(I) with 7.102° < 2θ < 66.25°. Data were corrected for absorption effects using the Multi-Scan method (SADABS). The ratio of minimum to maximum apparent transmission was 0.909. The calculated minimum and maximum transmission coefficients (based on crystal size) are 0.6782 and 0.7465. Refinement using the space group P-1, with Z = 2 for the formula

unit, $C_{31}H_{27}AgF_3N_2O_5PS$. The final anisotropic full-matrix least-squares refinement on F^2 with 398 variables converged at R1 = 3.51%, for the observed data and wR2 = 8.13% for all data. The goodness-of-fit was 1.132. The largest peak in the final difference electron density synthesis was 2.579 $e^-/Å^3$ and the largest hole was -1.890 $e^-/Å^3$ with an RMS deviation of 0.091 $e^-/Å^3$. On the basis of the final model, the calculated density was 1.602 g/cm^3 and F(000), 744 e^-.

[Ag(2,2'-bipy)(dppe)][CF₃SO₃] (**4**)

A total of 1270 frames were collected. The integration of the data using a monoclinic unit cell yielded a total of 169764 reflections to a maximum θ angle of 30.43° (0.70 Å resolution), of which 12234 were independent (average redundancy 13.876, completeness = 99.8%, R_{int} = 5.92%, R_{sig} = 2.56%) and 9470 (77.41%) were greater than $2\sigma(F^2)$. The final cell constants of a = 13.807(9) Å, b = 21.470(16) Å, c = 13.882(11) Å, β = 101.11(3)°, volume = 4038.(5) $Å^3$, are based upon the refinement of the XYZ-centroids of 9626 reflections above 20 σ(I) with 6.295° < 2θ < 60.69°. Data were corrected for absorption effects using the Multi-Scan method (SADABS). The ratio of minimum to maximum apparent transmission was 0.867. The calculated minimum and maximum transmission coefficients (based on crystal size) are 0.6471 and 0.7461. Refinement using the space group P 1 21/n 1, with Z = 4 for the formula unit, $C_{38}H_{33}AgCl_3F_3N_2O_3P_2S$. The final anisotropic full-matrix least-squares refinement on F^2 with 479 variables converged at R1 = 4.97%, for the observed data and wR2 = 15.38% for all data. The goodness-of-fit was 1.144. The largest peak in the final difference electron density synthesis was 1.633 $e^-/Å^3$ and the largest hole was -1.969 $e^-/Å^3$ with an RMS deviation of 0.139 $e^-/Å^3$. On the basis of the final model, the calculated density was 1.531 g/cm^3 and F(000), 1880 e^-.

Table S1. Selected bond lengths (Å) and angles (°) for complexes **1-4**.

**1**		**2**			
Ag(1)-N(32)	2.283(2)	Ag(1)-N(32)	2.321(3)		
Ag(1)-N(21)	2.287(2)	Ag(1)-N(21)	2.300(2)		
Ag(1)-P(2)	2.3475(12)	Ag(1)-P(2)	2.3483(16)		
		Ag(1)-O(3’)	2.501(14)		
N(32)-Ag(1)-N(21)	72.73(7)	N(32)-Ag(1)-N(21)	71.88(8)		
N(21)-Ag(1)-P(2)	138.99(5)	N(21)-Ag(1)-P(2)	142.58(6)		
N(32)-Ag(1)-P(2)	146.53(5)	N(32)-Ag(1)-P(2)	132.85(7)		
		O(3’)-Ag(1)-P(2)	105.3(2)		
		N(21)-Ag(1)-O(3’)	99.4(3)		
		N(32)-Ag(1)-O(3’)	93.3(2)		
**3**		**4**			
Ag(1)-N(2)	2.2587(19)	Ag(1)-N(13)	2.384(3)	P(14)-C(27)	1.846(3)
Ag(1)-N(13)	2.2746(18)	Ag(1)-N(2)	2.433(3)	P(28)-C(41)	1.842(3)
Ag(1)-P(18)	2.3365(14)	Ag(1)-P(14)	2.4302(15)	C(27)-C(27)	1.541(6)
		Ag(1)-P(28)	2.4325(18)	C(41)-C(41)	1.528(6)
N(2)-Ag(1)-N(13)	73.27(7)	N(32)-Ag(1)-N(21)	71.88(8)	N(13)-Ag(1)-N(2)	69.18(11)
N(2)-Ag(1)-P(18)	139.02(5)	N(21)-Ag(1)-P(2)	142.58(6)	N(13)-Ag(1)-P(14)	111.41(8)
N(13)-Ag(1)-P(18)	145.32(5)	N(32)-Ag(1)-P(2)	132.85(7)	N(13)-Ag(1)-P(28)	107.17(8)
		O(3’)-Ag(1)-P(2)	105.3(2)	N(2)-Ag(1)-P(14)	108.67(9)
		N(21)-Ag(1)-O(3’)	99.4(3)	N(2)-Ag(1)-P(28)	117.02(8)
		N(32)-Ag(1)-O(3’)	93.3(2)	P(14)-Ag(1)-P(28)	127.95(4)

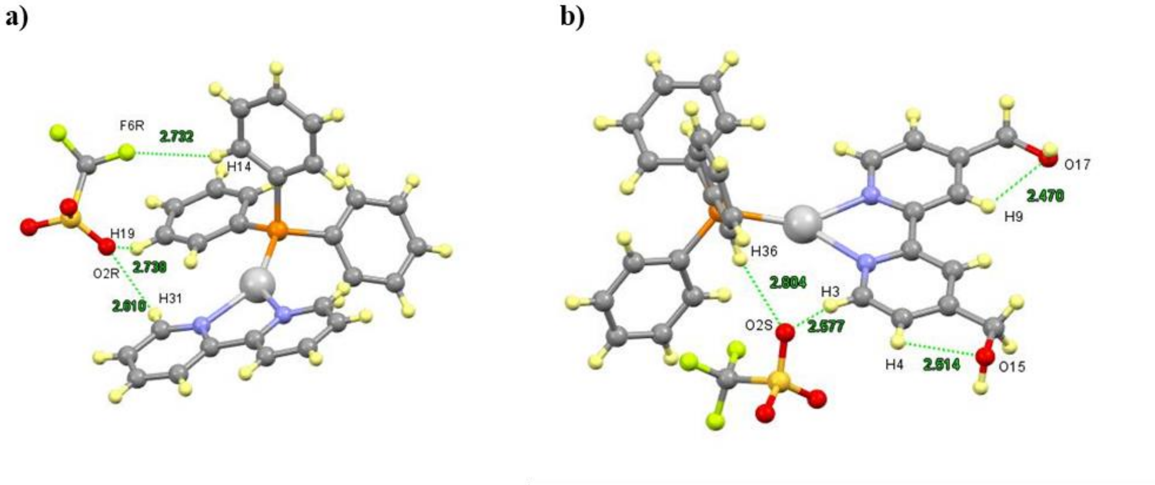

Figure S24. X-ray structure with the intramolecular hydrogen bonds observed in **1** (a) and **3** (b) complexes.

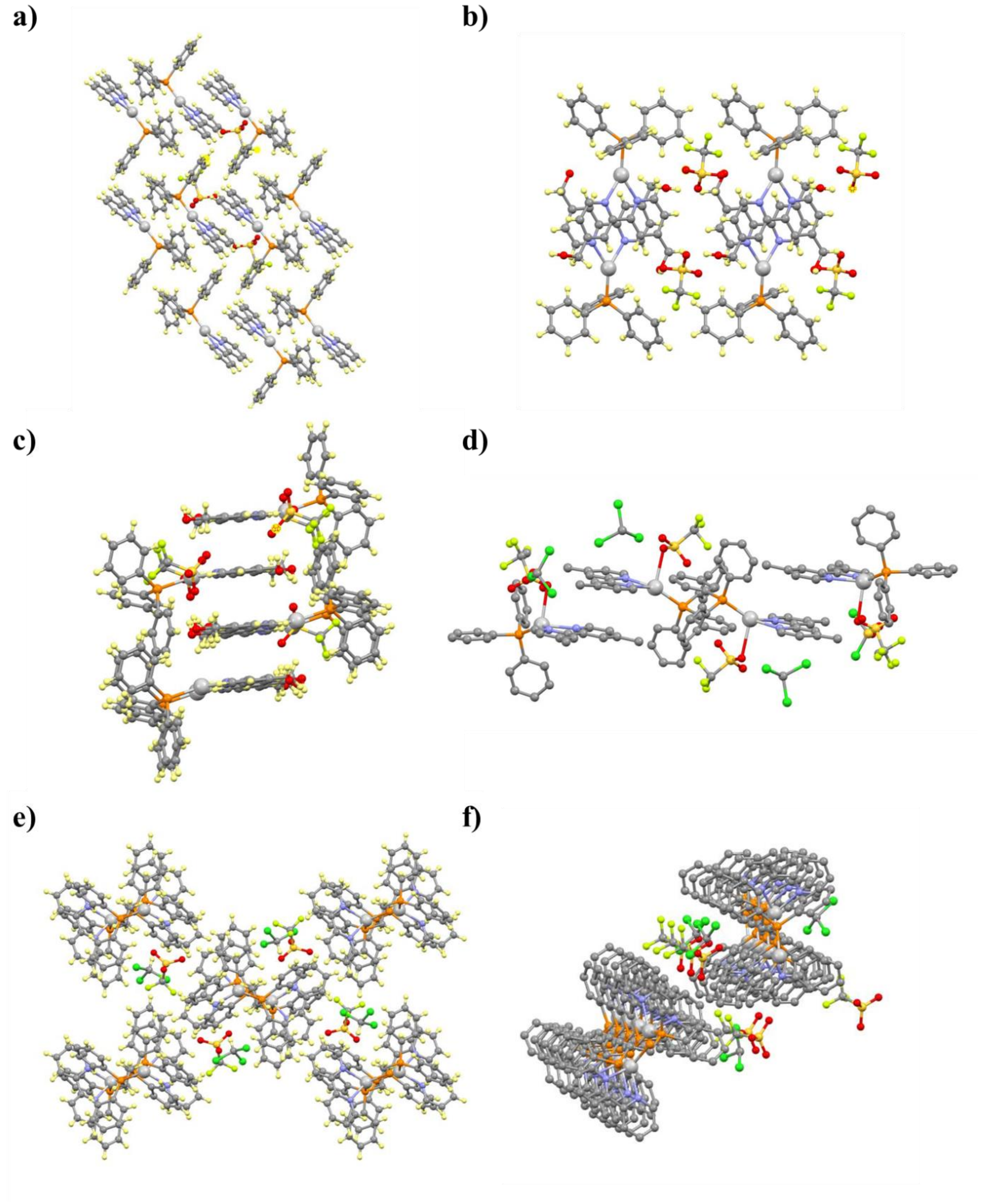

Figure S25. Packing diagram along the *a* axis for complexes **1** (a), **3** (b), **2** (d), **4** (e). Another vision of the packing diagrams for **3** (c) and **4** (f) complexes.

Solution behavior of compounds **1** and **2** in dmso-d_6

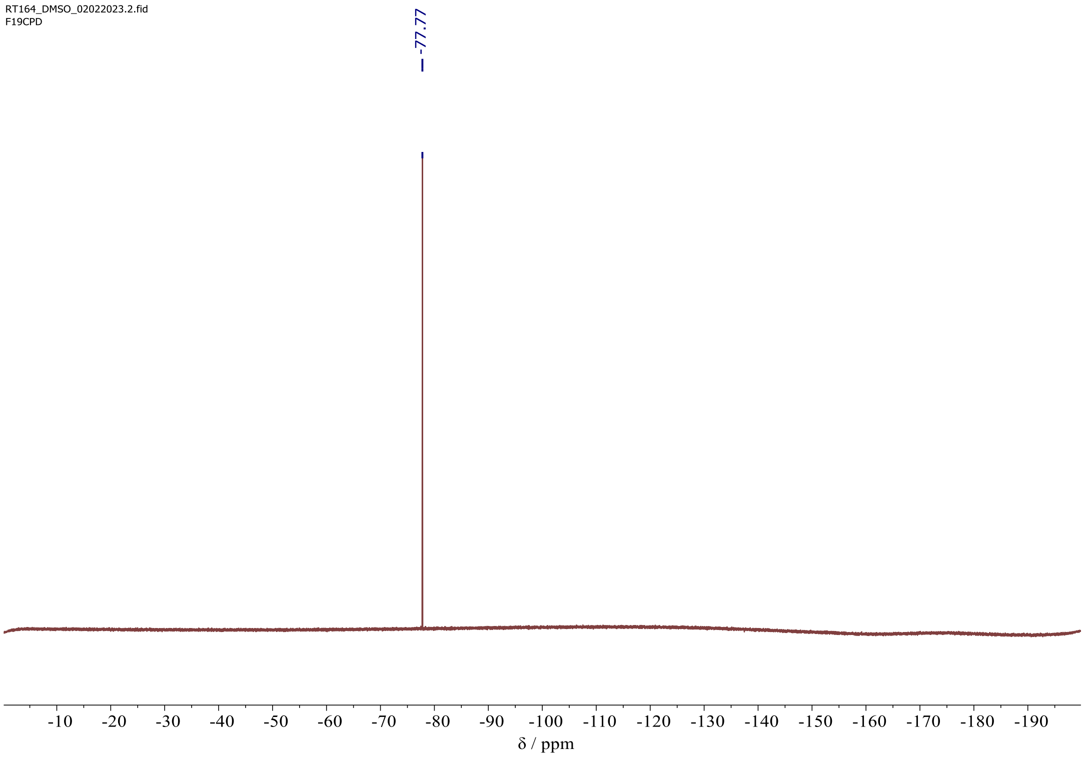

Figure S26. $^{19}F\{^1H\}$ NMR spectrum of compound **1** in DMSO-d_6 at 298 K.

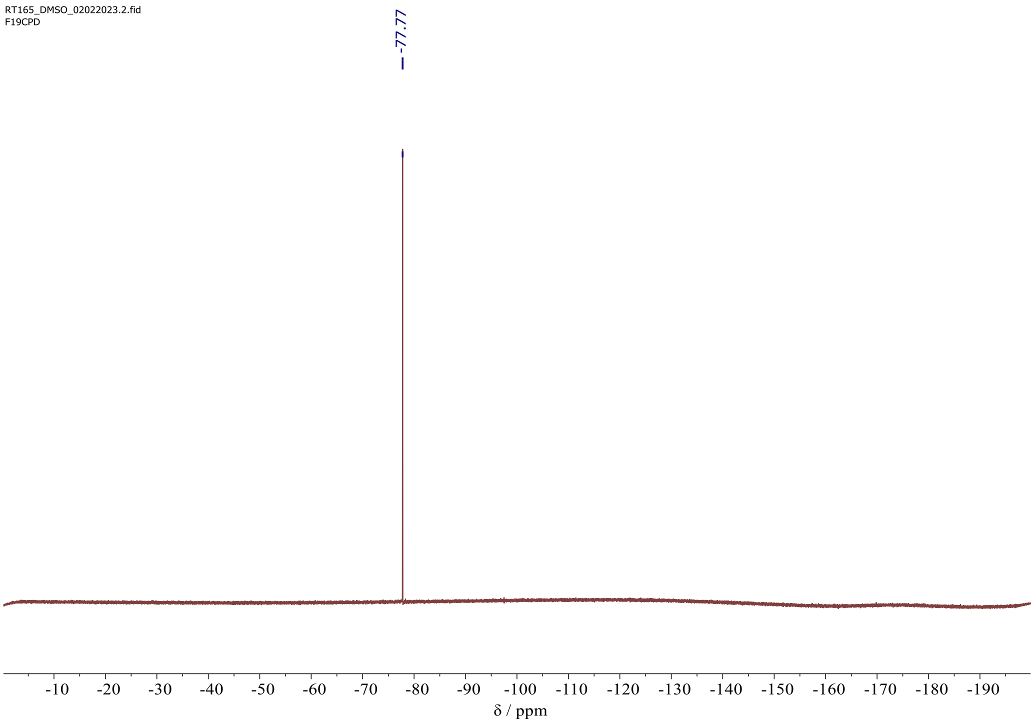

Figure S27. $^{19}F\{^1H\}$ NMR spectrum of compound **2** in DMSO-d_6 at 298 K.

S4. Stability studies

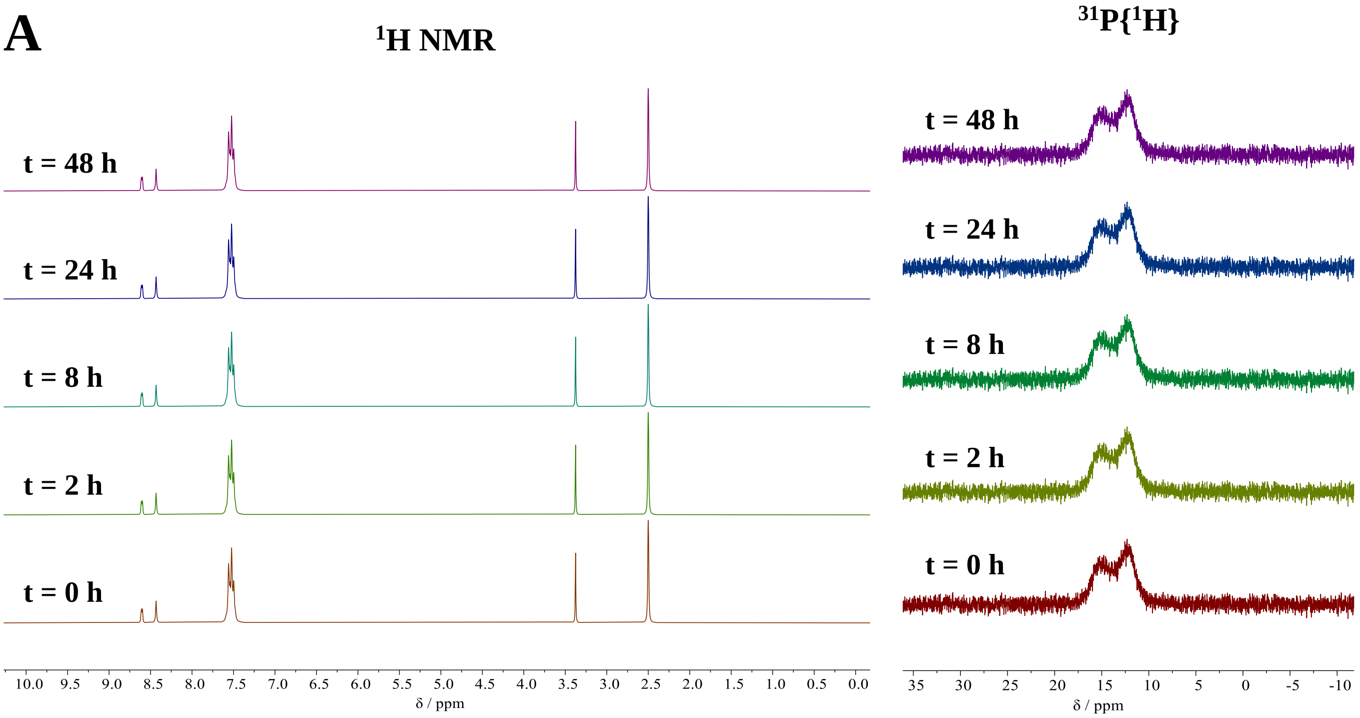

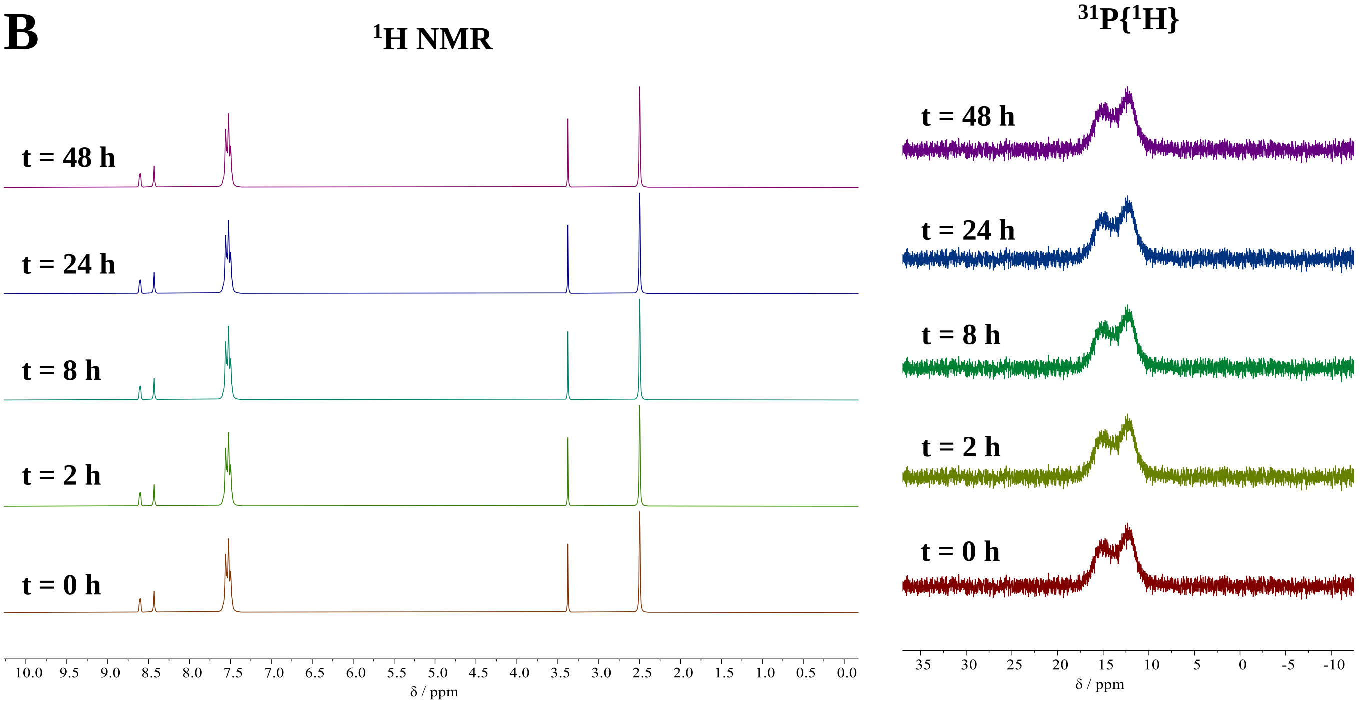

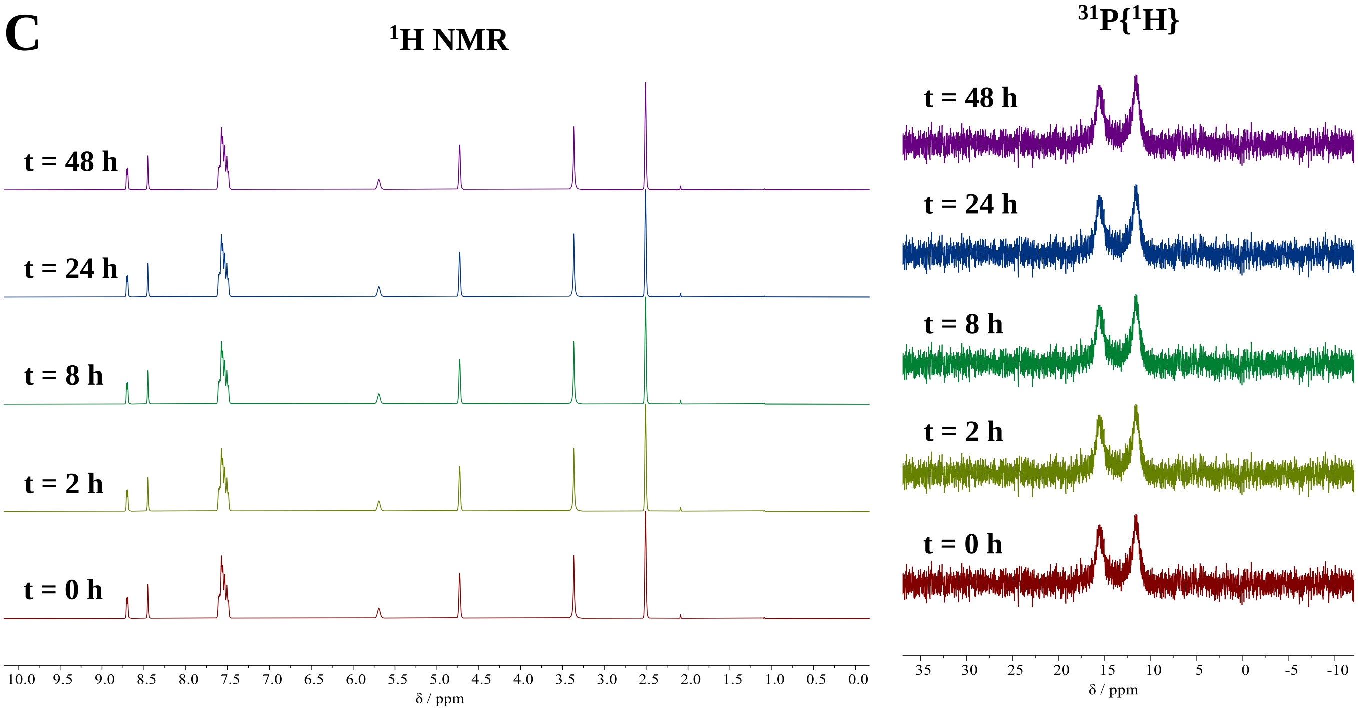

Figure S28. Stability studies monitored by ^{1}H and $^{31}P\{^{1}H\}$ NMR in DMSO-d_6 stock solutions for PPh_3-containing compounds **1** (A), **2** (B), and **3** (C) over 48 h.

A

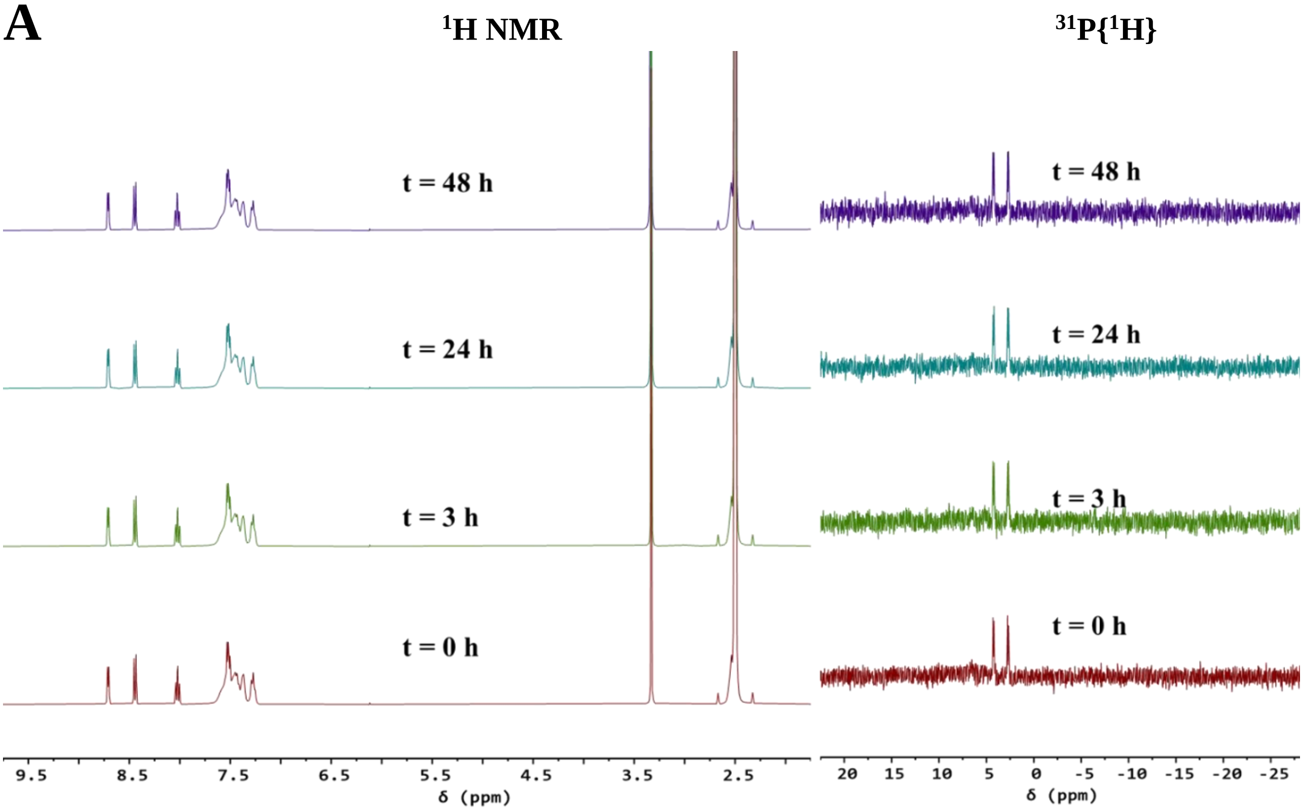

^{1}H NMR
^{31}P{^{1}H}
t = 48 h
t = 24 h
t = 3 h
t = 0 h
t = 48 h
t = 24 h
t = 3 h
t = 0 h
9.5 8.5 7.5 6.5 5.5 4.5 3.5 2.5
δ (ppm)
20 15 10 5 0 -5 -10 -15 -20 -25
δ (ppm)

B

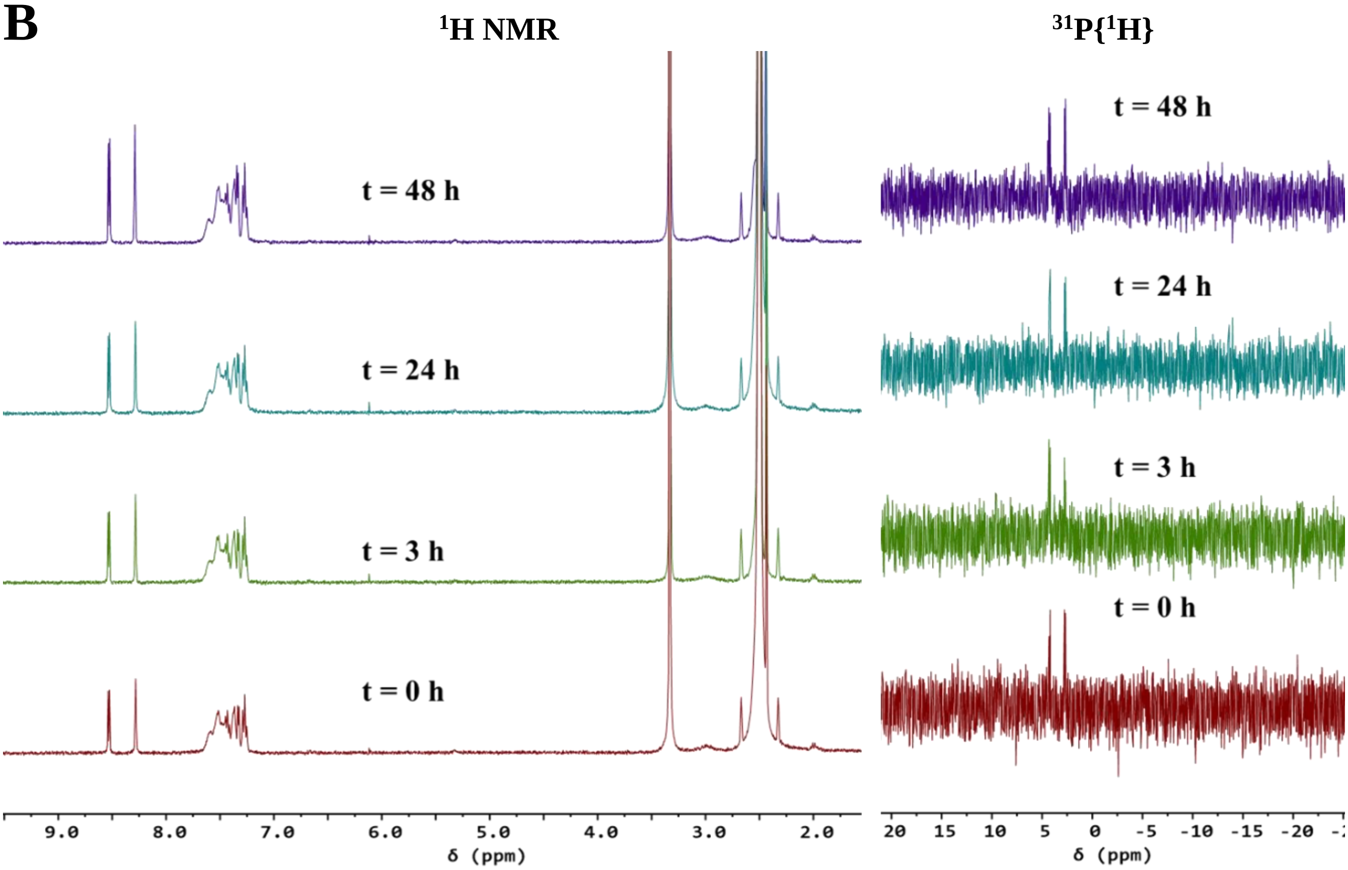

^{1}H NMR
^{31}P{^{1}H}
t = 48 h
t = 24 h
t = 3 h
t = 0 h
t = 48 h
t = 24 h
t = 3 h
t = 0 h
9.0 8.0 7.0 6.0 5.0 4.0 3.0 2.0
δ (ppm)
20 15 10 5 0 -5 -10 -15 -20
δ (ppm)

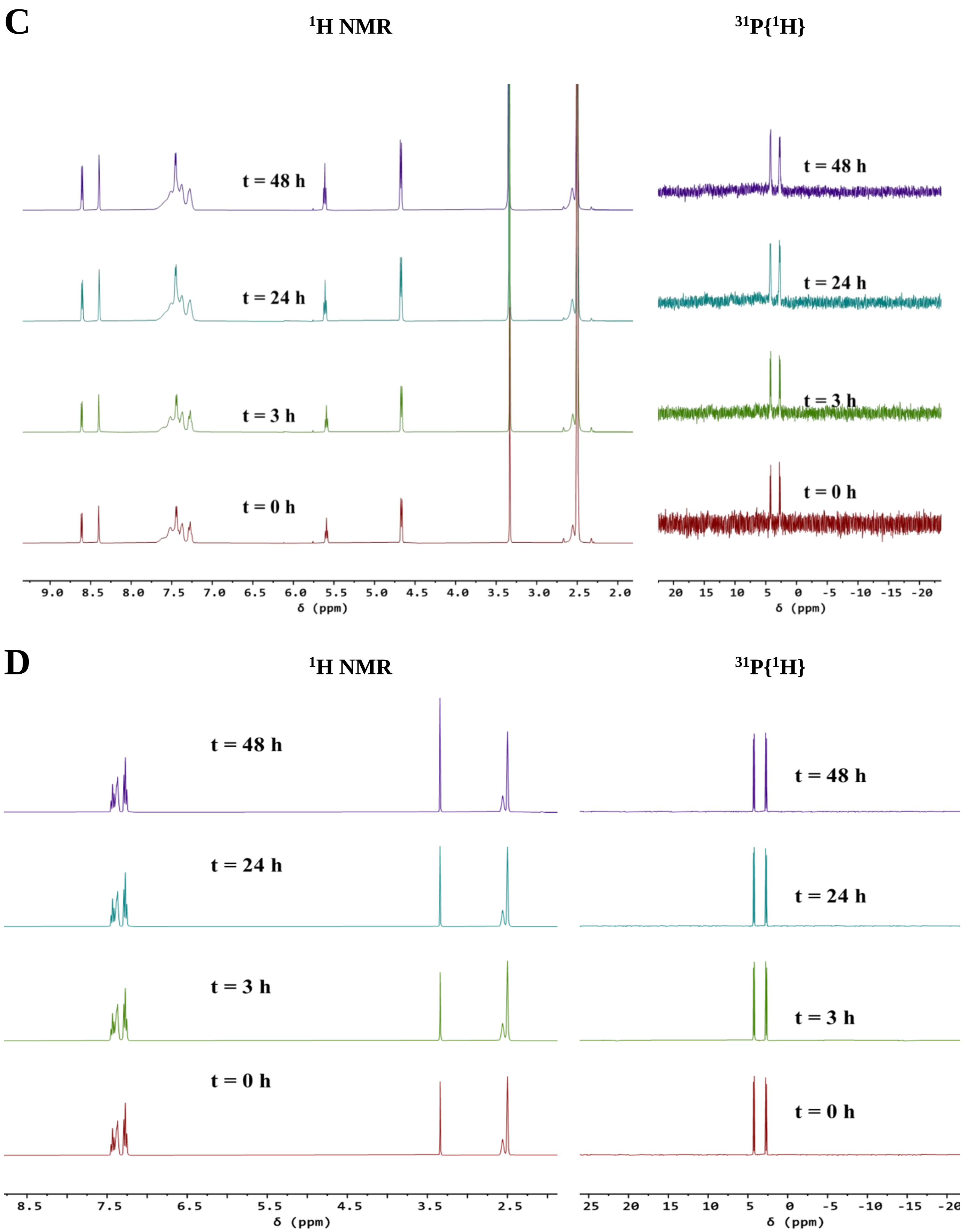

Figure S29. Stability studies monitored by ^{1}H and ^{31}P{^{1}H} NMR in DMSO-d_6 stock solutions for dppe-containing compounds **4** (A), **5** (B), **6** (C), and **7** (D) over 48 h.

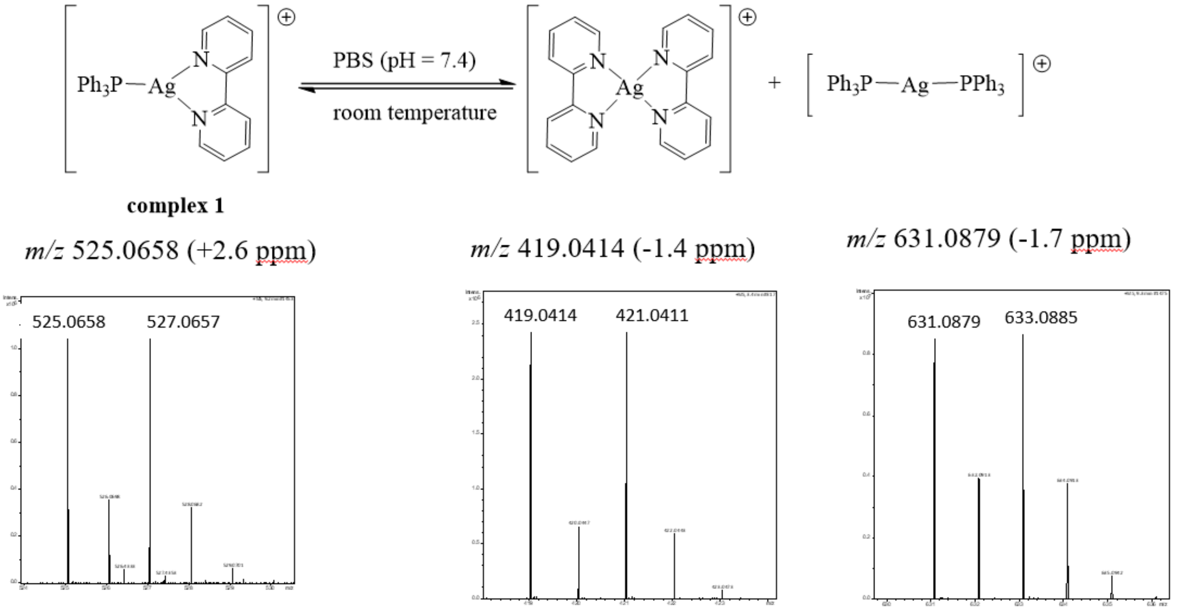

Figure S30. LC-ESI(+)-HRMS analysis of compound **1** in PBS (80 μM).

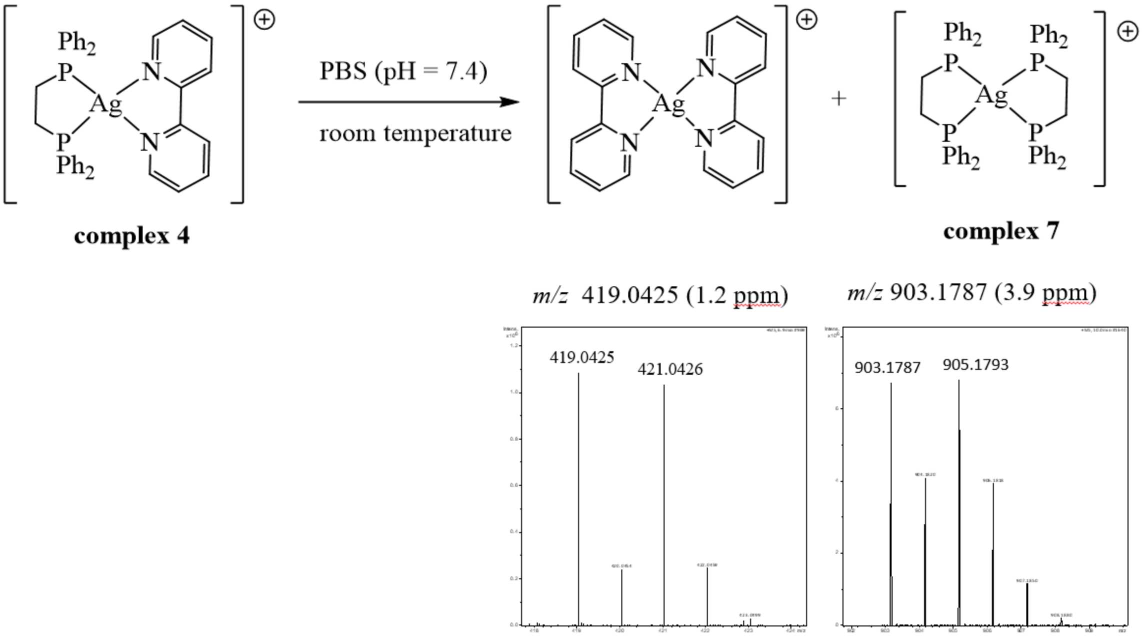

Figure S31. LC-ESI(+)-HRMS analysis of compound **4** in PBS (80 μM).

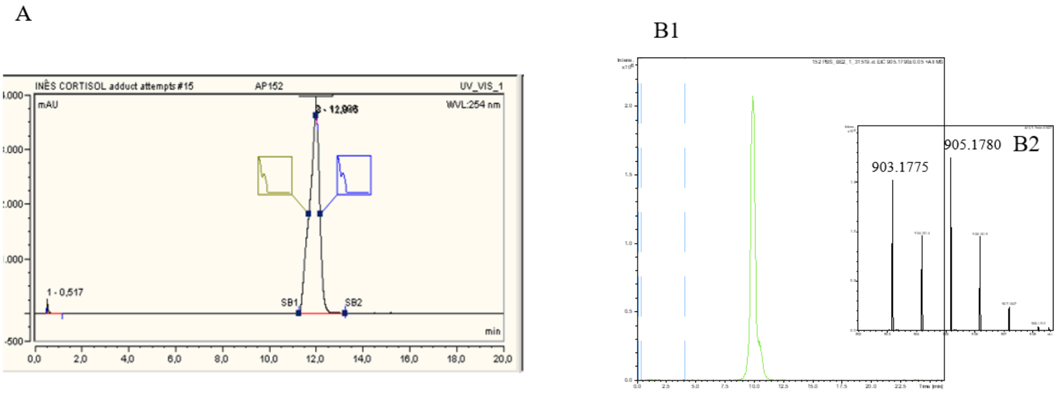

Figure S32. A. LC-DAD chromatogram obtained for compound 7 in MeCN**; B1**. LC-ESI(+)-HRMS analysis extracted ion chromatogram at *m/z* 903.1781, obtained for **7** in PBS (80 µM), following 24 h incubation at 37ºC; and **B2** .Corresponding HRMS full scan spectrum

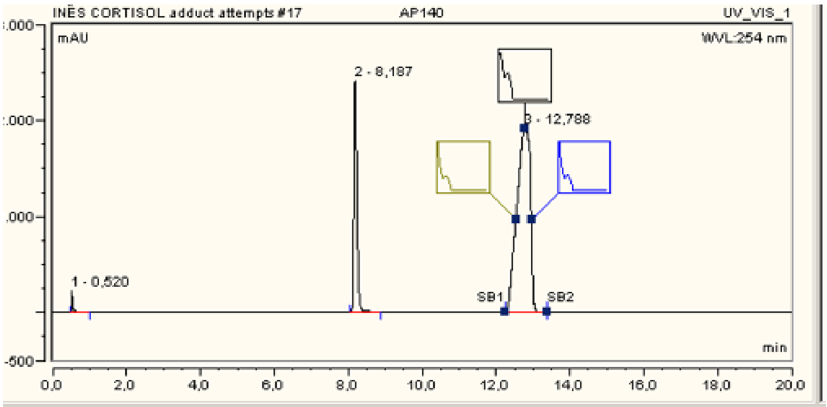

Figure S33. A. LC-DAD chromatogram obtained for compound **4** in MeCN: showing the the two complexes identified in Figure S31.

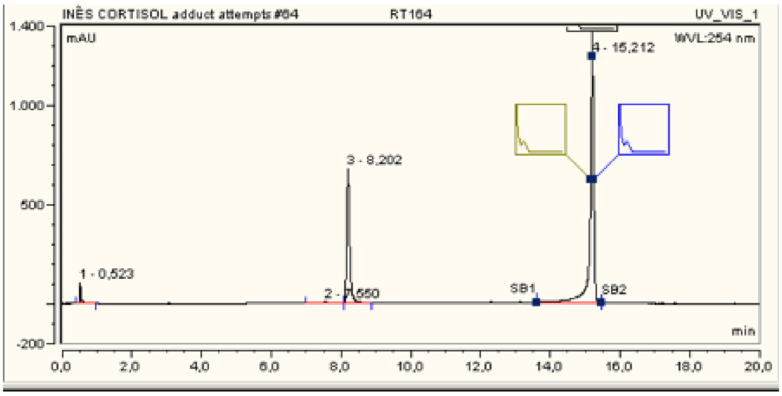

Figure S34. A. LC-DAD chromatogram obtained for compound **1** in MeCN: showing the two complexes identified in Figure S30.

S5. Biological data

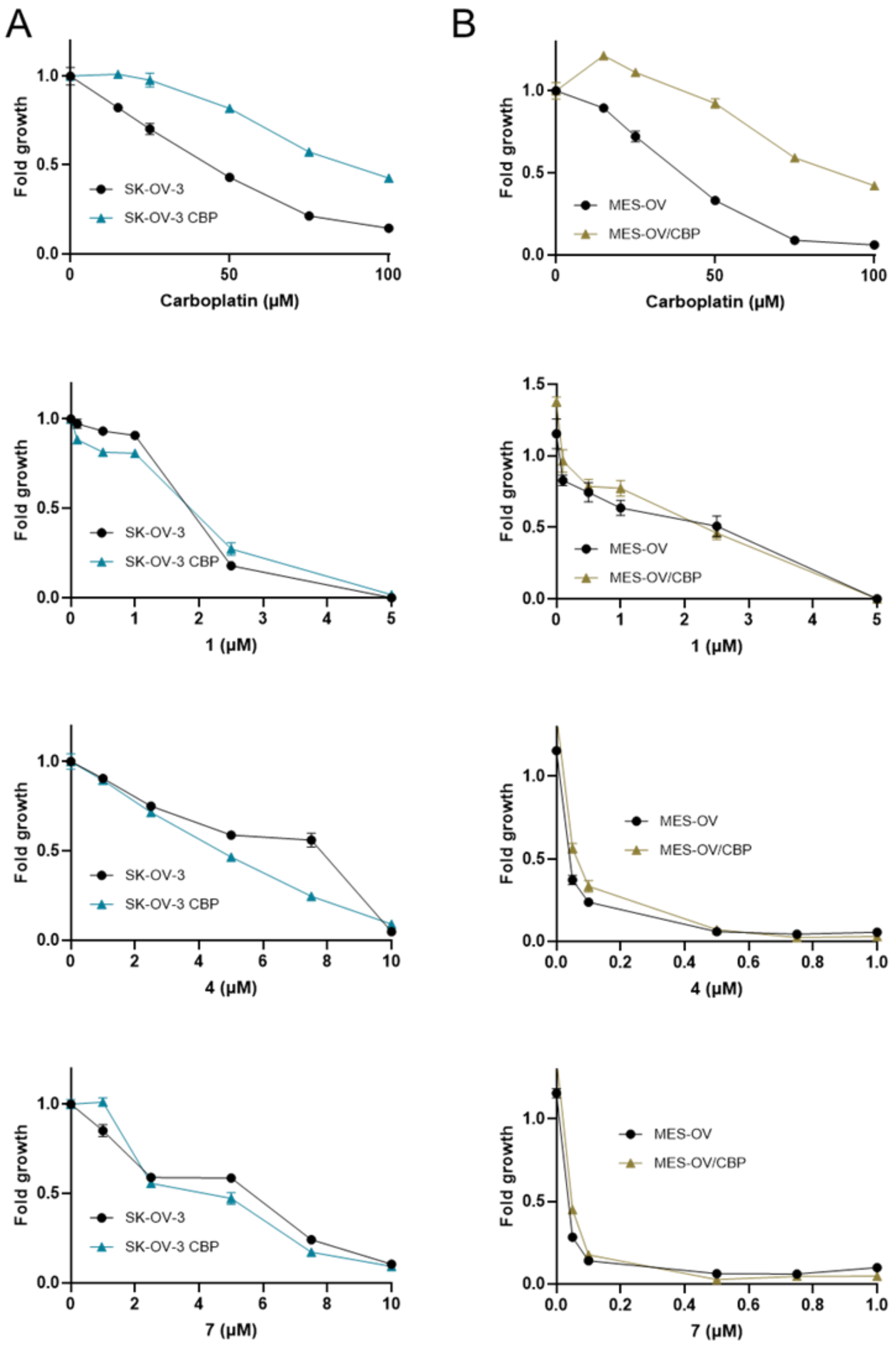

Figure S35. Impact of carboplatin resistance mechanism on the anticancer activity of **1**, **4** and **7** in comparison to carboplatin. SK-OV-3/CBP (A) and MES-OV/CBP (B) cells compared to their parental cell lines. Cytotoxicity of the drugs was determined by MTT assay after 72 h of treatment. A representative graph of one experiment with the mean values of triplicates is shown.

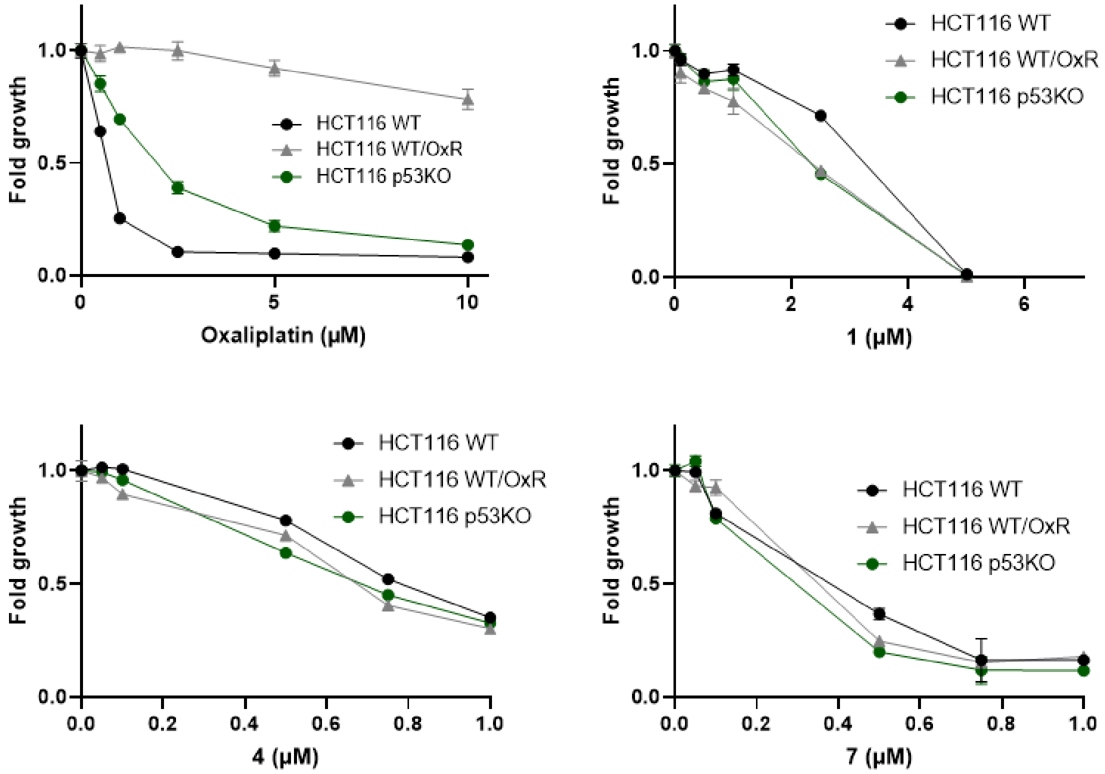

Figure S36. Impact of oxaliplatin resistance on **1**, **4** and **7** in comparison to oxaliplatin. HCT116 subclones were treated with increasing concentrations of the test compounds and the cytotoxicity was evaluated by MTT assays after 72 h. The graph shows dose-response curves of one representative experiment performed in triplicates.

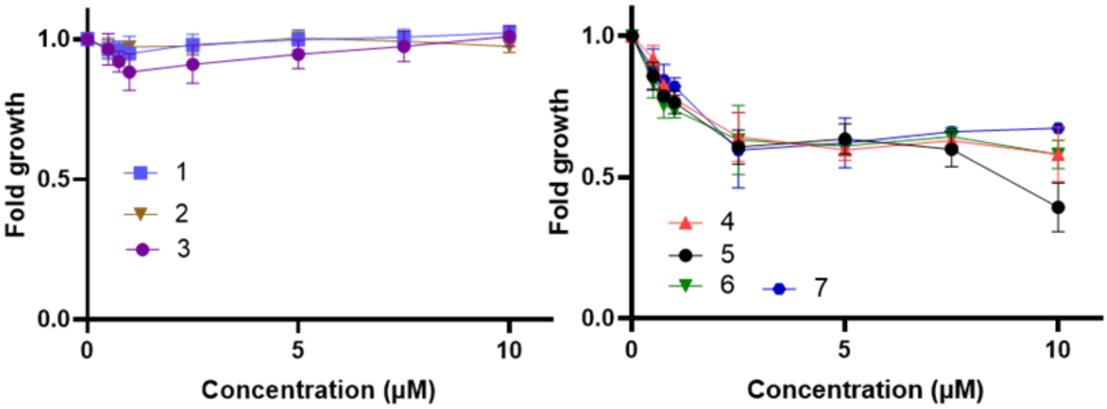

Figure S37. Anticancer activity of novel silver compounds in colonic epithelial fibroblasts. F331 cells were treated with increasing concentrations of the compounds for 72 h, followed by MTT assay. The data represented in the graphs are the mean ± SD of three independent experiments, each performed in technical triplicates.

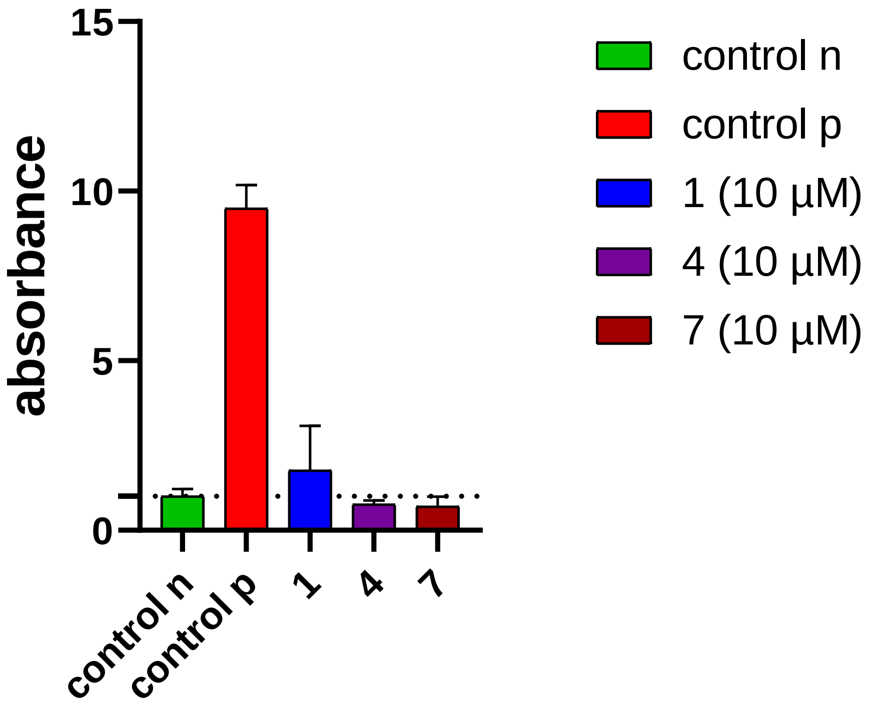

Figure S38. *In vitro* hemolytic activity of the tested silver drugs. The mean ± standard deviation (SD) was derived from triplicates of two representative experiments.

Table S2. Anticancer activity (IC_{50} values after 24 and 72 h) of the indicated compounds in SKOV-3 and MES-OV OC cells.

Time (h)		(IC50, μM ± SD)[a]	
		SK-OV-3	MES-OV
1	24 h	3.6 ± 0.7	2.84 ± 0.9
	72 h	2.1 ± 0.3	2.60 ± 0.2
4	24 h	7.9 ± 0.7	7.61 ± 0.6
	72 h	6.8 ± 1.4	< 0.05
7	24 h	8.3 ± 0.3	4.39 ± 0.3
	72 h	7.2 ± 1.4	< 0.05

[a] IC_{50} values were calculated from concentration-response curves. Values are given as mean ± SD of three independent experiments performed in triplicates.

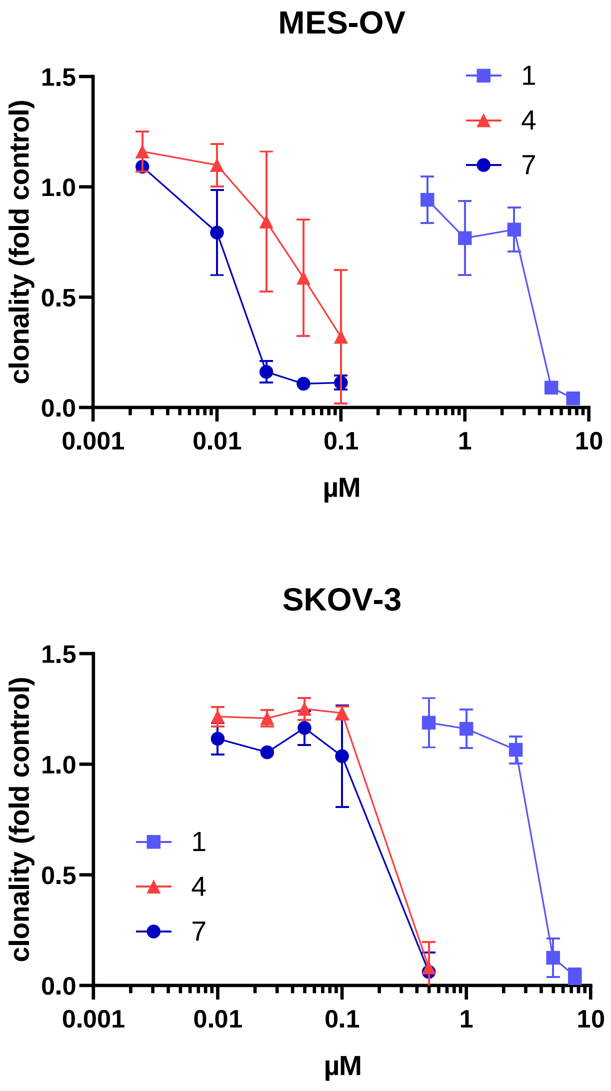

Figure S39. Cytotoxicity after long-term treatment of MES-OV and SKO-OV-3 cells with the tested compounds. Cells were fixed and stained with crystal violet after 10 days. Fold growth to untreated cells was calculated from densitometric analysis performed with Image J. Values given are the mean ± SD of duplicates from two experiments.

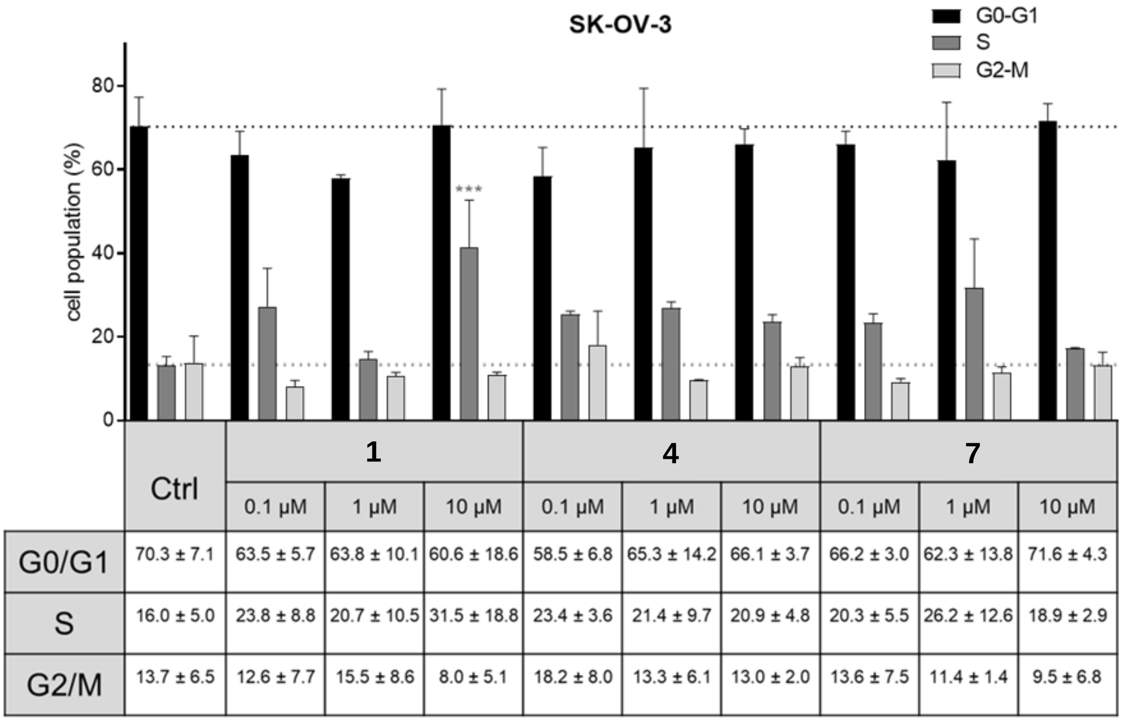

	Ctrl	1			4			7		
		0.1 μM	1 μM	10 μM	0.1 μM	1 μM	10 μM	0.1 μM	1 μM	10 μM
G0/G1	70.3 ± 7.1	63.5 ± 5.7	63.8 ± 10.1	60.6 ± 18.6	58.5 ± 6.8	65.3 ± 14.2	66.1 ± 3.7	66.2 ± 3.0	62.3 ± 13.8	71.6 ± 4.3
S	16.0 ± 5.0	23.8 ± 8.8	20.7 ± 10.5	31.5 ± 18.8	23.4 ± 3.6	21.4 ± 9.7	20.9 ± 4.8	20.3 ± 5.5	26.2 ± 12.6	18.9 ± 2.9
G2/M	13.7 ± 6.5	12.6 ± 7.7	15.5 ± 8.6	8.0 ± 5.1	18.2 ± 8.0	13.3 ± 6.1	13.0 ± 2.0	13.6 ± 7.5	11.4 ± 1.4	9.5 ± 6.8

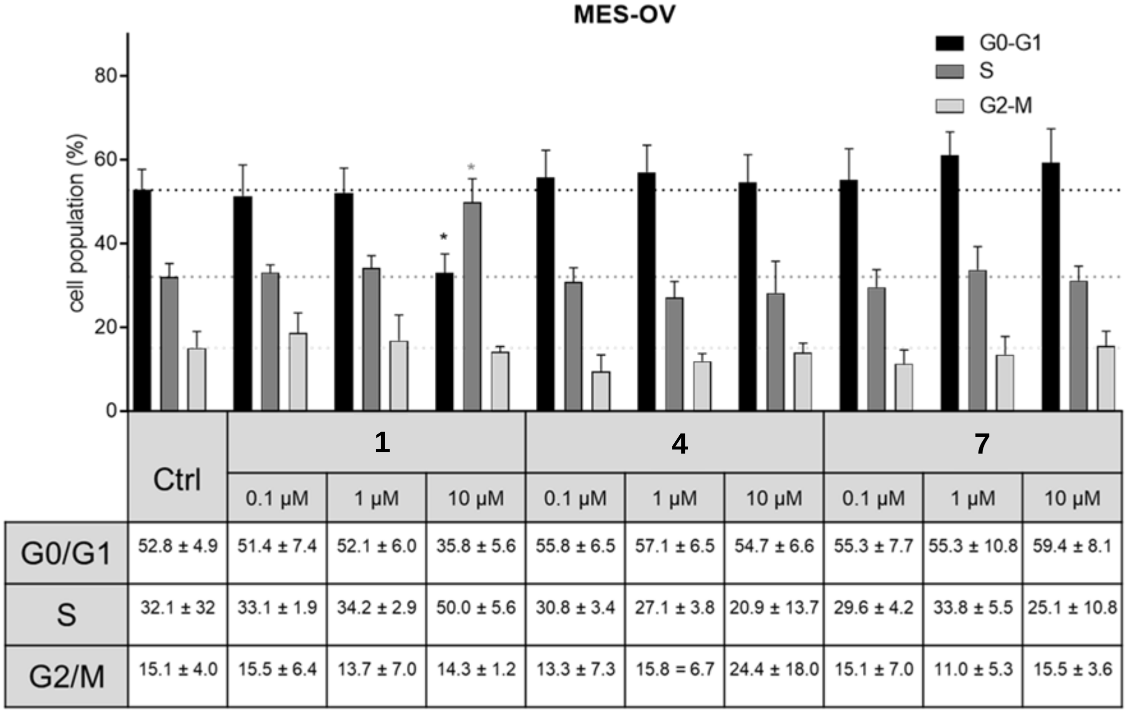

	Ctrl	1			4			7		
		0.1 μM	1 μM	10 μM	0.1 μM	1 μM	10 μM	0.1 μM	1 μM	10 μM
G0/G1	52.8 ± 4.9	51.4 ± 7.4	52.1 ± 6.0	35.8 ± 5.6	55.8 ± 6.5	57.1 ± 6.5	54.7 ± 6.6	55.3 ± 7.7	55.3 ± 10.8	59.4 ± 8.1
S	32.1 ± 32	33.1 ± 1.9	34.2 ± 2.9	50.0 ± 5.6	30.8 ± 3.4	27.1 ± 3.8	20.9 ± 13.7	29.6 ± 4.2	33.8 ± 5.5	25.1 ± 10.8
G2/M	15.1 ± 4.0	15.5 ± 6.4	13.7 ± 7.0	14.3 ± 1.2	13.3 ± 7.3	15.8 = 6.7	24.4 ± 18.0	15.1 ± 7.0	11.0 ± 5.3	15.5 ± 3.6

Figure S40. Impact of the tested compounds on cell cycle distribution of SK-OV-3 (A) and MES-OV (B) cells after 24 h of treatment. The percentage of cells in G0-G1, S and G2/M phase were analyzed in ethanol-fixed cells with propidium iodide (PI) followed by flow cytometry after 24 h treatment. The mean ± SD was derived from three independent experiments.

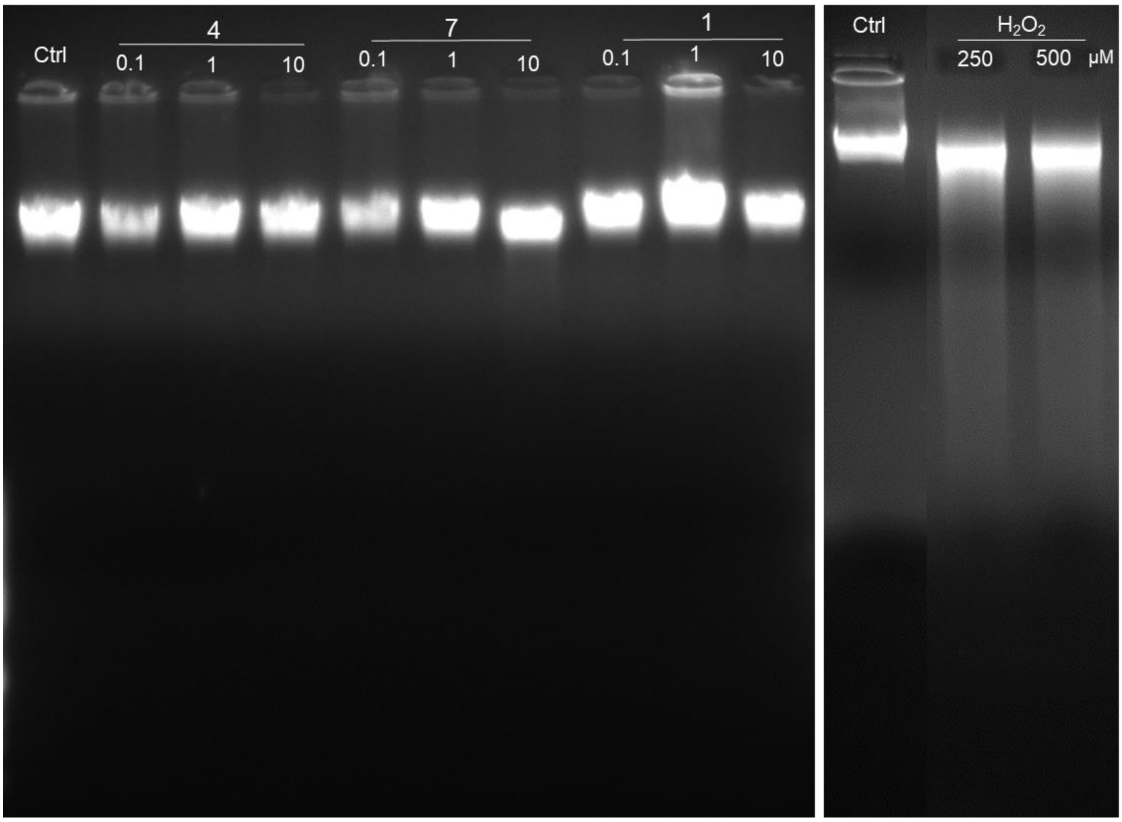

Figure S41. "DNA ladder" pattern of DNA fragments occurring during apoptosis was evaluated after 24h treatment with the indicated concentrations of silver compounds. As positive control 250 and 500 µM H_2O_2 was used to treat the cells for 24 h.

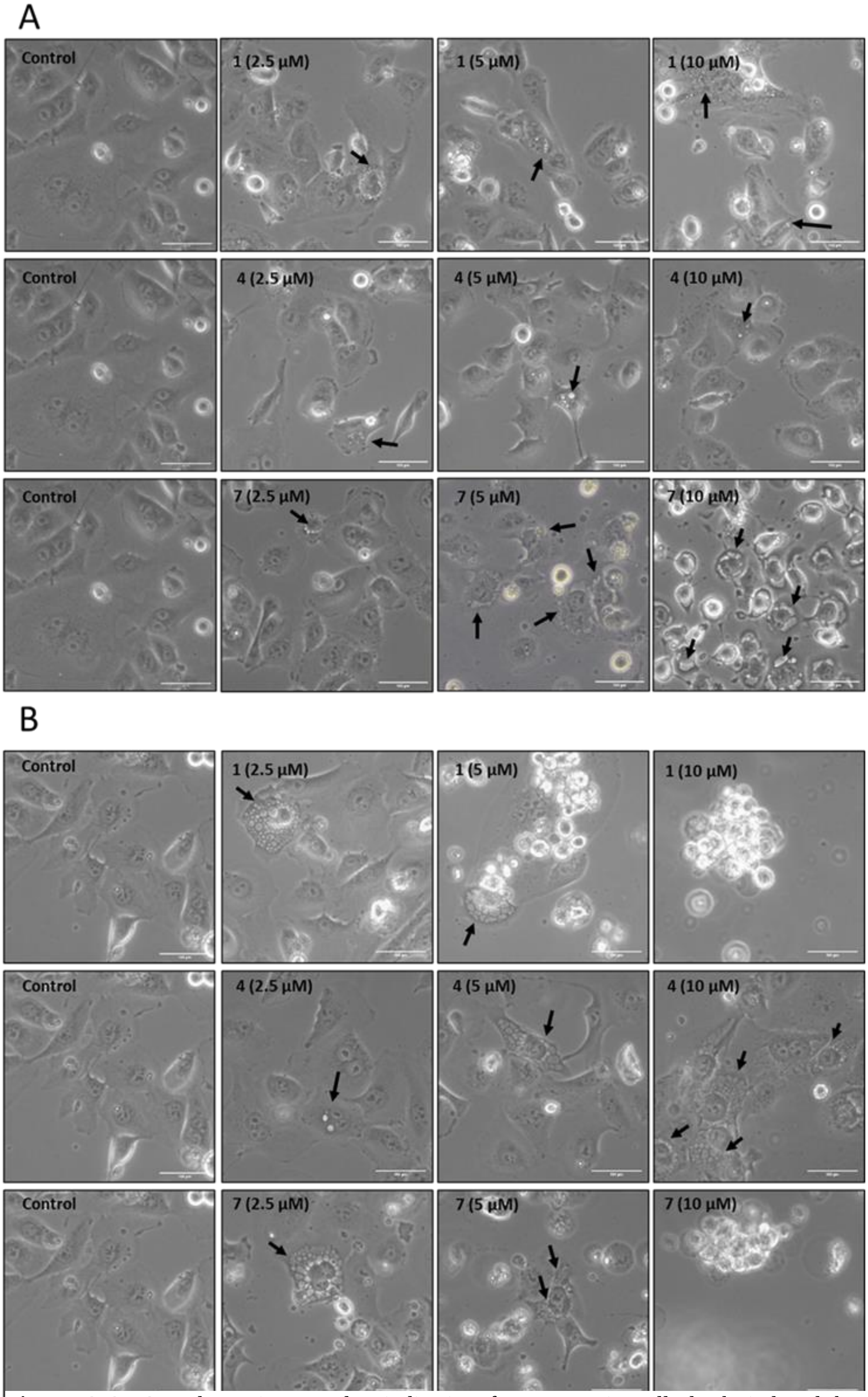

Figure S42. Cytoplasmatic vacuoles indicative for paraptotic cell death induced by silver compounds treatment. Phase-contrast images of MES-OV cells treated with the indicated compounds for 6 h (A) and 24 h (B) (200× magnification, scale bar: 100 µm).

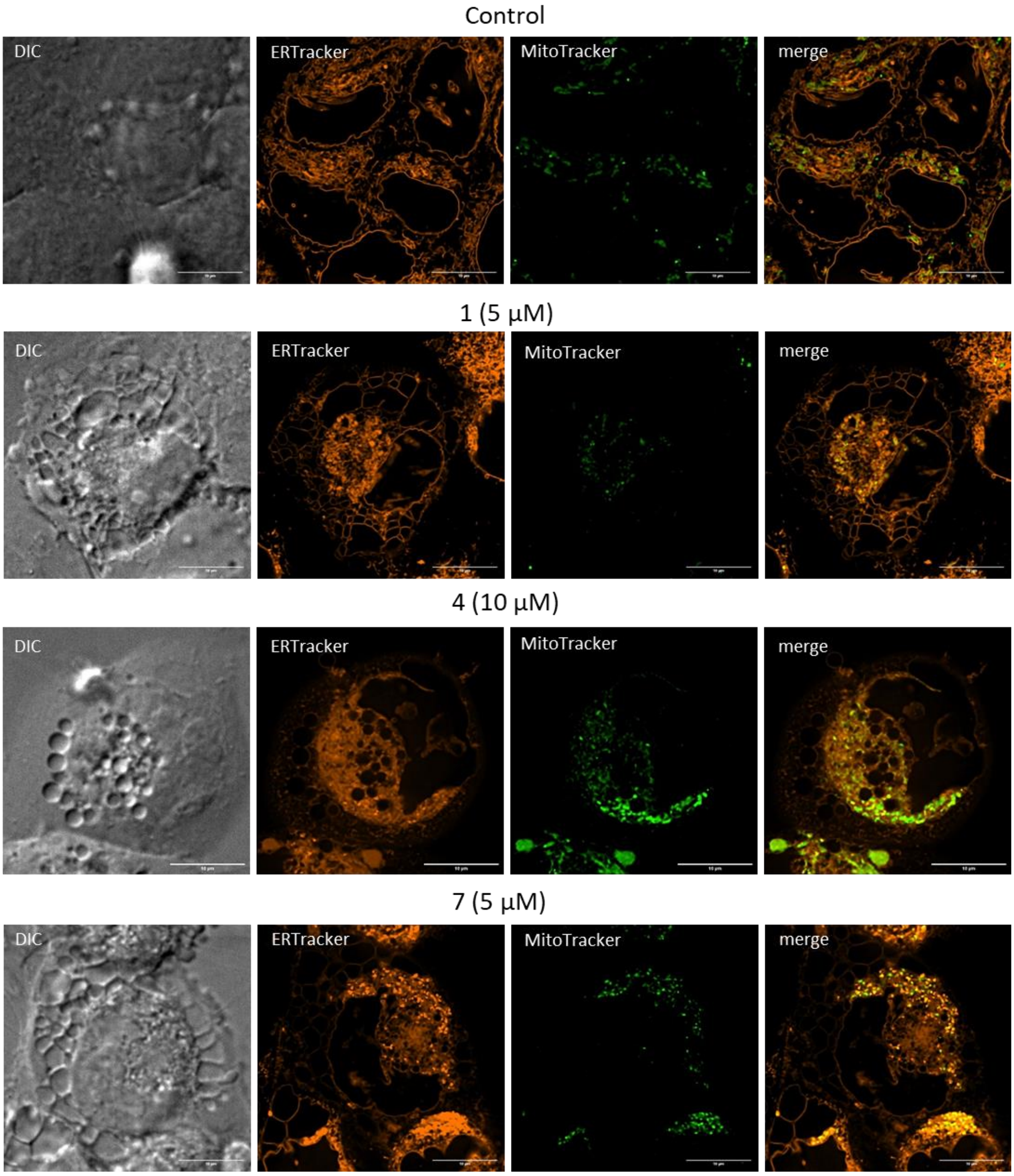

Figure S43. Spinning disk confocal microscopy of MES-OV cells treated with the indicated compounds and concentrations for 24 h. Representative pictures were taken in confocal mode, Z-stack and max intensity projection (192x magnification and 60x objectives 600-x magnification) of vesicles with stain of the ER (ERTracker in red), DIC (Differential interference contrast) and mitochondria (MitoTracker in green).

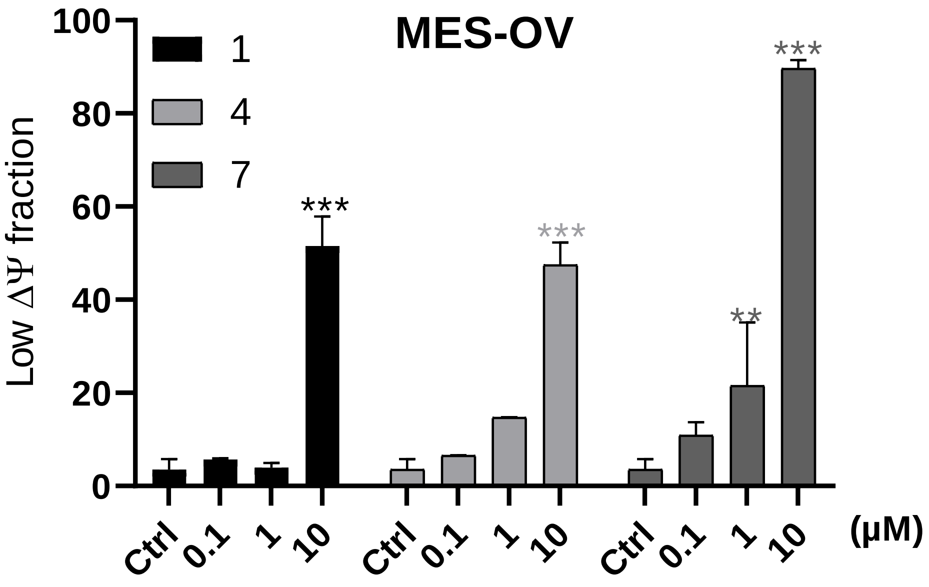

Figure S44. Mitochondrial damage after drug treatment in MES-OV cells. Cells were treated with the indicated concentrations of the novel silver drugs for 24 h. Mitochondrial membrane potential is given as the percentage of cells with decreased JC-1 fluorescence. The mean ± SD was derived from three independent experiments. Significance to control was calculated by two-way ANOVA and Bonferroni's multiple comparison test using GraphPad Prism software (***$p < 0.001$, **$p \leq 0.01$, *$p \leq 0.05$).

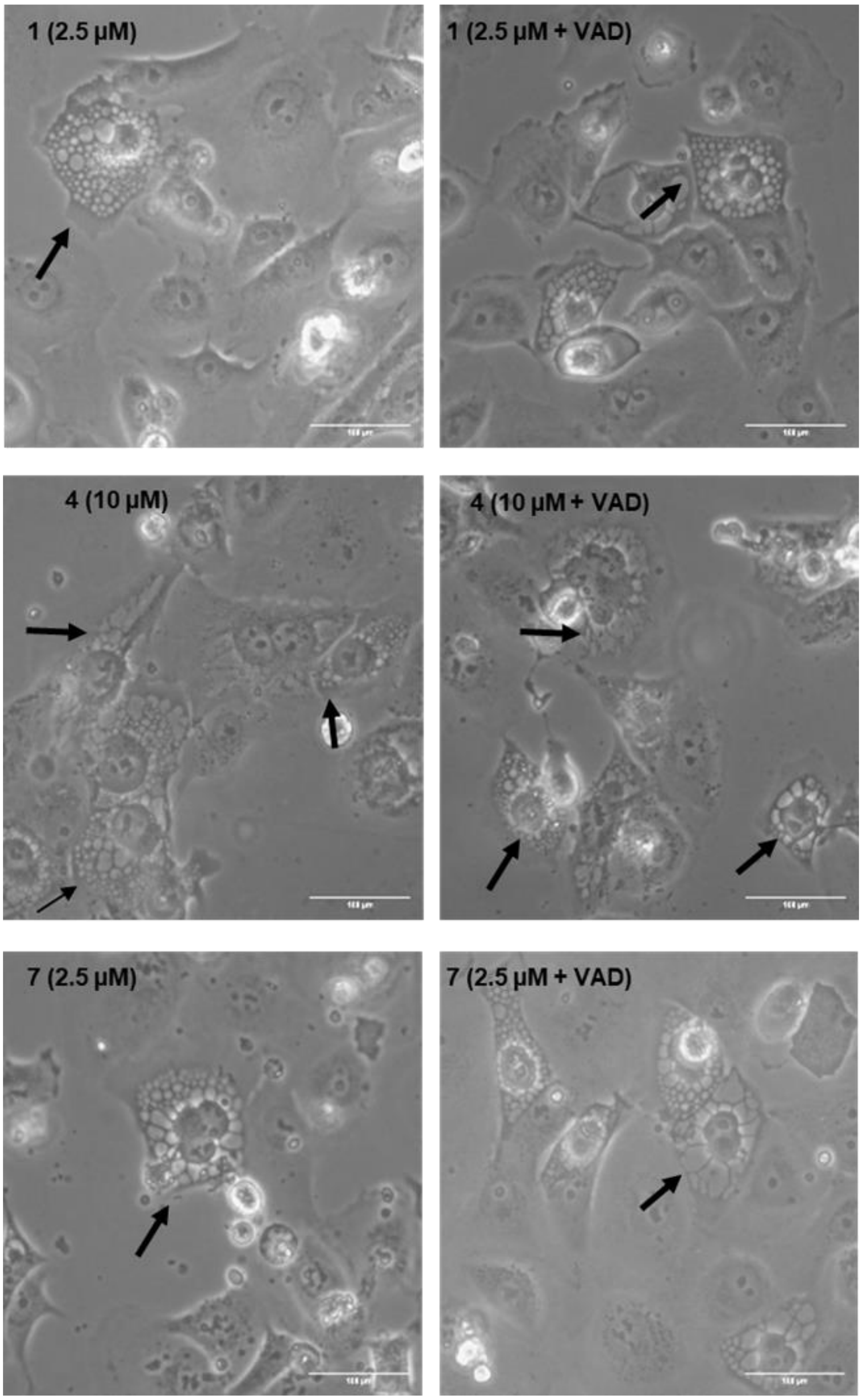

Figure S45. Impact of caspase inhibition on vacuole formation. Phase-contrast images of MES-OV cells treated with the indicated drugs in combination with and without the pan-caspase inhibitor Z-VAD-FMK for 24 h (200× magnification, scale bar: 100 µm).

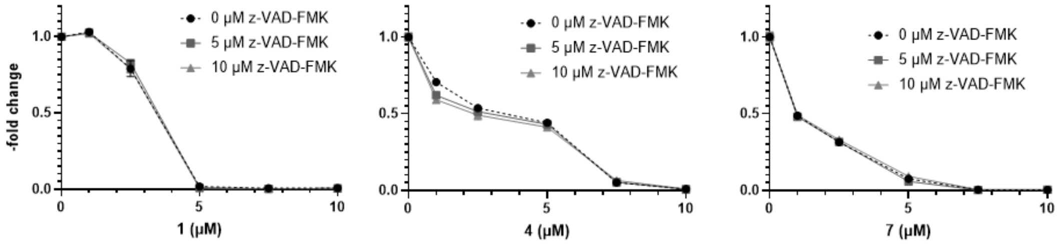

Figure S46. Impact of the pan caspase inhibitor z-VAD-FMK (5 µM and 10 µM) on the anticancer activity of the tested silver compounds in ER-YFP-transfected SW480 cells. In order to evaluate the cells´ viability, MTT assay was performed after 72 h of combined drug treatment. The mean ± SD was derived from triplicates of one representative experiment out of three.

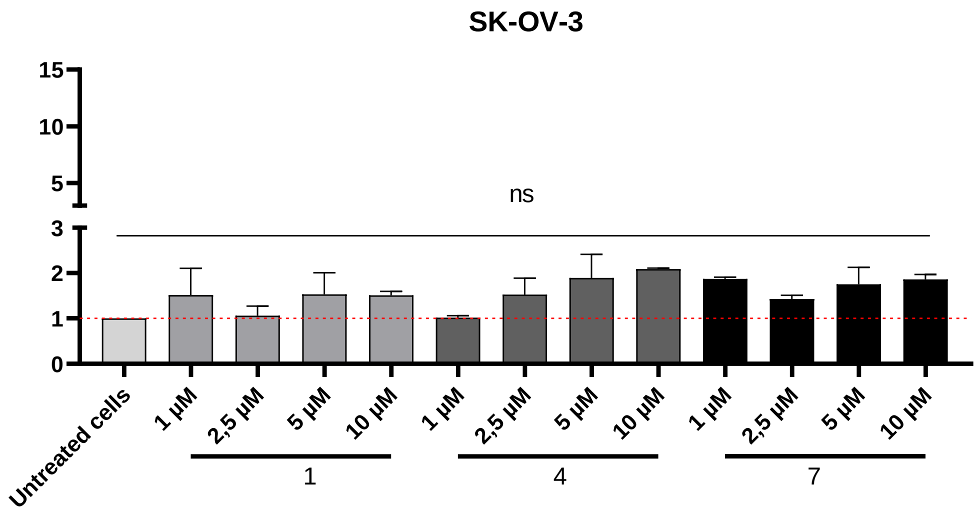

Figure S47. ROS production measured by flow cytometry of DCF-DA fluorescence in SK-OV-3 cells treated with indicated concentrations of the tested silver complexes after 24h. Significance to control was calculated with one-way ANOVA and Dunnett's multiple comparison test (ns ≤ 0.12)

Table S3: Human cell lines used in this study

Cell line	Origin	Specification	Medium	Source
SK-OV-3	Ovarian carcinoma	-	McCoy´s 5a	
SK-OV-3/CBP	Ovarian carcinoma	Carboplatin resistance	McCoy´s 5a	Established by Anamaria Brozovic[1]
MES-OV	Ovarian carcinoma	-	McCoy´s 5a	Donated to Anamaria Brozovic by Branimir I Sikic (Stanford University)[2]
MES-OV/CBP	Ovarian carcinoma	Carboplatin resistance	McCoy´s 5a	Established by Anamaria Brozovic[1]
F331	Human colorectal fibroblasts	-	Dulbecco´s Minimal Essential	donated by Brigitte Marian
HCT116 WT	Colorectal carcinoma	p53 – wild type	McCoy´s 5a	American Type Culture Collection (ATCC) (Rockville, MD, USA)
HCT116 WT/OxR	Colorectal carcinoma	Oxaliplatin resistance, p53 wild type	McCoy´s 5a	generated as previously published[3]
HCT116 p53KO	Colorectal carcinoma	p53 – knockout	McCoy´s 5a	Donated by Bert Vogelstein
SW480 ER-YFP	Colorectal carcinoma		MEME	generated as previously published[4]

Table S4. Experimental parameters for ICP-MS.

	**ICP-MS Agilent 7800**
**RF power (W)**	1550
**Cone material**	Nickel
**Carrier gas (L/min)**	1.08
**Plasma gas (L/min)**	15
**Monitored isotopes**	^{185}Re, ^{115}In, ^{107}Ag, ^{109}Ag
**Integration time [s]**	0.1
**Number of sweeps**	100
**Number of replicates**	10

References

(1) Kralj, J.; Pernar Kovač, M.; Dabelić, S.; Polančec, D.S.; Wachtmeister, T.; Köhrer, K.; Brozovic, A.; Transcriptome analysis of newly established carboplatin-resistant

ovarian cancer cell model reveals genes shared by drug resistance and drug-induced EMT. *Br. J. Cancer*. **2023**, 128 (7), 1344-1359.

(2) Wang, Y.C.; Wang, Y. C.; Juric, D.; Francisco, B; Yu, R. X.; Duran, G. E.; Chen, K. G.; Chen, X.; Sikic, B. I.; Regional activation of chromosomal arm 7q with and without gene amplification in taxane-selected human ovarian cancer cell lines. *Genes Chromosomes Cancer*, **2006**, 45, 365-374.

(3) Jungwirth, U.; Xanthos, D. N.; Gojo, J.; Bytzek, A. K.; Körner, W.; Heffeter, P.; Abramkin, S. A.; Jakupec, M. A.; Hartinger, C. G.; Windberger, U.; et al. Anticancer Activity of Methyl-Substituted Oxaliplatin Analogs. *Mol. Pharmacol.* **2012**, *81* (5), 719–728.

(4) Hager, S.; Korbula, K.; Bielec, B.; Grusch, M.; Pirker, C.; Schosserer, M.; Liendl, L.; Lang, M.; Grillari, J.; Nowikovsky, K.; et al. The Thiosemicarbazone Me2NNMe2 Induces Paraptosis by Disrupting the ER Thiol Redox Homeostasis Based on Protein Disulfide Isomerase Inhibition. *Cell Death Dis.* **2018**, *9* (11).
